# Common variants in *SOX-2* and congenital cataract genes contribute to age-related nuclear cataract

**DOI:** 10.1038/s42003-020-01421-2

**Published:** 2020-12-11

**Authors:** Ekaterina Yonova-Doing, Wanting Zhao, Robert P. Igo, Chaolong Wang, Periasamy Sundaresan, Kristine E. Lee, Gyungah R. Jun, Alexessander Couto Alves, Xiaoran Chai, Anita S. Y. Chan, Mei Chin Lee, Allan Fong, Ava G. Tan, Chiea Chuen Khor, Emily Y. Chew, Pirro G. Hysi, Qiao Fan, Jacqueline Chua, Jaeyoon Chung, Jiemin Liao, Johanna M. Colijn, Kathryn P. Burdon, Lars G. Fritsche, Maria K. Swift, Maryam H. Hilmy, Miao Ling Chee, Milly Tedja, Pieter W. M. Bonnemaijer, Preeti Gupta, Queenie S. Tan, Zheng Li, Eranga N. Vithana, Ravilla D. Ravindran, Soon-Phaik Chee, Yuan Shi, Wenting Liu, Xinyi Su, Xueling Sim, Yang Shen, Ya Xing Wang, Hengtong Li, Yih-Chung Tham, Yik Ying Teo, Tin Aung, Kerrin S. Small, Paul Mitchell, Jost B. Jonas, Tien Yin Wong, Astrid E. Fletcher, Caroline C. W. Klaver, Barbara E. K. Klein, Jie Jin Wang, Sudha K. Iyengar, Christopher J. Hammond, Ching-Yu Cheng

**Affiliations:** 1 Department of Twin Research and Genetic Epidemiology, The School of Life Course Sciences, King’s College London, London, SE1 7EH UK; 2 Singapore Eye Research Institute, Singapore National Eye Center, 168751 Singapore, Singapore; 3 Center for Quantitative Medicine, Duke-NUS Medical School, 169857 Singapore, Singapore; 4 Epidemiology and Biostatistics, Case Western Reserve University, Cleveland, OH 44106 USA; 5 Computational and Systems Biology, Genome Institute of Singapore, 138672 Singapore, Singapore; 6 Department of Epidemiology and Biostatistics, School of Public Health, Tongji Medical College, Huazhong University of Science and Technology, 430030 Wuhan, China; 7 Department of Genetics, Aravind Medical Research Foundation, Madurai, Tamil Nadu 625020 India; 8 Department of Ophthalmology and Visual Sciences, University of Wisconsin School of Medicine and Public Health, Madison, WI 53726 USA; 9 Department of Medicine (Biomedical Genetics), Boston University School of Medicine, Boston, MA 02118 USA; 10 Histology, Department of Pathology, Singapore General Hospital, 169856 Singapore, Singapore; 11 Ophthalmology & Visual Sciences Academic Clinical Program (Eye ACP), Duke-NUS Medical School, 169857 Singapore, Singapore; 12 Centre for Vision Research, Westmead Institute for Medical Research, University of Sydney, Sydney, NSW 2145 Australia; 13 Division of Human Genetics, Genome Institute of Singapore, 138672 Singapore, Singapore; 14 National Eye Institute, National Institutes of Health, Bethesda, MD 20814 USA; 15 Department of Ophthalmology, King’s College London, London, SE5 9RS UK; 16 Department of Epidemiology, Erasmus Medical Centre, 3015 GD Rotterdam, The Netherlands; 17 Department of Ophthalmology, Erasmus Medical Centre, 3015 GD Rotterdam, The Netherlands; 18 Menzies Institute for Medical Research, University of Tasmania, Hobart, TAS 7000 Australia; 19 Department of Ophthalmology, Flinders University, 5042 Adelaide, SA Australia; 20 Center for Statistical Genetics, Department of Biostatistics, University of Michigan, Ann Arbor, MI 48109 USA; 21 K.G. Jebsen Center for Genetic Epidemiology, Department of Public Health, Norwegian University of Science and Technology, 7491 Trondheim, Norway; 22 Department of Anatomical Pathology and Cytology, Singapore General Hospital, 169608 Singapore, Singapore; 23 Institute of Molecular and Cell Biology, 138673 Singapore, Singapore; 24 Aravind Eye Hospital, Madurai, Tamil Nadu 625020 India; 25 Department of Ophthalmology, Yong Loo Lin School of Medicine, National University of Singapore, 117597 Singapore, Singapore; 26 Saw Swee Hock School of Public Health, National University of Singapore, 117549 Singapore, Singapore; 27 Beijing Institute of Ophthalmology, Beijing Ophthalmology and Visual Science Key Lab, Beijing Tongren Eye Center, Beijing Tongren Hospital, Capital Medical University, WC29+WV Beijing, China; 28 Department of Statistics and Applied Probability, National University of Singapore, 119077 Singapore, Singapore; 29 Department of Ophthalmology, Medical Faculty Mannheim of the Ruprecht-Karls-University Heidelberg, Seegartenklinik Heidelberg, 69115 Heidelberg, Germany; 30 Faculty of Epidemiology & Population Health, London School of Hygiene and Tropical Medicine, London, WC1E 7HT UK; 31 Department Ophthalmology, Radboud University Medical Center, Nijmegen, The Netherlands; 32 Institute of Molecular and Clinical Ophthalmology, Basel, Basel, Switzerland; 33 Health Services and Systems Research, Duke-NUS Medical School, 169857 Singapore, Singapore

**Keywords:** Lens diseases, Genome-wide association studies

## Abstract

Nuclear cataract is the most common type of age-related cataract and a leading cause of blindness worldwide. Age-related nuclear cataract is heritable (*h*^*2*^ = 0.48), but little is known about specific genetic factors underlying this condition. Here we report findings from the largest to date multi-ethnic meta-analysis of genome-wide association studies (discovery cohort *N* = 14,151 and replication *N* = 5299) of the International Cataract Genetics Consortium. We confirmed the known genetic association of *CRYAA* (rs7278468, *P* = 2.8 × 10^−16^) with nuclear cataract and identified five new loci associated with this disease: *SOX2-OT* (rs9842371, *P* = 1.7 × 10^−19^), *TMPRSS5* (rs4936279, *P* = 2.5 × 10^−10^), *LINC01412* (rs16823886, *P* = 1.3 × 10^−9^), *GLTSCR1* (rs1005911, *P* = 9.8 × 10^−9^), and *COMMD1* (rs62149908, *P* = 1.2 × 10^−8^). The results suggest a strong link of age-related nuclear cataract with congenital cataract and eye development genes, and the importance of common genetic variants in maintaining crystalline lens integrity in the aging eye.

## Abstract (web summary)

Here, the authors report a multi-ethnic genome wide association meta-analysis of 12 studies from the International Cataract Genetics Consortium. They find six new loci associated with age-related nuclear cataract, in addition to replicating the association at *CRYAA*, and suggest a strong genetic link between age-related nuclear and congenital cataracts.

## Introduction

Age-related cataract is the leading cause of blindness, accounting for more than one-third of blindness worldwide^1,2^. Cataract is an opacification of the lens of the eye, resulting in reduced vision, glare and decreased ability to perform daily activities. Although surgery is often effective in restoring vision, its costs to health-care systems are considerable^3^. The prevalence of cataract and the number of cataract surgeries is projected to rise globally, as the population ages^4,5^, and so will the costs of cataract to society.

The most frequent form of age-related cataract, nuclear cataract (15 year cumulative incidence of 49.6% in individuals aged 65–74 years) affects the lens nucleus^6^. Susceptibility to age-related nuclear cataract (ARNC) was conferred by a mixture of genetic and environmental risk factors: up to half on nuclear cataract variation is due to genetic risk factors^7^, while smoking^8^, obesity^9^ and diet^10^ are potentially modifiable exposures associated with ARNC.

Despite the public health significance of ARNC, relatively little is known about its underlying genetic factors. To date, genome-wide association studies (GWAS) have not been very successful in the identifying common genetic variants for age-related cataract, partly due to the difficulties in objectively phenotyping ARNC. Studies using cataract surgery (either self-reported or based on information from electronic health record) as a proxy for the presence of cataract has been challenging, as the severity of cataract when cataract surgery is done varies greatly among individuals^11,12^. On top of this, there are three major subtypes of age-related cataract (i.e., nuclear, cortical and subcapsular cataract); each of them may have different pathophysiology. To date, the only reported GWAS of ARNC with objective phenotyping via lens photos and detailed cataract grading was done in Asian cohorts, where two genetic loci (*CRYAA*, *KCNAB1*) were found associated with ARNC^13^. The *CRYAA* gene encodes for most abundant structural protein present in the lens and mutations in this gene cause congenital cataracts^14,15^. *KCNAB1* encodes voltage-gated potassium channel, previously linked to ageing bone phenotypes^16^. However, a more recent exome array analysis of ~1500 Europeans failed to find any variants associated at genome-wide significance^17^. Previous analysis of poorly defined (self-report) cataract phenotypes from the UK Biobank (http://www.nealelab.is/uk-biobank, https://www.leelabsg.org/resources) found no common variant associations. A GWAS of retinal detachment in UK Biobank found 20 loci associated with cataract surgery, likely reflecting several age-related cataract subtypes^18^. We are not aware of any other GWAS studies of cataract subtypes, other than for age-related diabetic cataract: a small Taiwanese study found several suggestive loci and a recent larger European-ancestry GWAS identified *CACNA1C* gene at GWAS significance^19,20^.

Given the potential of appropriately powered genetic studies to reveal aetiologies and pathways of ARNC, we aimed to identify additional genomic regions associated with the susceptibility to ARNC via a meta-analysis of GWAS of 12 well-phenotyped studies from the International Cataract Genetics Consortium. We replicated genetic association of *CRYAA* (rs7278468, *P* = 2.8 × 10^−16^) with nuclear cataract and identified six new loci associated with this disease. The results suggest a strong link of ARNC with genes linked to congenital cataract and eye development, as well as and the importance of common genetic variants in maintaining crystalline lens integrity during ageing.

## Results

**Fig. 1:**
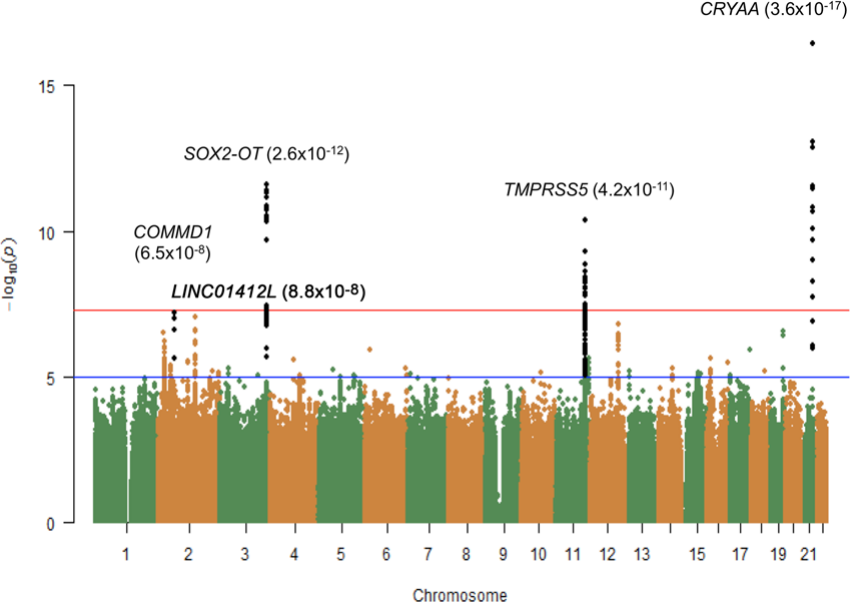
Manhattan plot of the GWAS meta-analysis for age-related nuclear cataract in the combined analysis (*N* = 14,151). The plot shows −log10-transformed *P* values for all SNPs; the upper horizontal line represents the genome-wide significance threshold of *P* < 5.0 × 10^−8^; the lower line indicates a *P* value of 10^−5^. Data of both directly genotyped and imputed SNPs are presented in the Manhattan plot.

The results from the meta-analysis of 8.5 million variants in eight studies (Supplementary Fig. 1 and Supplementary Tables 1–3) followed a polygenic model with no evidence of population structure (meta-analysis genomic inflation factor *λ* = 1.009, Supplementary Table 4 and Supplementary Fig. 2). In the discovery stage we found three loci to be associated at genome-wide significance (Fig. 1) and this number increased to six after the all-data meta-analysis stage (Supplementary Figs. 3–6). As expected for a common age-related trait, the majority of associated variants or variants in LD with those were situated outside of coding regions and we observed suggestive depletion of intronic variants and enrichment in ncRNA and upstream variants (Supplementary Fig. 4).

We confirmed the *CRYAA* genomic region previously found significantly associated with ARNC score at a GWAS-significant level. The strongest evidence for association was found for rs7278468 (*β* = 0.08; *P* = 3.6 × 10^−17^), just upstream of the *CRYAA* gene transcript. However, *KCNAB1* variants that were previously reported in association with ARNC^13^ were rare in Europeans (MAF = 0.03) and were not significantly associated in this meta-analysis (*β* = 0.04; *P* = 0.02 for *KCNAB1* rs55818638). In addition, we identified two novel susceptibility regions that at this stage were significantly associated with ARNC (Table 1 and Supplementary Figs. 3 and 5). Markers located on chromosome 3q26.33, in proximity of the *SOX2* gene and within its regulator, *SOX2-OT*, were significantly associated with the ARNC score (*β* = 0.07; *P*_discovery_ = 2.6 × 10^−12^ for rs9842371). The *SOX2* locus has not previously been associated with nuclear cataract but was associated with cataract surgery in UK Biobank^18^.

**Table 1:** Genome-wide significant associations for age-related nuclear cataract.

						Discovery					Replication		Meta-analysis	
SNP	Chr.	Pos.	Nearest gene	A1	A2	EAF European	EAF Asian	*β* (SE)	*P*_discovery_	*P*_het_	OR (SE)	*P*_replication_	*Z-*score	*P*_combined_
rs61185326	2	20747778	intragenic	T	A	0.04	0.02	0.02 (0.04)	3.0 × 10^−7^	0.09	1.00 (0.21)	0.99	4.60	4.2 × 10^−6^
rs13021828	2	24439276	*ITSN2*	C	G	0.38	0.39	−0.05 (0.01)	6.1 × 10^−7^	0.24	1.07 (0.05)	0.17	−3.54	4.0 × 10^−4^
rs62149908^a^	2	62191878	*COMMD1*	T	C	0.22	0.32	−0.06 (0.04)	6.5 × 10^−8^	0.04	0.89 (0.06)	0.04	−5.70	1.2 × 10^−8^
rs16823886	2	145341259	*LINC01412, ZEB2*	A	G	0.13	0.18	−0.06 (0.01)	8.8 × 10^−8^	0.02	0.86 (0.05)	3.9 × 10^−3^	−6.07	1.3 × 10^−9^
rs9842371	3	181346937	*SOX2*	T	C	0.35	0.54	0.07 (0.01)	2.6 × 10^−12^	0.11	1.31 (0.05)	4.4 × 10^−9^	9.03	1.7 × 10^−19^
rs4936279^a^	11	113566207	*TMPRSS5*	A	C	0.30	0.48	0.06 (0.01)	4.2 × 10^−11^	0.35	1.07 (0.05)	0.18	6.33	2.5 × 10^−10^
rs11067211^a^	12	109988214	*MMAB*	G	A	0.26	0.16	0.06 (0.01)	1.6 × 10^−7^	0.76	1.09 (0.06)	0.14	5.24	1.6 × 10^−7^
rs1005911	19	48206092	*GLTSCR1*	G	T	0.25	0.36	−0.05 (0.01)	2.8 × 10^−7^	0.84	0.87 (0.05)	9.5 × 10^−3^	−5.73	9.8 × 10^−9^
rs7278468^a^	21	44588757	*CRYAA*	G	T	0.69	0.37	0.08 (0.01)	3.6 × 10^−17^	0.27	1.13 (0.07)	0.06	8.18	2.8 × 10^−16^

This table summarises the SNPs that were associated at genome-wide significance level (*P* < 5 × 10^−8^) with age-related nuclear cataract in the combined analysis (discovery phase) and/or after the replication phase. *SNP* single-nucleotide polymorphism, *chr.* chromosome, *pos* position (NCBI build 37), *A1* reference allele 1, *A2* the other allele, *EAF* effect allele frequency, *Beta* effect size on standardised nuclear cataract scores based on the effect allele in all discovery cohorts meta-analysis, *SE* standard errors of the effect size, *P*_het_, *P* value for heterogeneity, *OR* odds ratio estimated from the case–control collections in the replication phase, *Z* *Z-*score derived from the overall meta-analysis combining the discovery and replication phases. ^a^These variants were not available in the INDEYE(S) study due to probe design issues and the following variants in high linkage disequilibrium with the lead SNP were genotyped instead: rs55785307 (*COMMD1*, *R*^2^ = 0.84; *D*′  = 0.99), rs11601037 (*TMPRSS5*, *R*^2^ = 0.90; *D*′ = 1.0), rs7486178 (*MMAB*, *R*^2^ = 0.83; *D*′ = 0.99) and rs870137 (*CRYAA*, *R*^2^ = 0.48; *D*′ = 0.98).

A second novel susceptibility genetic locus significantly associated with ARNC score was located on chromosome 11.q23.2 and overlapped with the genomic sequence of the *TMPRSS5* gene (*β* = 0.06; *P*_discovery_ = 4.2 × 10^−11^ for rs4936279). Furthermore, a third locus, overlapping with the *COMMD1* gene-coding region, also approached genome-wide significance in this meta-analysis (*β* = −0.06; *P*_discovery_ = 6.5 × 10^−8^ for rs62149908). Among the genes that were associated at suggestive, but not GWAS-significant levels overall, ancestry-specific significant associations were observed at chromosome 13q12.11 in Asians (*β* = 0.07; *P*_Asians_ = 2.7 × 10^−8^ for rs4769087) within the *GJA3* genomic sequence, and on chromosome 11q23.1 in Europeans upstream of *CRYAB* (*β* = 0.07; *P*_Europeans_ = 2.5 × 10^−5^ for rs10789852).

Genome-wide associated SNPs showing suggestive association (*P* < 10^−6^) in the discovery phase were taken forward to the replication stage of this study (Table 1). Despite the smaller sample size for replication, four out of nine markers tested showed nominal replication (*P* < 0.05, Supplementary Fig. 7). Another three of the SNPs failed to achieve significance, but the association in the replication meta-analysis was in the same direction as that in the discovery phase (Table 1). Notably association was replicated for markers in the *SOX2* locus (OR = 1.31; *P* = 4.4 × 10^−9^ for rs9842371), but the replication results were not statistically significant for the markers in the *TMPRSS5* locus, nor in the previously established *CRYAA* locus (OR = 1.13; *P* = 5.6 × 10^−2^ for rs7278468). Nevertheless, we observed that the direction of allele’s effects was the same between the discovery stage and replication stage all SNPs (i.e., the allele associated with higher ARNC score in the discovery stage also had a odds ratio of >1 for ARNC in the replication stage), except *ITSN2* rs13021828.

An all-inclusive meta-analysis of all leading SNPs of regions associated at or close to GWAS-significance levels using both the discovery and replication loci was performed (Table 1 and Supplementary Fig. 4). In addition to the loci of *SOX2*/*SOX2-OT* (*Z* = 9.03; *P* = 1.7 × 10^−19^ for rs9842371), *CRYAA*, (*Z* = 8.18; *P* = 2.8 × 10^−16^ for rs7278468) and *TMPRSS5* (*Z* = 6.33; *P* = 2.5 × 10^−10^ for rs4936279), novel genome-wide significant associations were found for rs16823886 upstream of the *ZEB2* gene (*Z* = −6.07; *P* = 1.3 × 10^−9^), rs62149908 (*Z* = −5.70; *P* = 1.2 × 10^−8^), within the Copper Metabolism Domain Containing 1 (*COMMD1*) gene; for rs1005911 within the *GLTSCR1* gene (*Z* = −5.73; *P* = 9.8 × 10^−9^). At those loci, the following genes are expressed in lens (Supplementary Table 6): *ZEB2*, *GLTSCR1*, *NAPA*, but the eQTL and regulatory sequence analysis (Supplementary Fig. 6, Supplementary Tables 5, Supplementary Data 1) did not provide conclusive evidence on how those genes may exert their effects on ARNC formation. The eQTL analysis (Supplementary Data 1), however, found a strong association between the following SNPs and transcript levels: rs7278468 and the *CRYAA* (*P* = 1.3 × 10^−7^, liver tissue); rs11067211 and *MMAB* (*P* = 5.3 × 10^−8^, brain); rs61185326 and *RHOB* (*P* = 3.0 × 10^−7^, muscle); and rs10789852 and *CRYAB* (*P* = 7.6 × 10^−22^; fat). It is possible that similar effects are present for other genes, but at tissues and developmental stages that are not captured in the available GTEx or TwinsUK tissues.

The common variants associated at GWAS-levels with ARNC in our discovery stage analysis explained ~3% of heritability. A conditional analysis of SNPs identified from discovery phase loci (Supplementary Table 7) and a gene-based test (Supplementary Table 8) was performed on the results of the discovery stage meta-analysis, but they did not yield any additional association beyond those already reported above. Pathway analysis were a few pathways associated with ARNC (Supplementary Table 9), with the strongest enrichment observed for cholesterol biosynthesis (*P*_permuted_ = 0.01), whose importance in cataract is not clearly known.

An LDscore systematic analysis of genetic correlations suggested that the ARNC genetic risk was correlated with the following eye-related traits measured in UK Biobank: cataract (0.48), diabetes-related eye diseases (0.27) and glaucoma (0.20). In addition, there was correlation with the genetic risks of (Supplementary Fig. 8) hip (0.34) and waist (0.30) circumference, different classes of circulating lipids (median = 0.26) and age at menarche (**−**0.12). However, none of the correlations survived correction for multiple testing. Similarly, the Open Targets SNP and gene co-localisation results point to sharing of signals with astigmatism-related traits (*CRYAA*, *SOX2* and *GLTSCR1* loci), cardio-metabolic traits, anthropometric and blood cell traits (Supplementary Fig. 9). Of note, there was also co-localisation with smoking-related GWAS signals at the *ZEB2* and *ITSN2* loci (Supplementary Fig. 9).

**Fig. 2:**
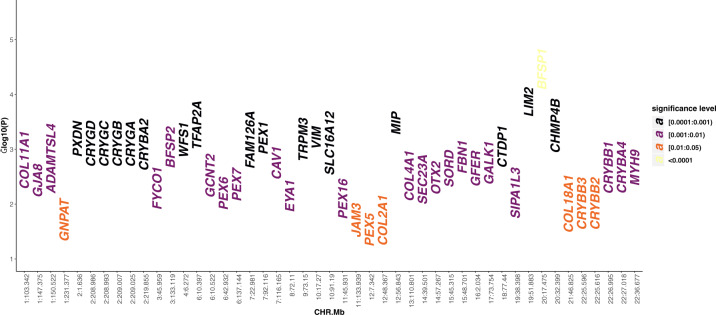
Common variants in congenital cataract genes. This Manhattan plot shows the association results for the congenital cataract genes. The −log10(*P* value) of the most strongly associated variant per gene is plotted against the gene location (in chromosome followed by mega base format: CHR.Mb). The colour code represents the strength of association in terms of *P* value.

Multiple variants in proximity to 47 genes linked to congenital cataract were nominally associated with ARNC in our analysis (Fig. 2 and Supplementary Data 2), but only 5 survived correction for multiple testing (*α* = 5 × 10^−4^) in *BFSP1* (*β* = 0.08; *P* = 3.5 × 10^−5^), *LIM2* (*β* = 0.04; *P* = 1.4 × 10^−4^), *MIP* (*β* = 0.02; *P* = 3.4 × 10^−4^*), TFAP2A* (*β* = 0.05; *P* = 3.7 × 10^−4^) and *CHMP4B* (*β* = 0.08; *P* = 3.8 × 10^−4^).

## Discussion

Here we report the results of a GWAS on nuclear cataract, conducted on 14,151 participants with detailed ARNC severity phenotypes and replicated in 5299 samples. Apart from confirming association at the *CRYAA*, we increased the number of known associations by reporting five additional ARNC genetic loci.

The SOX2 Overlapping Transcript (*SOX2-OT*) encodes for a highly conserved long noncoding RNA, which overlaps and regulates *SOX2* expression. SOX2 is a single exon transcription factor, previously associated with anophthalmia^21^ and coloboma^22^. Sox2 is involved in crystallin regulation in murine^23^ and avian models^24^ and in humans, and *SOX2* mutations cause microphthalmia and cataract^25–27^.

ZEB2 is a still uncharacterised member of the Zinc Finger E-Box Binding Homeobox family. However a structurally similar member of the same family, ZEB1 is associated to Fuch’s^28^ and posterior^29^ corneal dystrophy, while COMMD1 is involved in copper homoeostasis^30^ and metabolism and in Wilson’s disease^31^. Mutations in the *UBE3* gene are known causes of the Kaufman oculocerebrofacial syndrome^32^, a severe malformation in the newborn with numerous ocular manifestations.

We observe association for genetic variants near the *GJA3* locus, as previously reported^33,34^; however, this association was ethnicity-specific and could not be replicated in Europeans or in the smaller cohort of nuclear cataract case–control replication panel. This gene encoding for a gap-junction connexin (Connexin-46, CXA46) can induce cataract in animal models^35^ and some of its mutations cause congenital cataracts in humans^36,37^. Given the evidence for association and its biological properties, variants at the *GJA3* locus need to be better characterised in future studies.

Variants in proximity to the *CRYAA* and *CRYAB* gene, encoding for the two forms of α-crystallin, were associated with ARNC. The α-crystallins contribute to the clarity and refractive properties of the lens, may prevent protein damage and protect against oxidative stress^33,34,38^. The common variants that we identified appear to affect transcription and expression of these genes, as suggested by previous studies where both proteins were down-regulated in lenses with ARNC^13,39,40^.

Most of the genes located nearest to our association signals have functional properties that suggest an involvement in eye morphogenesis in general and crystallin expression and regulation. This together with the signals from the genes linked to congenital cataract point to overlap in mechanisms between the congenital and late-onset forms. In that respect, the genetic architecture of ARNC likely does not differ from other common complex conditions where deleterious coding variants cause congenital forms while common variants regulating gene expression are associated with increased risk of developing age-related forms. Given that smoking is an established risk factor for ARNC, it is also interesting that two of the loci co-localised with signals from GWAS of smoking. What is intriguing and would merit further research is the suggested systemic involvement in the disease. Both the Open Targets colocalization analysis and LDscore results suggest genetic sharing with metabolic syndrome components^41^, age at menarche and other hormonal factors^42^ in the pathogenesis of cataract. Systemic risk factors are known to influence other age-related cataract forms, such as cortical and diabetic cataracts, and when well-phenotyped and well-powered GWAS for these phenotypes become available, it will be interesting to see if there is any genetic overlap between those and loci identified here.

This work has several strengths, such as the use of the largest sample to date for genetic analysis of ARNC and, more importantly in the discovery phase, of precisely and quantitatively phenotyped cohorts. It also provides evidence of genetic mechanisms shared between congenital and age-related cataract and shows the importance of common genetic variants in maintaining crystalline lens integrity in the aging eye.

This study also has some limitations. The GWAS used in this study employed different grading systems, and despite phenotypic standardisation before the analyses, some residual heterogeneity between the studies may not be fully excluded. This study also sought to maximise the discovery power at the expense of increasing heterogeneity. We believe that replication was constrained by the power in the replication sample: a combined panel of 2807 cases and 2492 controls would afford sufficient (≥0.7) power only to the most common and strongest genetic effects (Fig. S5), which in our case are only encountered in the *SOX2* locus.

However, our conservative approach at dealing with the ethnic heterogeneity may have uneven power across the regions where there are significant differences the LD structure between the two main ancestral groups (European and Asian), or whenever there are significant differences in the minor allele frequency at certain loci. These circumstances, however, would have not affected the specificity of our findings.

Notwithstanding imperfections arising from sample and phenotypic availability, this study has doubled the number of loci positively associated with cataract and improved the proportion of phenotypic variance explained by them. The remaining heritability gap will be reduced by future with more powered, well-phenotyped studies and cohorts to further confirm association of known loci with ARNC and improve our understanding of the genetic architecture of this age-related cataract type.

## Methods

Meta-analyses of summary statistics from GWAS were performed in four cohorts of European (*N* = 7352) and four of Asian (*N* = 6799) ancestry. Genetic variants associated with ARNC at GWAS (*P* < 5 × 10^−8^) or suggestive levels of statistical significance (*P* < 1 × 10^−6^) were carried forward for replication in the four additional cohorts.

### Subjects and phenotyping

The following population-based cohort studies were included in the meta-analyses: Age-related Eye Diseases Study (AREDS), Blue Mountains Eye Study (BMES)^43^, Rotterdam Study I, Rotterdam Study phase III (RSI-III)^44^ and TwinsUK^45^ all of European ancestry, as well as Singapore Malay Eye Study (SiMES)^46^, Singapore Indian Eye Study (SINDI)^47^ and two separate subsets of the Singapore Chinese Eye Study (SCES)^47^. Detailed demographic information and phenotyping methods are shown in the Supplementary Notes and Supplementary Tables 1 and 2. All studies were conducted with the approval of their local Research Ethics Committees, and written informed consent was obtained from all participants, in accordance with the Declaration of Helsinki.

All participants underwent detailed eye examination, including lens photography after pupil dilation for quantitative assessment of cataract severity in the discovery phase. Nuclear cataract was graded using standard grading systems from lens photographs (Supplementary Tables 1 and 2 and Supplementary Note: Grading systems) and, when scores for both eyes were available, the higher of the two scores was used in the analyses. Individuals who had undergone cataract surgery in both eyes were excluded.

In the replication phase, a dichotomous nuclear cataract status (presence or absence) was used as phenotypic outcome for the association models. This categorical binary trait was used as only semi-quantitative grading was available from these study populations, either from slit-lamp grading by clinician or from lens photography. In the replication phase we used two population-based cohorts of Asian ancestry, the Beijing Eye Study (BES)^48^ and India Eye Study-South India (INDEYE(S)^49^ as well as two European cohorts, the population-based (Beaver Dam Eye Study or BDES^50^) and a clinic-based case–control study (South London Case Control Study or SLCCS). The definition of cataract cases is shown in Supplementary Tables 1 and 2; the criteria included AREDS grade 3 or more for BES^48^, LOCS III grade 4 or higher for INDEYE(S)^49^, Wisconsin grade 3 or higher for BDES^50^ and LOCS III grade 3 or higher for SLCCS. Controls were individuals with no significant nuclear opacity at the time of recruitment and no prior history of cataract surgery.

### Genotyping and imputation

Different platforms were used for the genotyping of each cohort (Supplementary Table 3). All GWAS datasets were imputed against the 1000 Genomes Phase 1, with either IMPUTE2 (ref. ^51^) or Minimac^52^.

### Statistical analysis

In the discovery phase, we included only cohorts where ARNC phenotyping was conducted according to an objective, standardised grading system of nuclear cataract severity. The details of each cohort and ARNC phenotyping can be found in Supplementary Tables 1–3, Supplementary Fig. 1 and Supplementary Notes. The distribution of quantitative ARNC scores was normalised whenever needed, and subsequently standardised within each cohort (mean 0 and standard deviation 1). The distribution of the transformed phenotypes is shown in Supplementary Fig. 1. For the replication, we used four cohorts of nuclear cataract patients and cataract-free controls (Supplementary Tables 2 and 3), not included in the quantitative, discovery phase (due to unavailability of genome-wide genotyping or quantitative nuclear cataract information).

Each cohort was ancestrally homogeneous: ethnic outliers were identified through Principal Component Analysis clustering and excluded from subsequent analyses. Genome-wide association analyses were performed in each cohort separately by building additive linear regression models, with the standardised ARNC score as the dependent variables and the number of alleles at each genetic locus as the explanatory variables, adjusting for age, sex and, when appropriate, principal components. In TwinsUK, linear mixed models with a kinship matrix as a random effect term (GEMMA)^53^ were used to account for non-independence of observations due to familial relationships.

Fixed-effect inverse-variance meta-analyses using METAL^54^ were performed on the GWAS summary statistics provided by each study for all variants with MAF >1%, genotyping call rate >0.97 and imputation quality >0.3 (the ‘*RSQ*’ parameter in MACH^55^ or ‘*info*’ for IMPUTE^51^) that were present in at least three of the European or at least three of the Asian cohorts. Additionally, variants showing high heterogeneity (*I*^2^ > 0.75) were excluded.

Gene-based analyses were performed using GATES^56^ and gene set enrichment analysis using PASCAL^57^. The proportion of genetic variance explained by associated SNPs was calculated using individual-level data using GCTA^58^. Shared heritability between ARNC and other traits, for which GWAS results were available through the LDscore Hub website, was calculated using linkage disequilibrium score regression^59^, taking Europeans as a reference.

Genome-wide associated SNPs showing suggestive association (*P* < 10^−6^) in the discovery phase were taken forward to the replication stage of this study. We performed logistic regressions within each replication cohort, followed by an inverse-variance meta-analysis. Finally, SNPs that were identified through discovery and were genotyped in replication cohorts were meta-analysed together through a sample size-weighted *P* value analysis using METAL^54^.

### Gene expression in publicly available databases

Gene expression data in human and mouse lens were obtained using publicly available databases: iSyte^60^, Ocular tissue database and the Mouse Genome informatics (MGI) gene expression database. Expression patterns were examined not only for the gene closest to the most strongly associated variant in each associated region, but also for all other genes in in the same LD block with them.

### eQTL analysis

Lens tissue eQTLs are not currently available, but as eQTL effects are often shared between tissues^61,62^, we assessed whether SNPs associated with nuclear cataract (*P* < 1 × 10^−5^) regulate gene expression of adjacent genes (i.e. have eQTL effects) by searching publicly available data (GTEx)^63^ and the available literature^64^.

### Regulatory elements

The most significantly associated variant at each locus was annotated for regulatory functions (enhancer histone modification signals, DNase I hypersensitivity, binding of transcription factors or effects on regulatory motifs), using HaploReg^65^ and ENCODE data track in the UCSC genome browser.

### Additional annotation and data integration

Additional annotation and data integration were performed using FUMA (https://fuma.ctglab.nl, SNP2GENE and GENE2FUNCTION) and Open Targets Genetics (https://genetics.opentargets.org/, sentinel-variant PheWAS and candidate gene co-localisation).

### Congenital cataract genes

Given the significant associations of markers within or in the proximity of congenital cataract genes such as *GJA3* and *CRYAA*, we enquired whether other common variants within genomic regions hosting additional known congenital cataract loci^66,67^ were associated with ARNC. We explored association for a list of genes linked to congenital cataract by an extensive literature search and by using following databases: Online Mendelian Inheritance in Man (OMIM), Cataract Map (Cat-Map) and Clinical Variants (ClinVar). Each database was queried for variants within a 100 kb window and within the same LD block as the strongest associated SNP.

### Web resources

http://genome.ucsc.edu/

http://ldsc.broadinstitute.org/

https://genome.uiowa.edu/otdb/

http://Supplemental.informatics.jax.org/

http://Supplemental.gtexportal.org/home/

http://omim.org/

https://cat-map.wustl.edu/

https://Supplemental.ncbi.nlm.nih.gov/clinvar/

https://fuma.ctglab.nl

https://genetics.opentargets.org/

### Reporting summary

Further information on research design is available in the Nature Research Reporting Summary linked to this article.

## Supplementary information

Supplementary Information

Description of Additional Supplementary Files

Supplementary Data 1

Supplementary Data 2

Supplementary Data 3

Reporting Summary

## Data Availability

The GWAS summary statistics are available in Supplementary Data 3. Individual-level data can be requested by contacting the participating studies.
