# Supplementary material for: Common variants in SOX-2 and congenital cataract genes contribute to age-related nuclear cataract
Source: Commun Biol. 2020 Dec 11;3:755. doi: 10.1038/s42003-020-01421-2 (PMC7733496; doi:10.1038/s42003-020-01421-2)
Supplement: Supplementary file 1 — Supplementary Information [file 42003_2020_1421_MOESM1_ESM.pdf]

Supplementary Information

Common variants in *SOX-2* and congenital cataract genes contribute to age-related nuclear cataract

Ekaterina Yonova-Doing,[1*] Wanting Zhao,[2,3*] Robert P. Igo Jr,[4*] Chaolong Wang,[5,6*] Periasamy Sundaresan,[7] Kristine E. Lee,[8] Gyungah Jun,[9,10] Alexessander Couto Alves,[1] Xiaoran Chai,[2] Anita S. Chan,[2,11] Mei Chin Lee,[2,11] Allan Fong,[2] Ava G. Tan,[12] Chiea Chuen Khor,[2,13] Emily Chew,[14] Pirro G. Hysi,[1,28] Fan Qiao,[2,3] Jacqueline Chua,[2,11] Jaeyoon Chung,[10] Jiemin Liao,[2] Johanna M. Colijn,[15,16] Kathryn Burdon,[17,18] Lars G. Fritsche,[19,20] Maria K. Swift,[8] Maryam H. Hilmy,[21] Miao Ling Chee,[2] Milly Tedja,[15,16] Pieter Bonnemaijer,[15,16] Preeti Gupta,[2] Queenie S. Tan,[22] Zheng Li,[13] Eranga N. Vithana,[2,11] Ravilla D. Ravindran,[23] Soon-Phaik Chee,[2,11,24] Yuan Shi,[2] Wenting Liu,[13] Xinyi Su,[11] Xueling Sim,[25] Yang Shen,[5] Ya Xing Wang,[26] Hengtong Li,[2] Yih-Chung Tham,[2] Yik Ying Teo,[25,27] Tin Aung,[2,11,24] Kerrin S. Small,[1] Paul Mitchell,[12] Jost B. Jonas,[26,29] Tien Yin Wong,[2,11,24] Astrid E. Fletcher,[29] Caroline C. Klaver,[15,16] Barbara E. K. Klein,[8] Jie Jin Wang,[12,31] Sudha K. Iyengar,[4] Christopher J. Hammond,[1,28,**] Ching-Yu Cheng[2,3,11,24,**]

Prof Christopher Hammond
Email: chris.hammond@kcl.ac.uk

Prof Ching-Yu Cheng
Email: chingyu.cheng@duke-nus.edu.sg

This file includes:

Supplemental Figures 1 to 9
Supplemental Tables 1 to 11
Supplementary Note
References
Acknowledgments

Supplementary Figure 1. Distribution plots of standardized residuals for the Discovery phase cohorts.

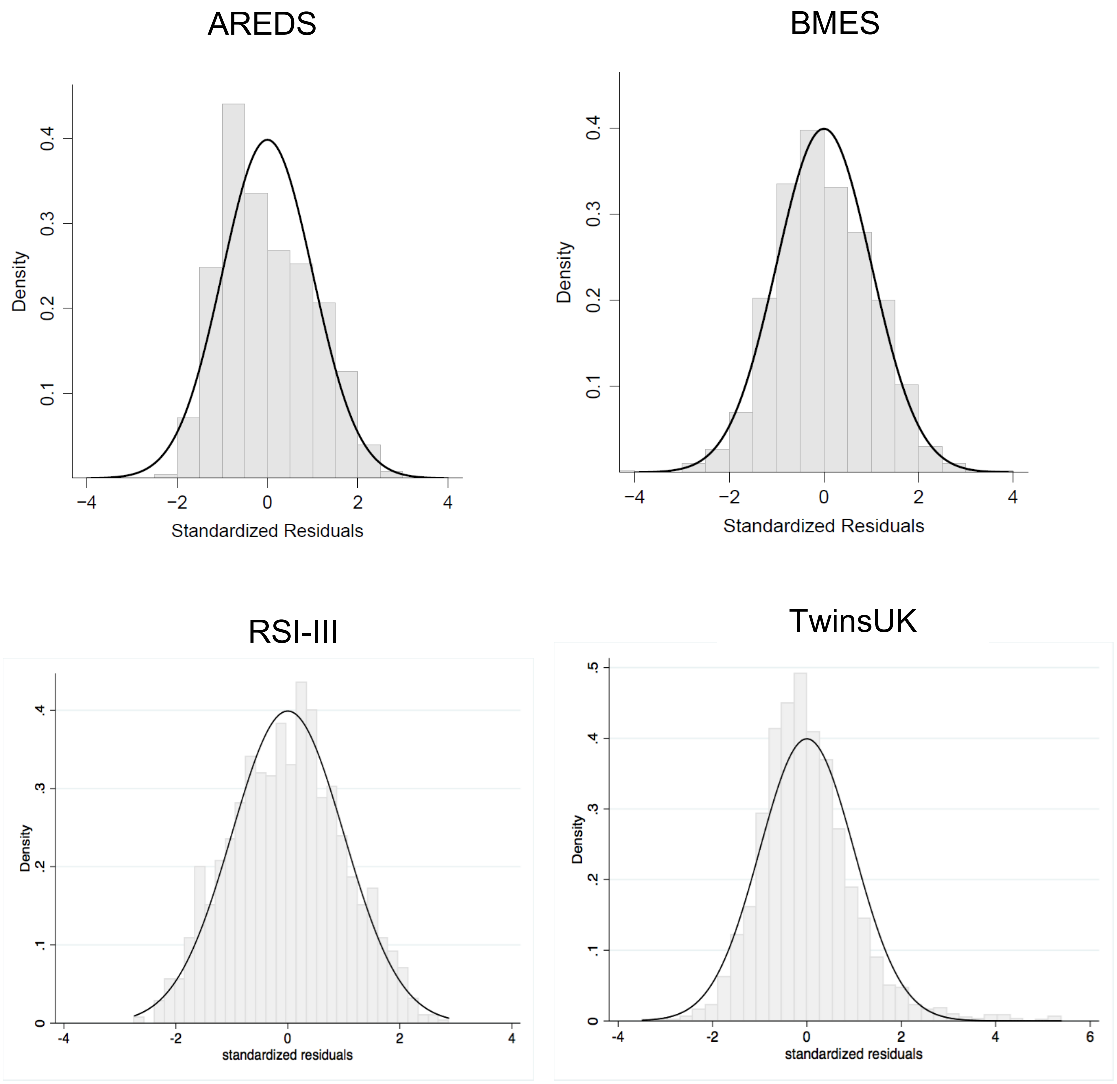

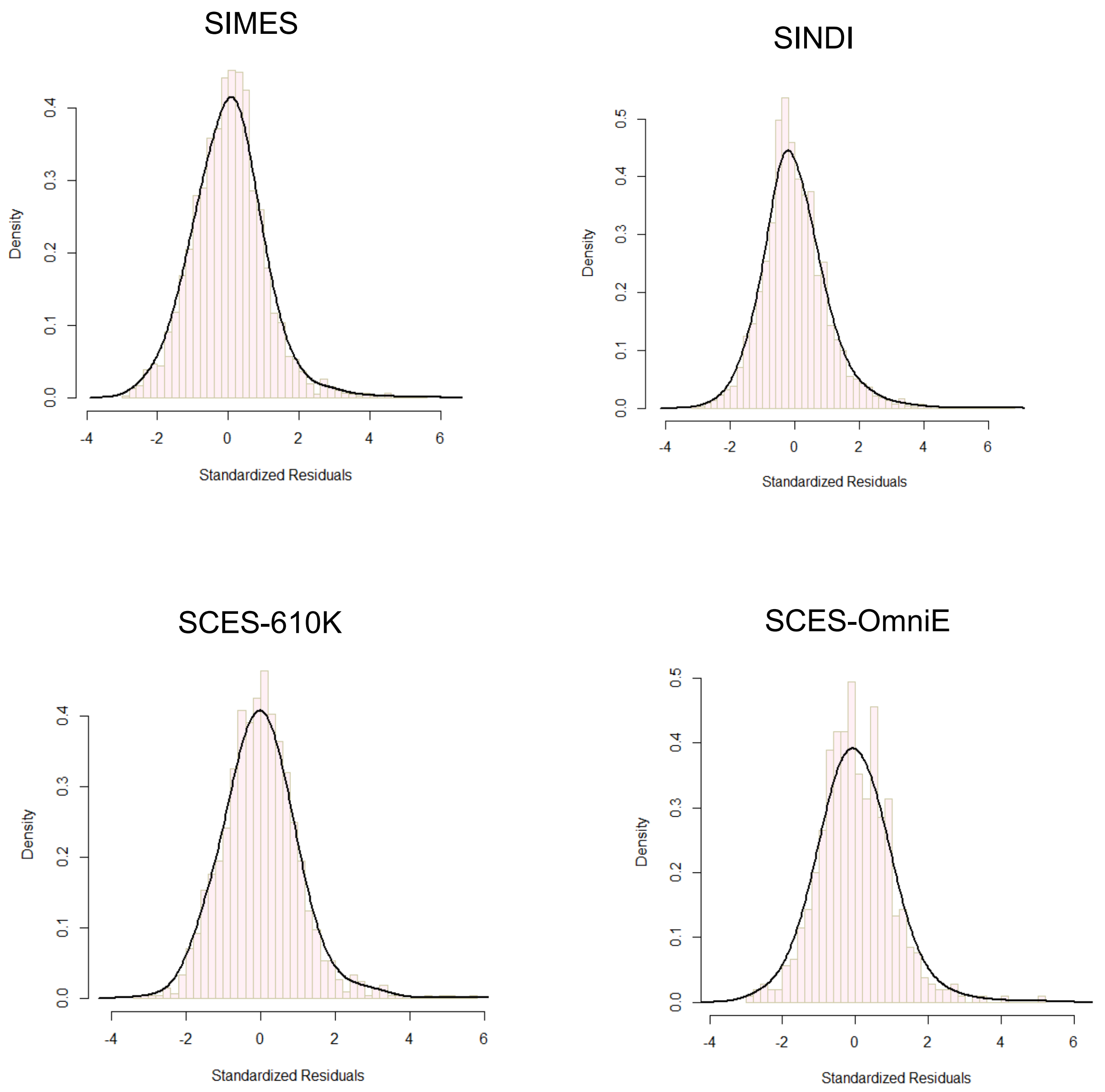

This figure shows the distribution of the standardised residuals resulting from the regression analysis of age-related nuclear cataract in the participating discovery cohorts. European ancestry cohorts are shaded in grey and the Asian ancestry cohorts are shaded in pink.

Supplementary Figure 2. Quantile-Quantile (QQ) plot for association between all SNPs analyzed and age-related nuclear cataract in this meta-analysis for *A*) European ancestry cohorts, *B*) Asian ancestry cohorts, and *C*) Combined analysis.

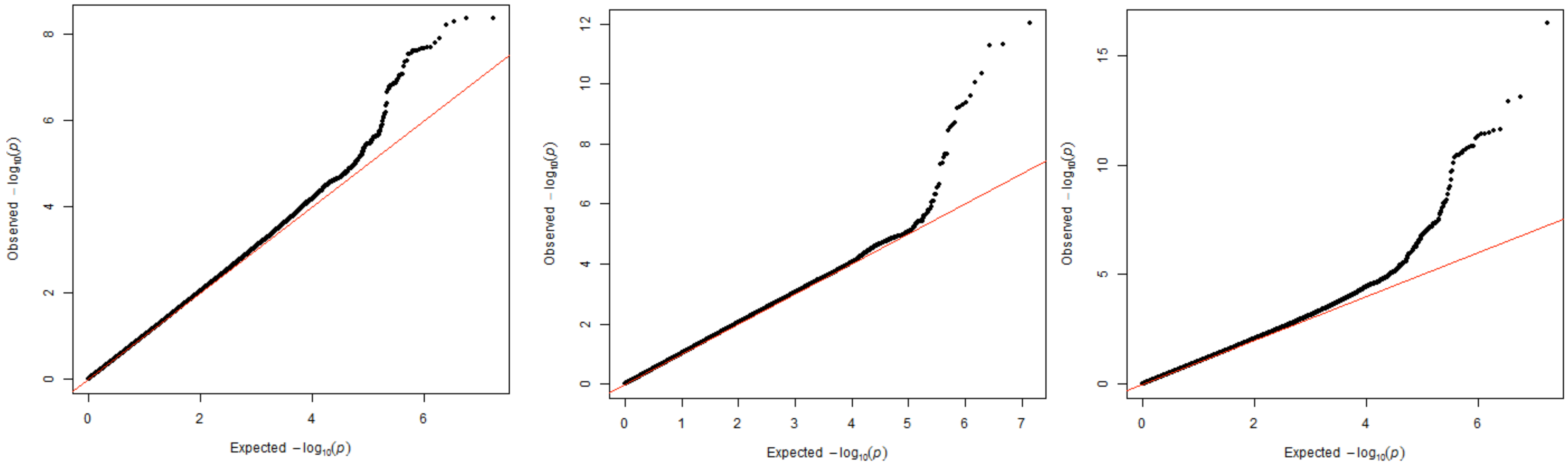

Each dot represents an observed statistic (defined as log10 P) versus the corresponding expected statistic. The red line corresponds to the null distribution. The inflation factors (λ) of the test statistics in the discovery meta-analysis phases were as follows: $\lambda_{Europeans}$=1.02, λ_{Asians}=1.03, and $\lambda_{overall}$=1.04.

Supplementary Figure 3. Summary results from FUMA

A) Functional consequences of SNPs on genes

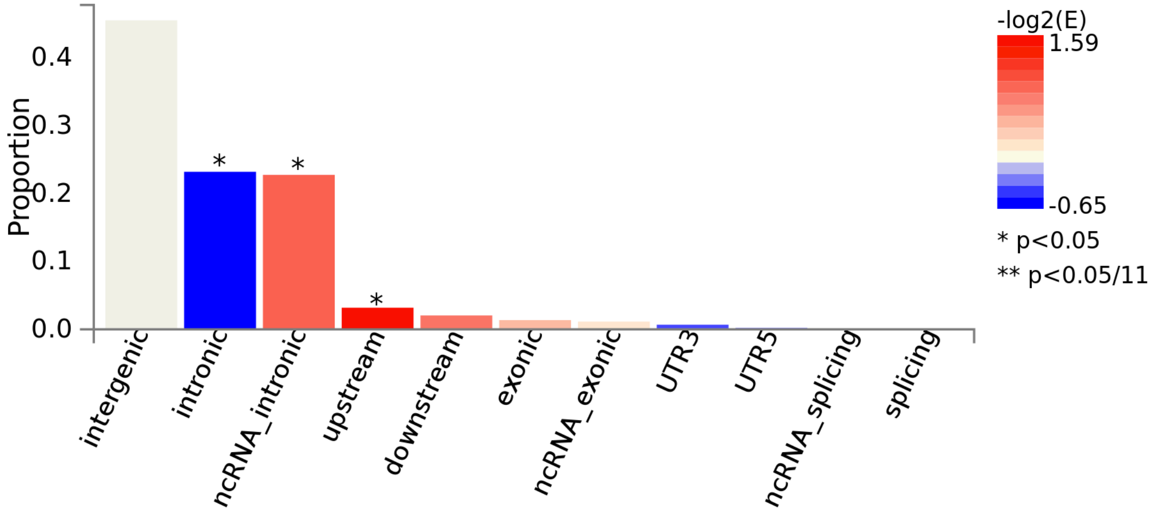

B) Summary per genomic risk locus

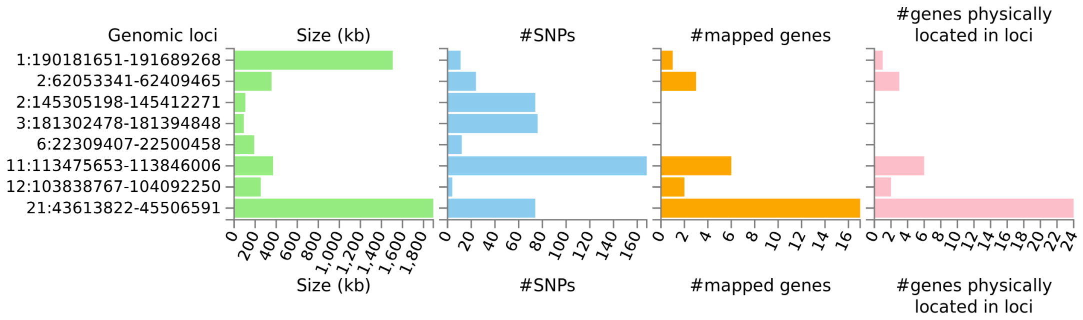

Supplementary Figure 4. Regional association and recombination rate plots for the nine loci which were selected for replication as follows: A) *CRYAA,* B) *SOX2-OT*, C) *TMPRSS5*, D) *COMMD1* E) *LINC01412* F) *MMAB* G) *GLTSCR1* H) rs61185326 locus, and I) *ITSN2.*

A

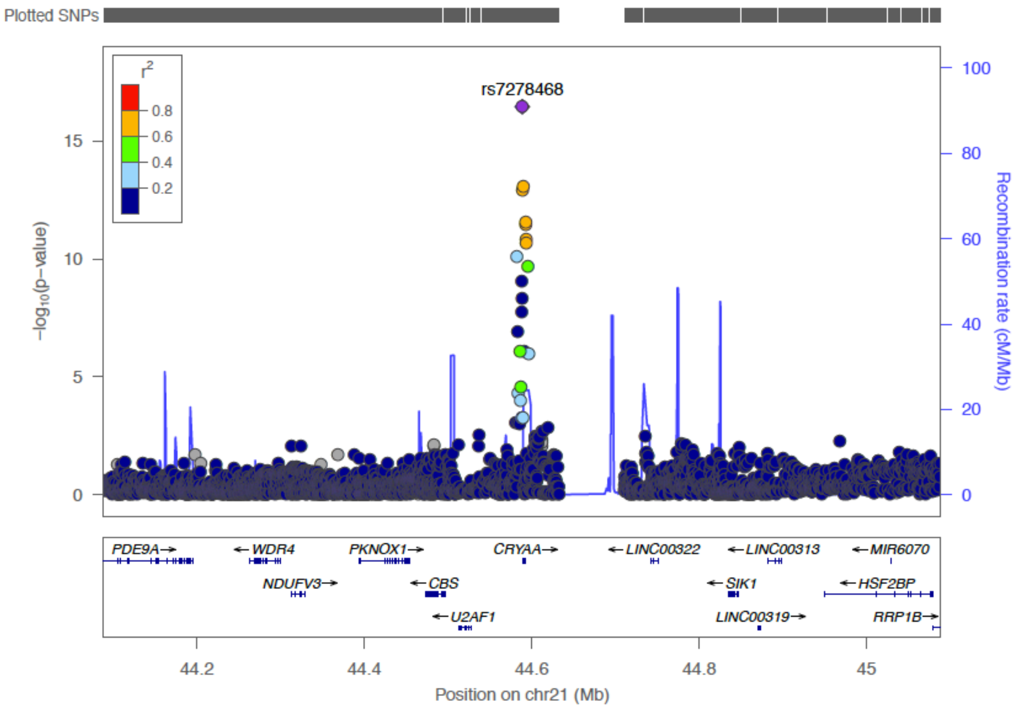

B

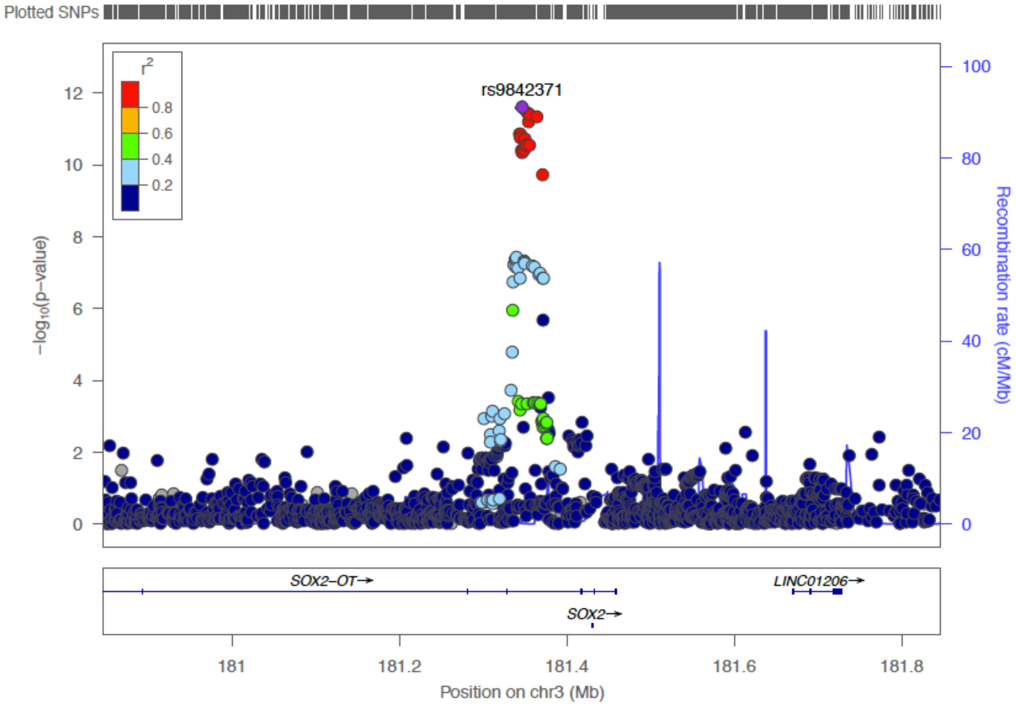

C

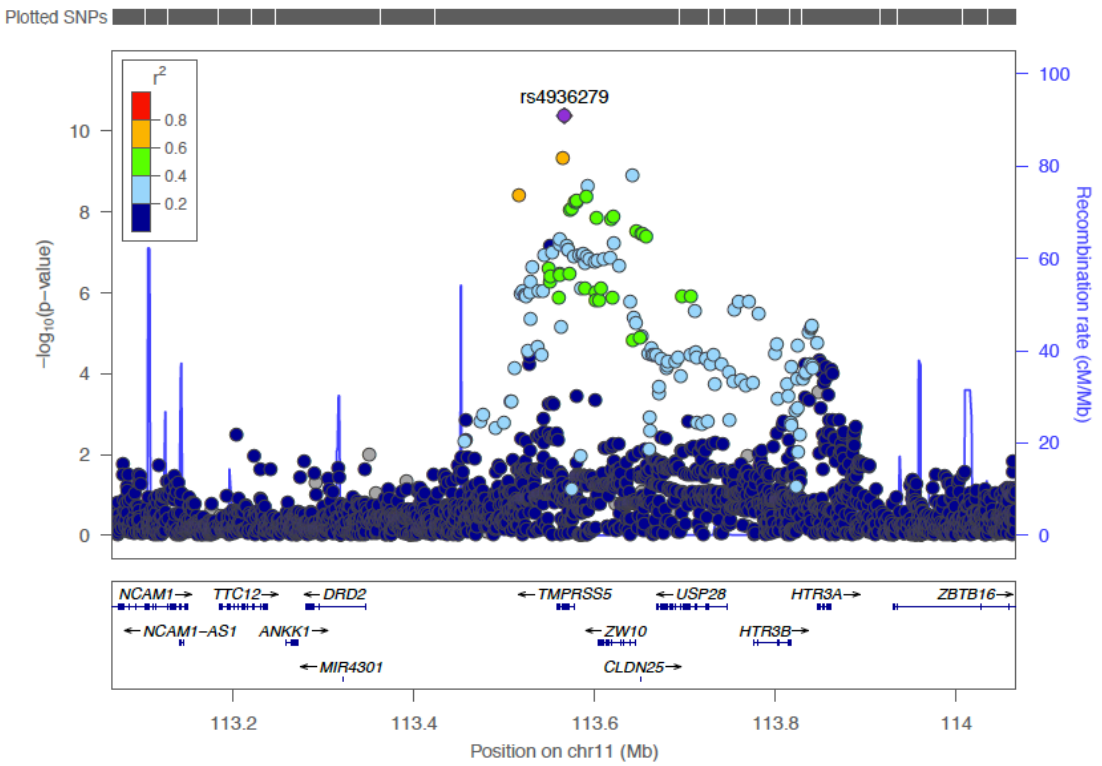
Plotted SNPs
rs4936279
r²
0.8
0.6
0.4
0.2
−log10(p−value)
Recombination rate (cM/Mb)
NCAM1→
TTC12→
←DRD2
←TMPRSS5
←USP28
HTR3A→
ZBTB16→
←NCAM1−AS1
ANKK1→
←ZW10
HTR3B→
←MIR4301
CLDN25→
113.2
113.4
113.6
113.8
114
Position on chr11 (Mb)

D

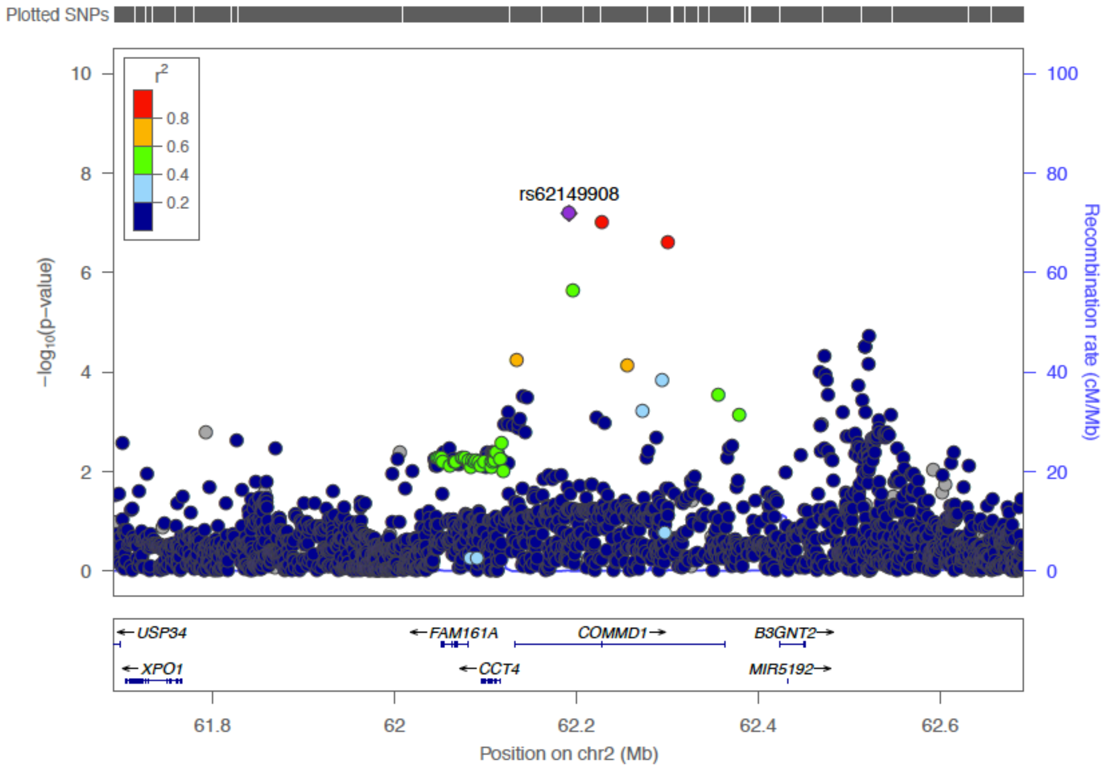
Plotted SNPs
rs62149908
r²
0.8
0.6
0.4
0.2
−log10(p−value)
Recombination rate (cM/Mb)
←USP34
←FAM161A
COMMD1→
B3GNT2→
←XPO1
←CCT4
MIR5192→
61.8
62
62.2
62.4
62.6
Position on chr2 (Mb)

E

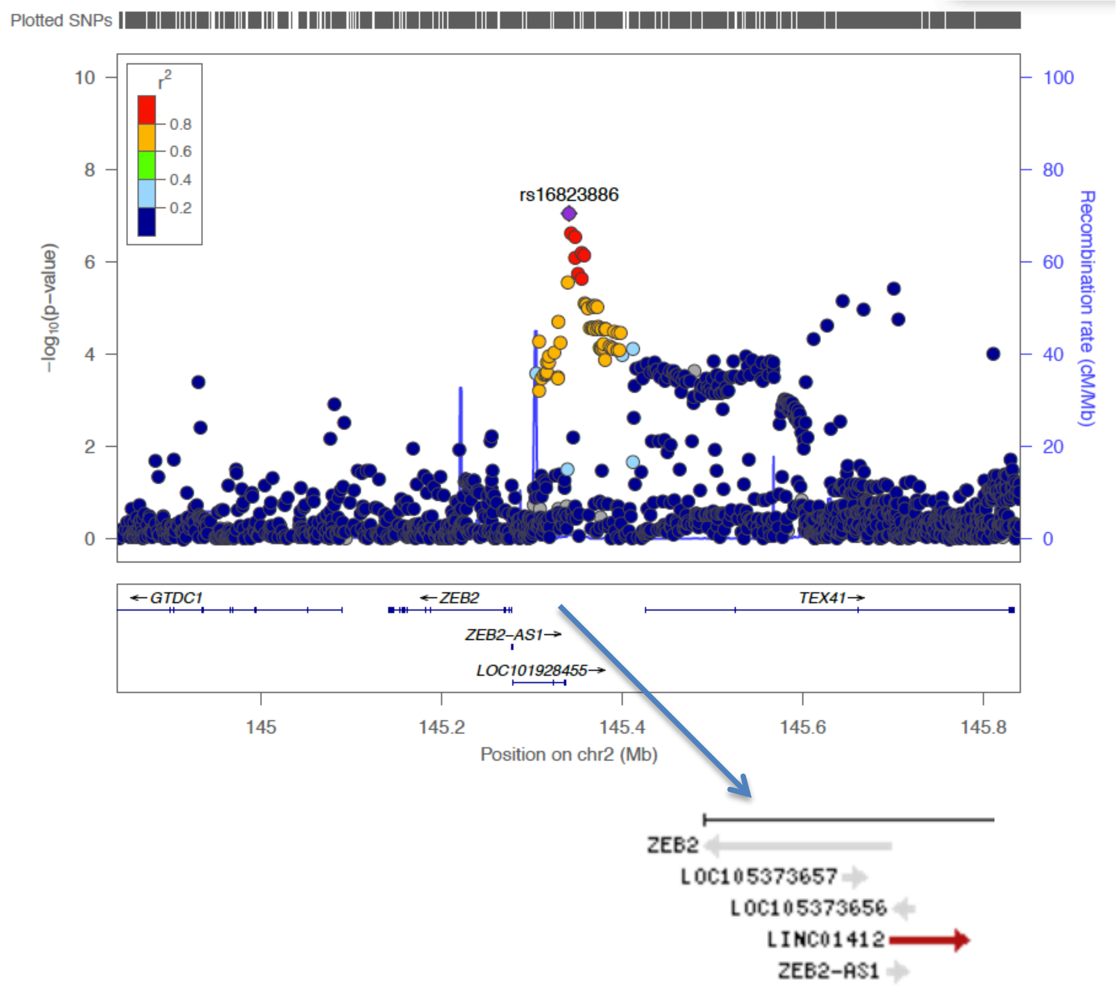
Plotted SNPs
r2
0.8
0.6
0.4
0.2
rs16823886
−log10(p−value)
Recombination rate (cM/Mb)
GTDC1
ZEB2
TEX41
ZEB2−AS1
LOC101928455
145
145.2
145.4
145.6
145.8
Position on chr2 (Mb)
ZEB2
LOC105373657
LOC105373656
LINC01412
ZEB2-AS1

F

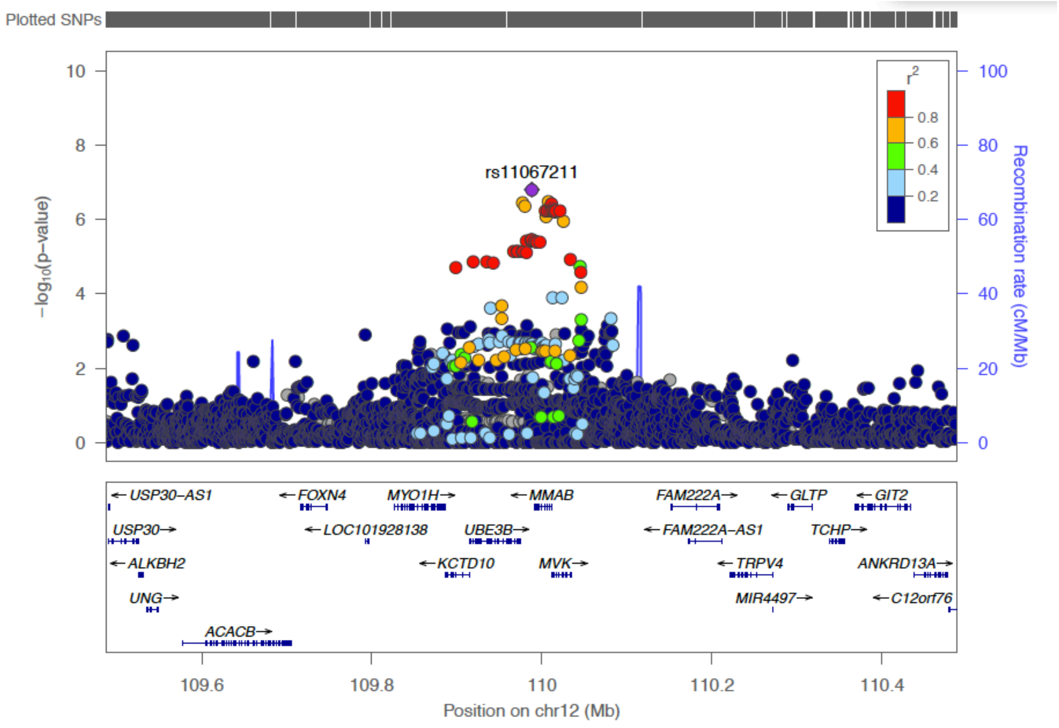
Plotted SNPs
r2
0.8
0.6
0.4
0.2
rs11067211
−log10(p−value)
Recombination rate (cM/Mb)
USP30−AS1
FOXN4
MYO1H
MMAB
FAM222A
GLTP
GIT2
USP30
LOC101928138
UBE3B
FAM222A−AS1
TCHP
ALKBH2
KCTD10
MVK
TRPV4
ANKRD13A
UNG
MIR4497
C12orf76
ACACB
109.6
109.8
110
110.2
110.4
Position on chr12 (Mb)

G

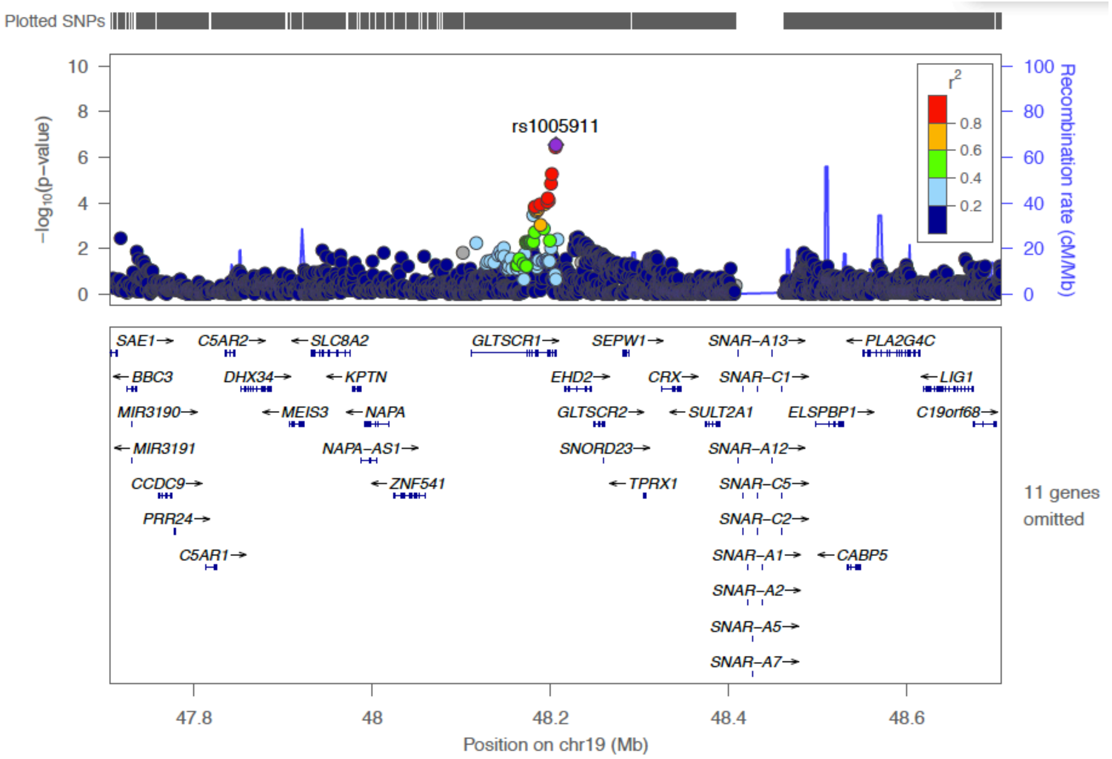
Plotted SNPs
rs1005911
r2
0.8
0.6
0.4
0.2
−log10(p−value)
Recombination rate (cM/Mb)
SAE1→ C5AR2→ ←SLC8A2 GLTSCR1→ SEPW1→ SNAR−A13→ ←PLA2G4C
←BBC3 DHX34→ ←KPTN EHD2→ CRX→ SNAR−C1→ ←LIG1
MIR3190→ ←MEIS3 ←NAPA GLTSCR2→ ←SULT2A1 ELSPBP1→ C19orf68→
←MIR3191 NAPA−AS1→ SNORD23→ SNAR−A12→
CCDC9→ ←ZNF541 ←TPRX1 SNAR−C5→
PRR24→ SNAR−C2→
C5AR1→ SNAR−A1→ ←CABP5
SNAR−A2→
SNAR−A5→
SNAR−A7→
11 genes omitted
47.8 48 48.2 48.4 48.6
Position on chr19 (Mb)

H

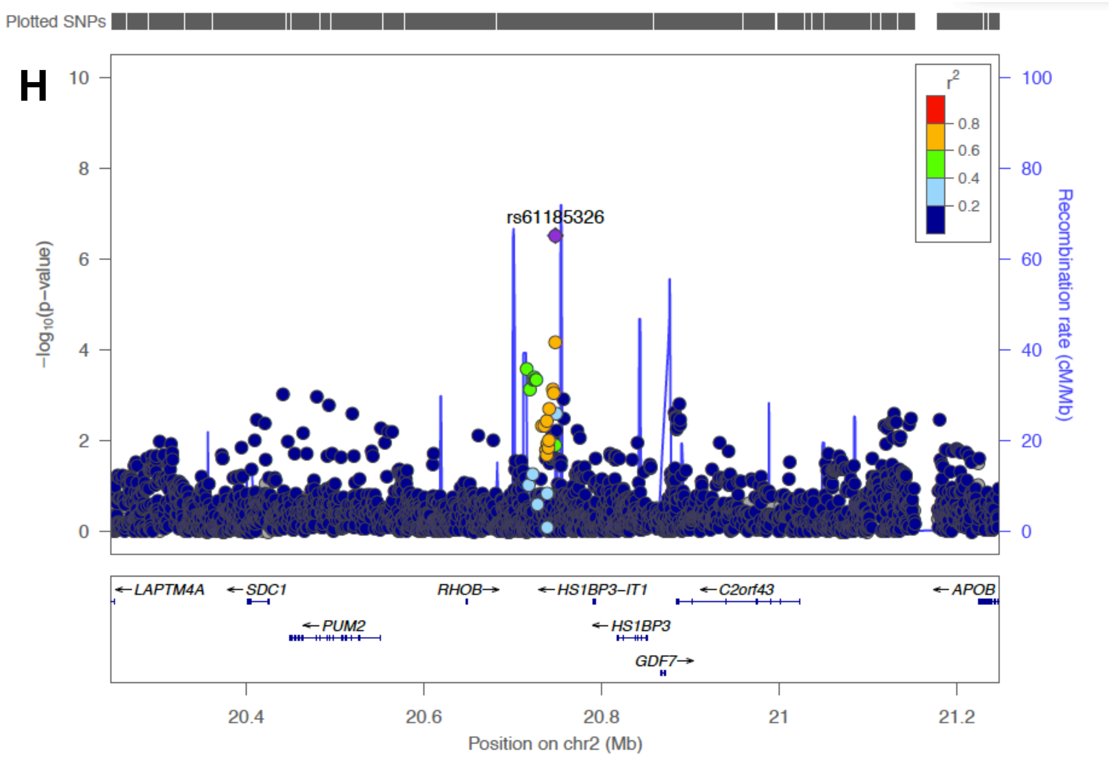
Plotted SNPs
rs61185326
r2
0.8
0.6
0.4
0.2
−log10(p−value)
Recombination rate (cM/Mb)
←LAPTM4A ←SDC1 RHOB→ ←HS1BP3−IT1 ←C2orf43 ←APOB
←PUM2 ←HS1BP3
GDF7→
20.4 20.6 20.8 21 21.2
Position on chr2 (Mb)

I

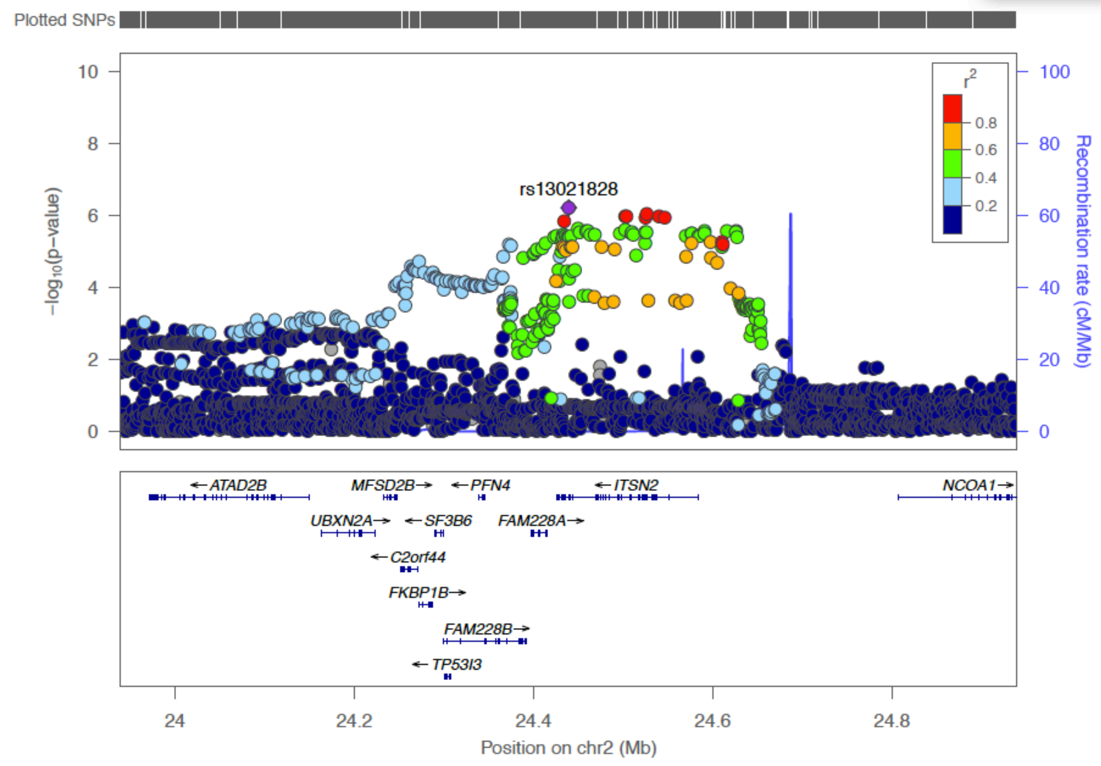

Plots are centred on the most significant SNP at each locus and flanked by the meta-analysis results for SNPs in the 500 kb region surrounding it. For each locus, the SNP with the lowest P value (topSNP) is depicted as a purple diamond; other SNPs are shaded according to their pairwise correlation (r^2) with the topSNP. The blue line represents the estimated recombination rates; the gene annotations are shown below the figure. SNPs were plotted against the EUR reference.

Supplementary Figure 5. Forest plots for the nine most strongly associated loci in the Discovery phase that were selected for replication.

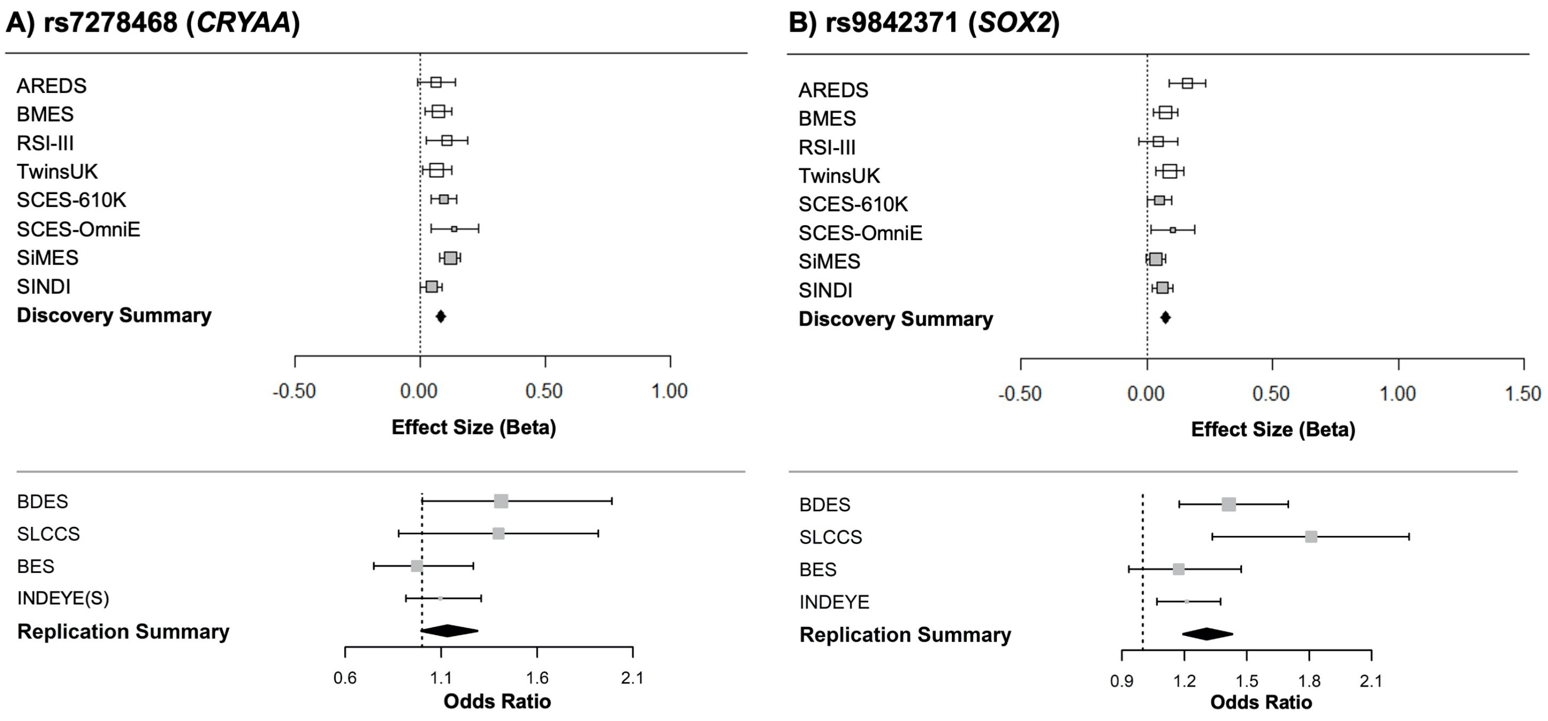

C) rs4936279 (*TMPRSS5*)

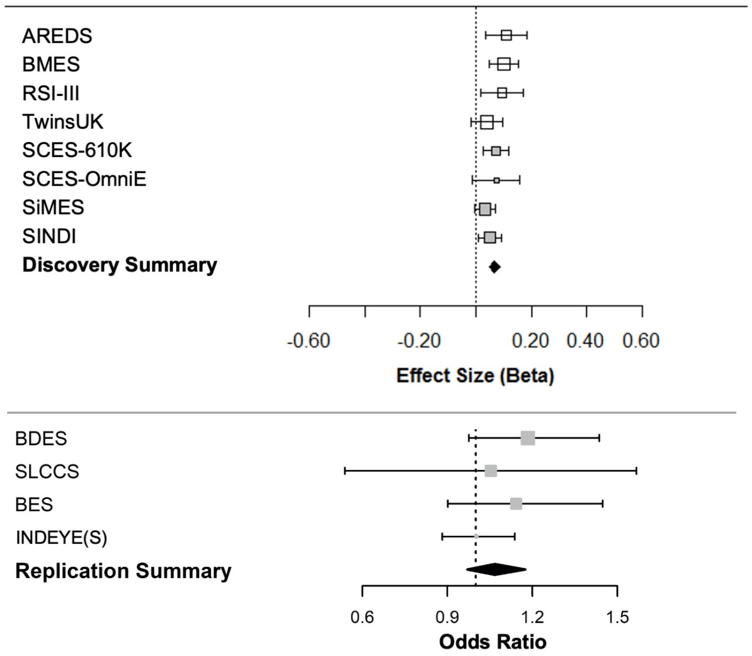

D) rs62149908 (*COMMD1*)

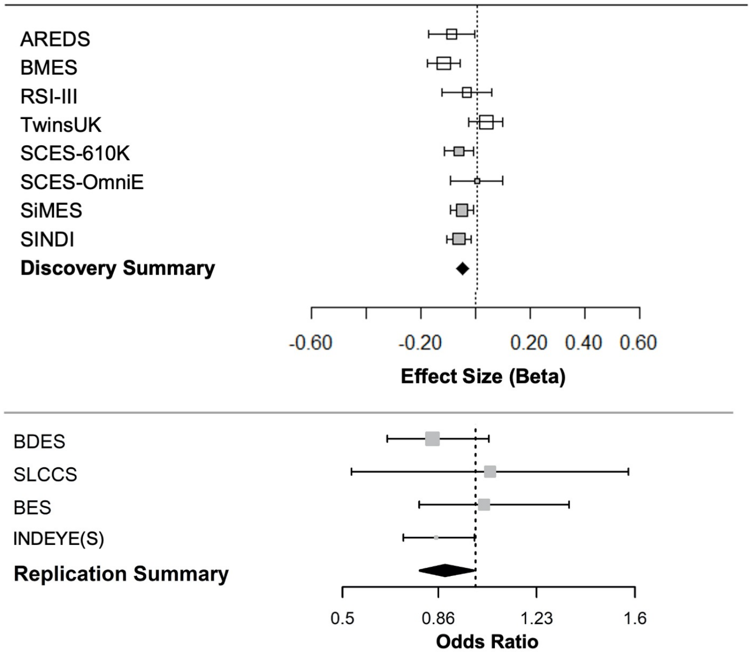

E) rs16823886 (*LINC01412*)

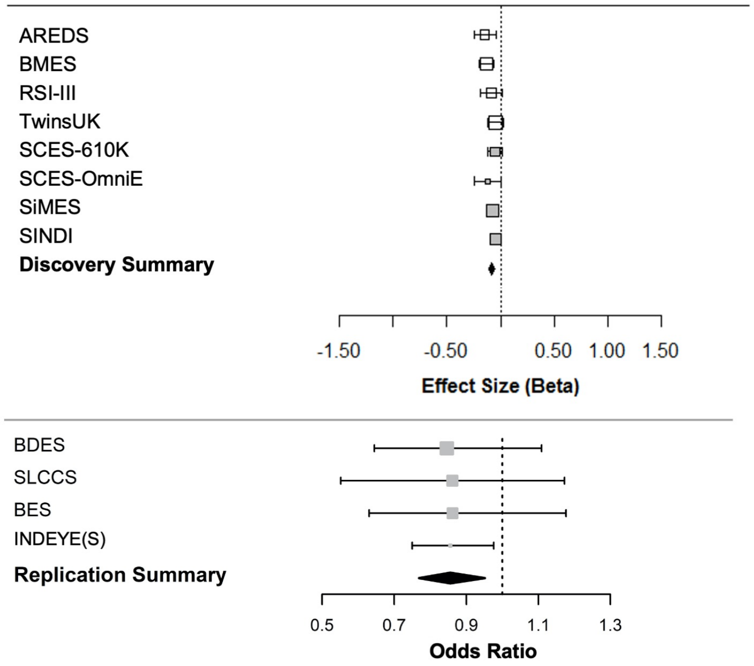

F) rs11067211 (*MMAB*)

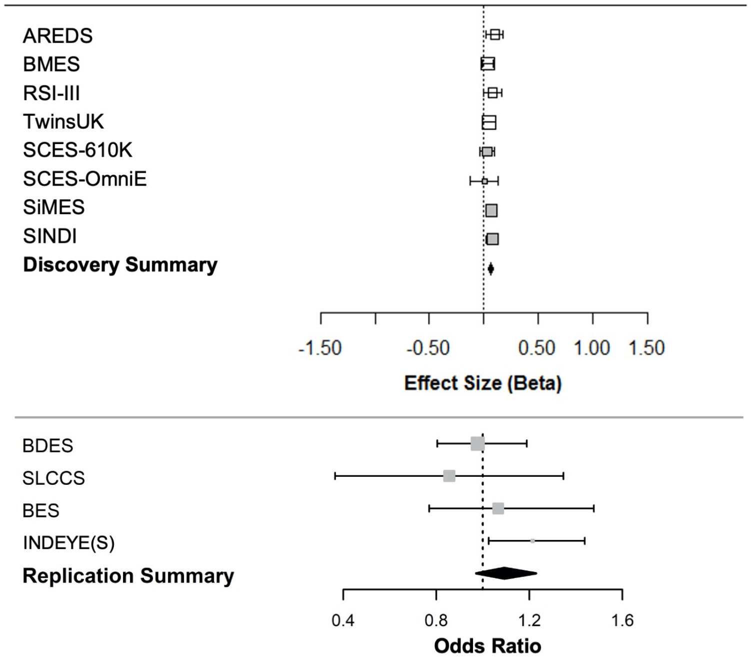

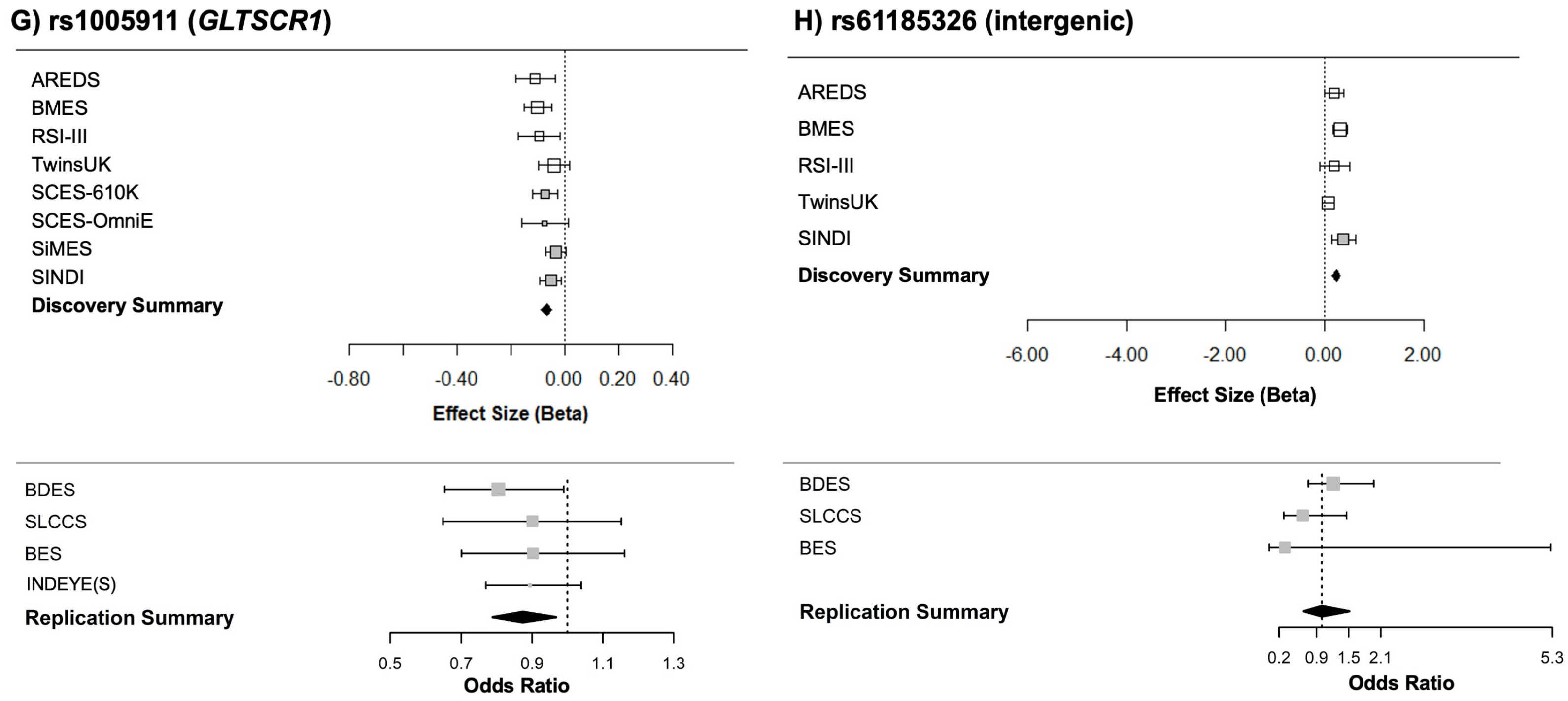
G) rs1005911 (GLTSCR1)
AREDS
BMES
RSI-III
TwinsUK
SCES-610K
SCES-OmniE
SiMES
SINDI
Discovery Summary
-0.80 -0.40 0.00 0.20 0.40
Effect Size (Beta)
BDES
SLCCS
BES
INDEYE(S)
Replication Summary
0.5 0.7 0.9 1.1 1.3
Odds Ratio
H) rs61185326 (intergenic)
AREDS
BMES
RSI-III
TwinsUK
SINDI
Discovery Summary
-6.00 -4.00 -2.00 0.00 2.00
Effect Size (Beta)
BDES
SLCCS
BES
Replication Summary
0.2 0.9 1.5 2.1 5.3
Odds Ratio

l) rs13021828 (*ITSN2*)

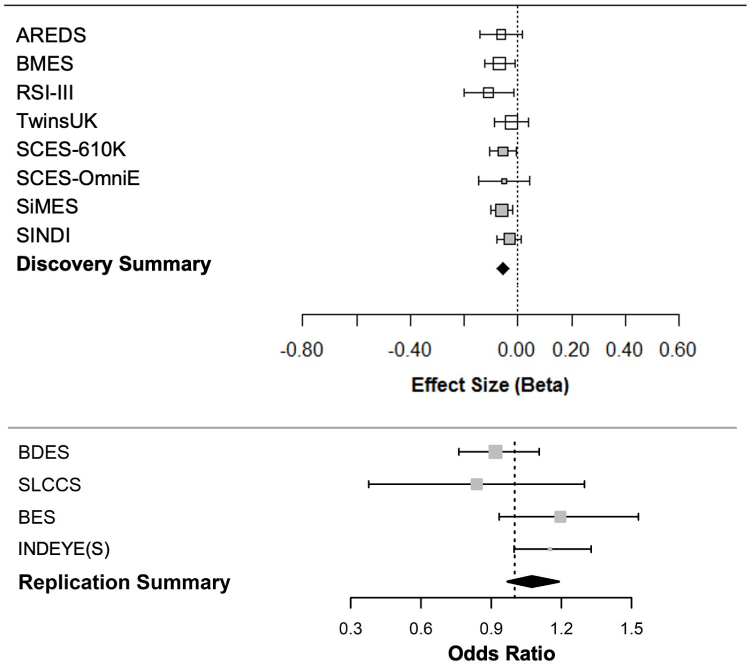

In the discovery phase, the effect size, beta is the linear regression coefficient for each additional copy of the effect allele under additive genetic model. In the replication phase. In the replication phase, the beta (ie, ln(odds ratio)), is the regression coefficient from logistic regression models (additive genetic model), and the odds ratio is The bars represent the standard error for the effect size estimate in the discovery phase and 95% confidence interval in the replication phase and squares are proportional to weights used in meta-analysis. In the discovery, the European ancestry cohorts have no shading and the Asian ancestry are shaded in gray. The overall effect is depicted by the black diamond in the Discovery phase and in the Replication phase separately.

Supplementary Figure 6. **FUMA annotation of the associated loci using various futures as follows: A) Legend B) *CRYAA,* C) *SOX2-OT*, D) *TMPRSS5*, E) *COMMD1* F) *LINC01412* G) *MMAB* H) *GLTSCR1* I) rs61185326 locus, and J) *ITSN2.***

A) Regulome DB

RegulomeDB Categorical Scores

Category	Description
	Likely to affect binding and linked to expression of a gene target
1a	eQTL + TF binding + matched TF motif + matched DNase footprint + DNase peak
1b	eQTL + TF binding + any motif + DNase footprint + DNase peak
1c	eQTL + TF binding + matched TF motif + DNase peak
1d	eQTL + TF binding + any motif + DNase peak
1e	eQTL + TF binding + matched TF motif
1f	eQTL + TF binding/DNase peak
	Likely to affect binding
2a	TF binding + matched TF motif + matched DNase footprint + DNase peak
2b	TF binding + any motif + DNase footprint + DNase peak
2c	TF binding + matched TF motif + DNase peak
	Less likely to affect binding
3a	TF binding + any motif + DNase peak
3b	TF binding + matched TF motif
	Minimal binding evidence
4	TF binding + DNase peak
5	TF binding or DNase peak
6	Motif hit
	No binding evidence
7	No evidence
NA	the variant does not exist in RegulomeDB

*External link to RegulomeDB from SNP table (when one of the SNPs is clicked) will open a new tab. rsID does not always match since RegulomeDB used dbSNP build 141 (the rsID in FUMA is dbSNP build 146). Genomic position (bp on hg19) shown in the link of RegulomeDB is the position shown in the SNP table - 1, since RegulomeDB used 0 based coordinate.

eQTLs

The color of eQTLs are arbitrary. When P-value is not available (i.e. for CMC eQTLs), -log10 FDR is plotted in stead of P-value.

SNPs colored grey in the plots

GWAS P-value: SNPs which are not in LD of any of significant independent lead SNPs in the selected region are colored grey.
CADD score: Only SNPs which are in LD of any of significant independent lead SNPs are displayed in the plot. Of those SNPs, SNPs which did not used for mapping (SNPs that were filtered by user defined parameters) are colored grey.
When positional mapping is performed, SNPs used for positional mapping are always colored non-grey colors.
When eQTL mapping is performed and eQTLs are plotted, SNPs used for eQTL mapping are also colored non-grey colors. If the option of eQTLs is not selected for the plot, SNPs which are not used for other mappings are colored grey even if they are used for eQTL mapping.
When chromatin interaction mapping is performed and chromatin interactions are plotted, SNPs used for chromatin interaction mapping are also colored non-grey colors. If the option of chromatin interactions is not selected for the plot, SNPs which are not used for other mappings are colored grey even if they are used for chromatin interaction mapping.
RegulomeDB score: Same as CADD score.
eQTLs: When eQTL mapping was performed and if there is any eQTL in the selected region, all eQTLs with user defined P-value threshold and tissue types are displayed. Of those eQTLs, eQTLs which did not used for eQTL mapping (eQTLs that were filtered by user defined parameters) are colored grey.

Color-code for genes

Red : Mapped genes. Genes mapped by positional mapping are always colored red. Genes mapped by eQTL mapping are colore red only when the option of eQTLs is selected for the plot, otherwise those genes are considered as non-mapped genes. Genes mapped by chromatin interaction are colored red only when the option of chromatin interactions is selected for the plot, otherwise those genes are considered as non-mapped genes.
Blue : Non-mapped protein-coding genes.
Dark grey : Non-mapped non-coding genes.

B)

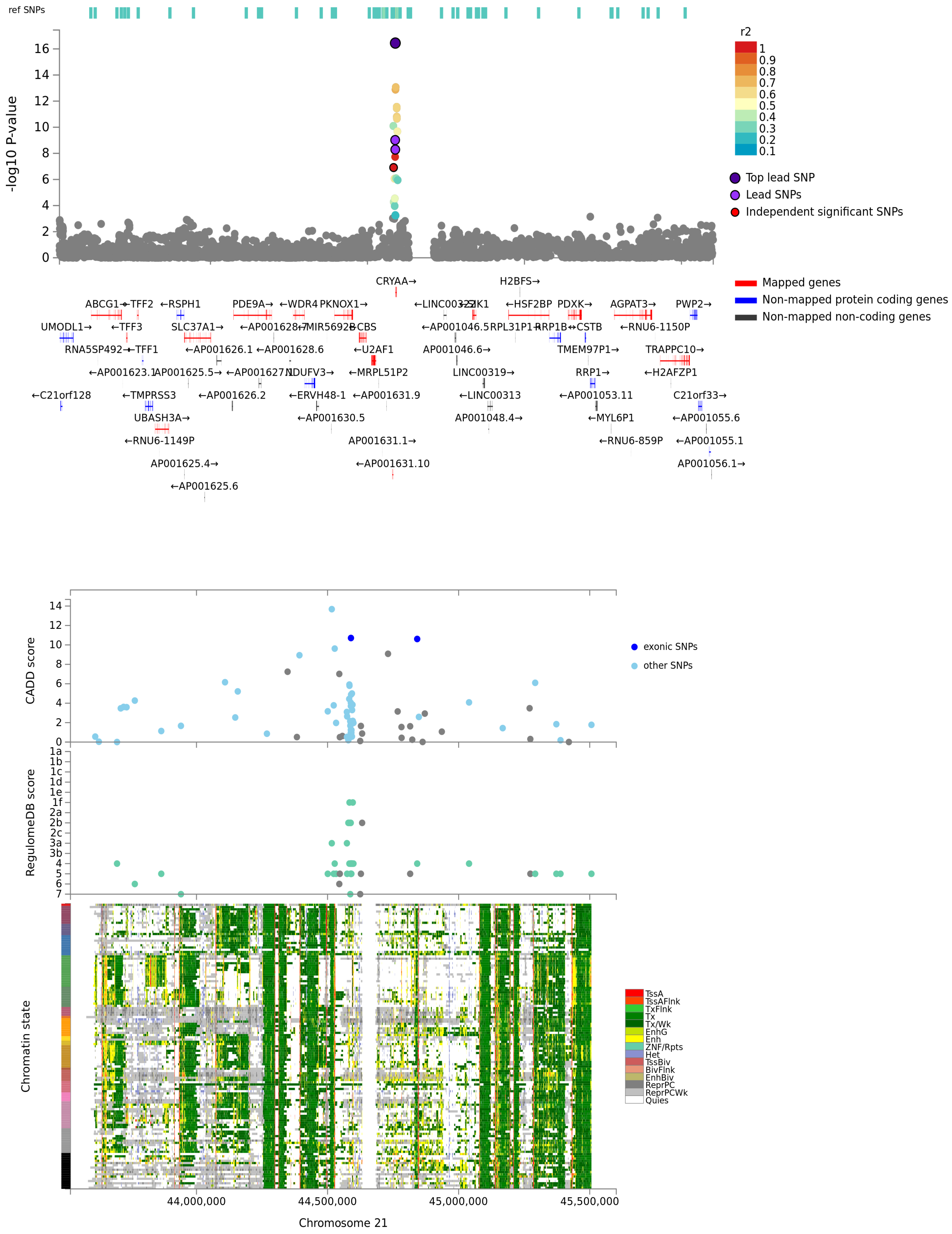
ref SNPs
-log10 P-value
r2
1
0.9
0.8
0.7
0.6
0.5
0.4
0.3
0.2
0.1
Top lead SNP
Lead SNPs
Independent significant SNPs
Mapped genes
Non-mapped protein coding genes
Non-mapped non-coding genes
CRYAA→
H2BFS→
ABCG1→
←TFF2
←RSPH1
PDE9A→
←WDR4
PKNOX1→
←LINC00322
←ZNF295-AS1
←HSF2BP
PDXK→
AGPAT3→
PWP2→
UMODL1→
←TFF3
SLC37A1→
←AP001628.7
←MIR5692B
←CBS
←AP001046.5
RPL31P1→
←RRP1B
←CSTB
←RNU6-1150P
RNA5SP492→
←TFF1
←AP001626.1
←AP001628.6
←U2AF1
AP001046.6→
TMEM97P1→
TRAPPC10→
←AP001623.1
AP001625.5→
←AP001627.1
NDUFV3→
←MRPL51P2
LINC00319→
RRP1→
←H2AFZP1
←C21orf128
←TMPRSS3
←AP001626.2
←ERVH48-1
←AP001631.9
←LINC00313
←AP001053.11
C21orf33→
UBASH3A→
←AP001630.5
AP001048.4→
←MYL6P1
←AP001055.6
←RNU6-1149P
AP001631.1→
←RNU6-859P
←AP001055.1
AP001625.4→
←AP001631.10
AP001056.1→
←AP001625.6
CADD score
exonic SNPs
other SNPs
RegulomeDB score
Chromatin state
TssA
TssAFlnk
TxFlnk
Tx
Tx/Wk
EnhG
Enh
ZNF/Rpts
Het
TssBiv
BivFlnk
EnhBiv
ReprPC
ReprPCWk
Quies
44,000,000
44,500,000
45,000,000
45,500,000
Chromosome 21

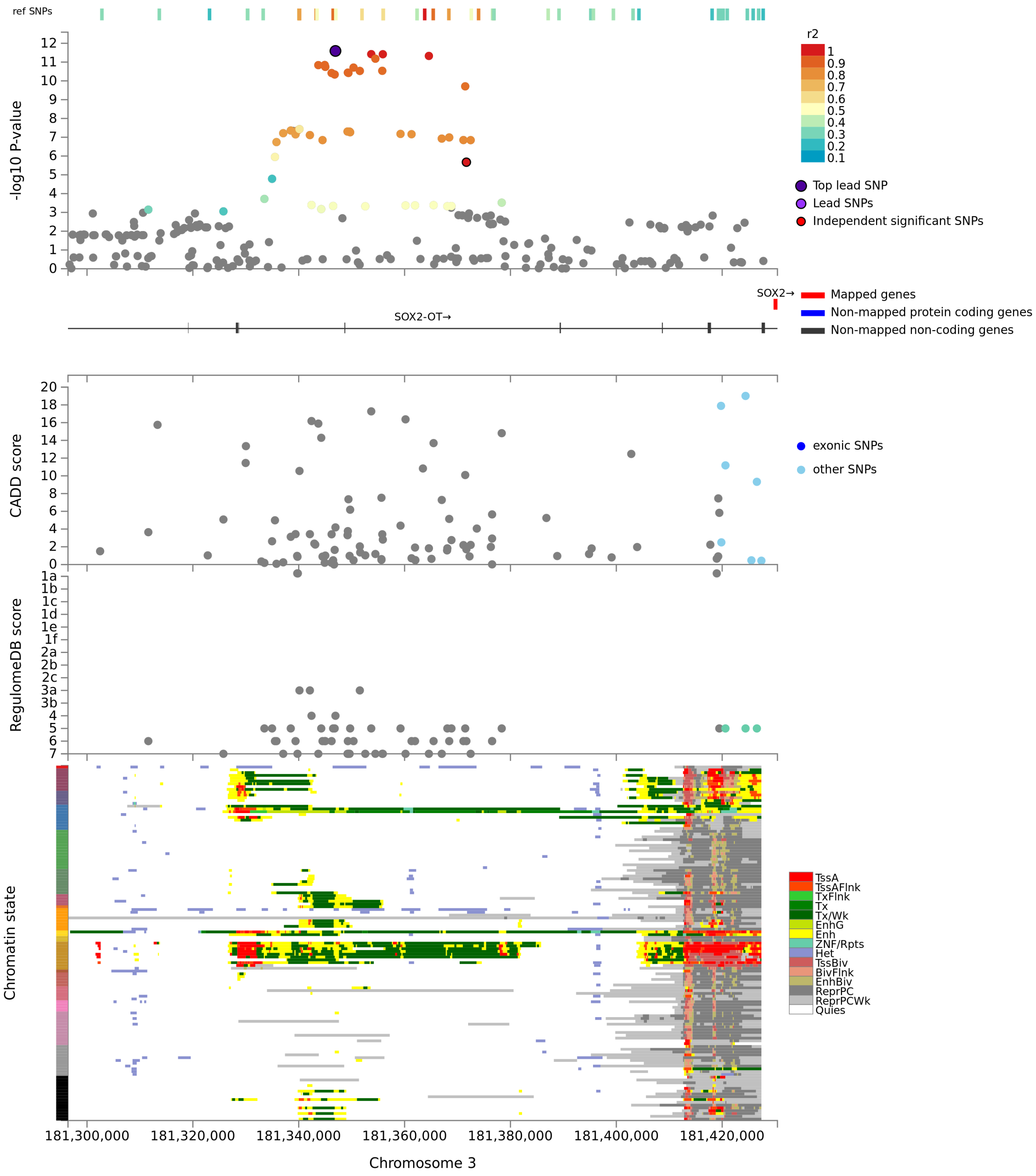

ref SNPs
-log10 P-value
r2
1
0.9
0.8
0.7
0.6
0.5
0.4
0.3
0.2
0.1
Top lead SNP
Lead SNPs
Independent significant SNPs
SOX2→
SOX2-OT→
Mapped genes
Non-mapped protein coding genes
Non-mapped non-coding genes
CADD score
exonic SNPs
other SNPs
RegulomeDB score
Chromatin state
TssA
TssAFlnk
TxFlnk
Tx
Tx/Wk
EnhG
Enh
ZNF/Rpts
Het
TssBiv
BivFlnk
EnhBiv
ReprPC
ReprPCWk
Quies
181,300,000
181,320,000
181,340,000
181,360,000
181,380,000
181,400,000
181,420,000
Chromosome 3

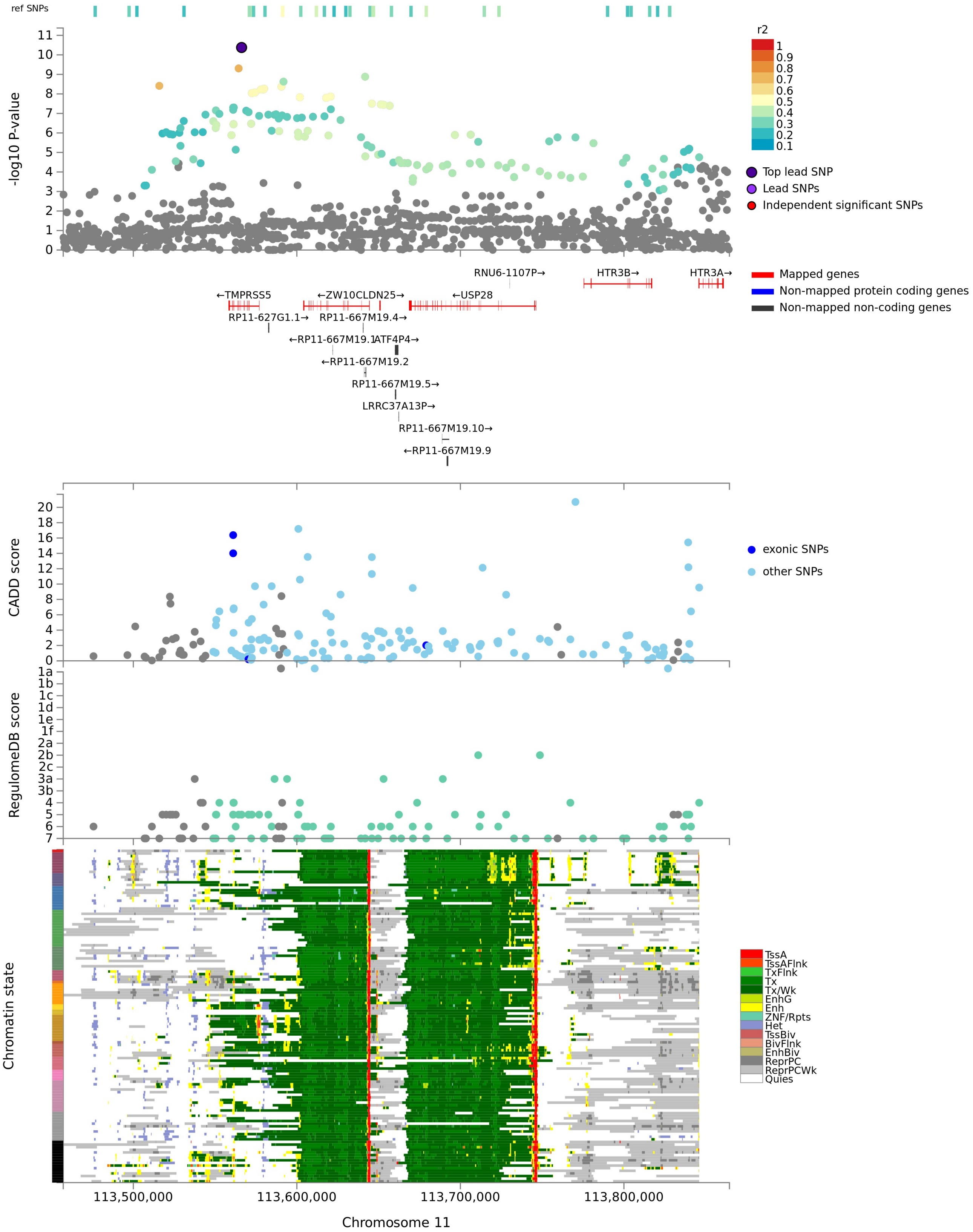

ref SNPs
-log10 P-value
r2
Top lead SNP
Lead SNPs
Independent significant SNPs
RNU6-1107P→
HTR3B→
HTR3A→
←TMPRSS5
←ZW10CLDN25→
←USP28
RP11-627G1.1→
RP11-667M19.4→
←RP11-667M19.1
ATF4P4→
←RP11-667M19.2
RP11-667M19.5→
LRRC37A13P→
RP11-667M19.10→
←RP11-667M19.9
Mapped genes
Non-mapped protein coding genes
Non-mapped non-coding genes
CADD score
exonic SNPs
other SNPs
RegulomeDB score
Chromatin state
TssA
TssAFlnk
TxFlnk
Tx
Tx/Wk
EnhG
Enh
ZNF/Rpts
Het
TssBiv
BivFlnk
EnhBiv
ReprPC
ReprPCWk
Quies
113,500,000
113,600,000
113,700,000
113,800,000
Chromosome 11

E)

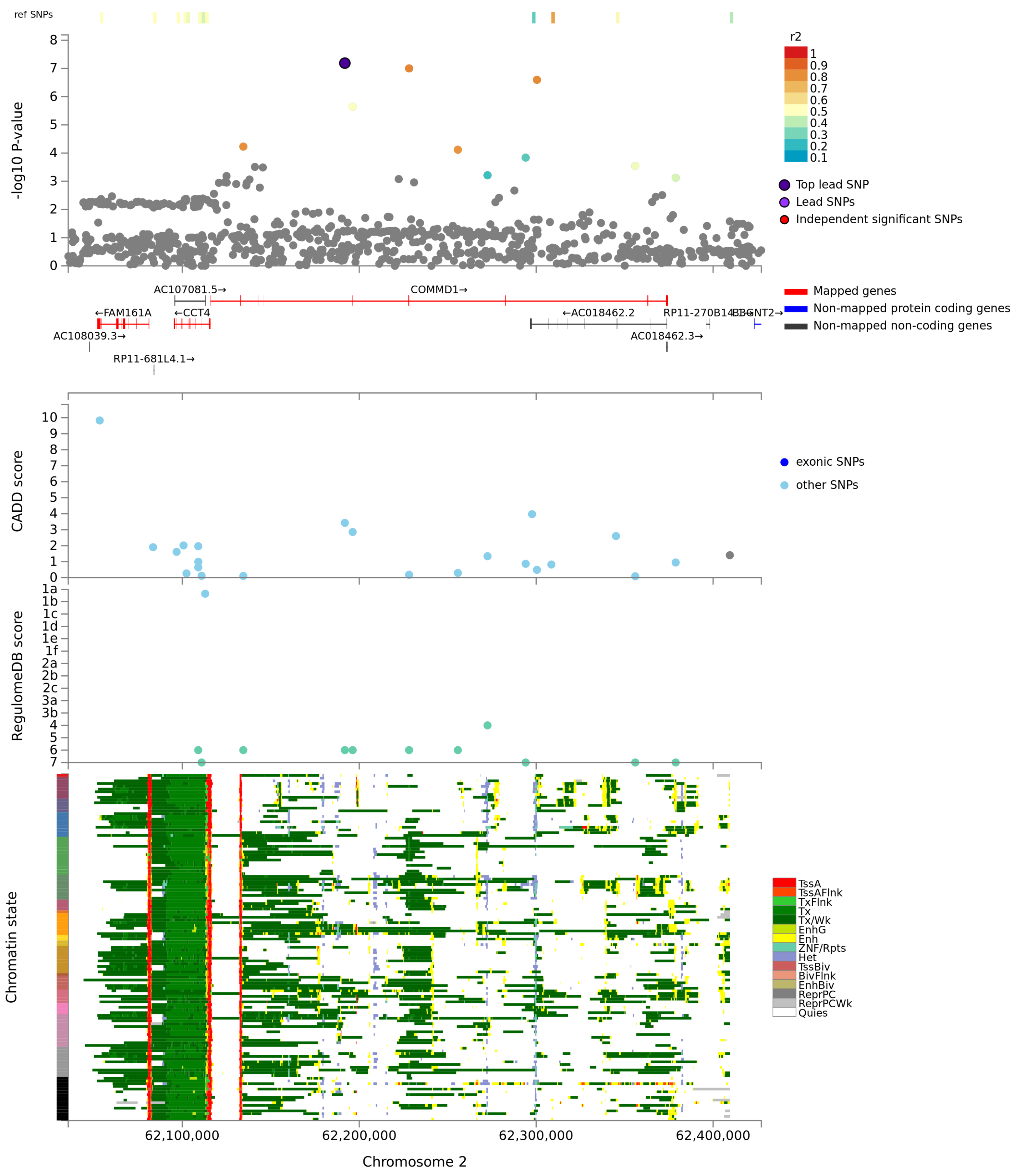
ref SNPs
-log10 P-value
r2
1
0.9
0.8
0.7
0.6
0.5
0.4
0.3
0.2
0.1
Top lead SNP
Lead SNPs
Independent significant SNPs
AC107081.5→
COMMD1→
←FAM161A
←CCT4
←AC018462.2
RP11-270B14.3
B3GNT2→
AC108039.3→
AC018462.3→
RP11-681L4.1→
Mapped genes
Non-mapped protein coding genes
Non-mapped non-coding genes
CADD score
exonic SNPs
other SNPs
RegulomeDB score
Chromatin state
TssA
TssAFlnk
TxFlnk
Tx
Tx/Wk
EnhG
Enh
ZNF/Rpts
Het
TssBiv
BivFlnk
EnhBiv
ReprPC
ReprPCWk
Quies
62,100,000
62,200,000
62,300,000
62,400,000
Chromosome 2

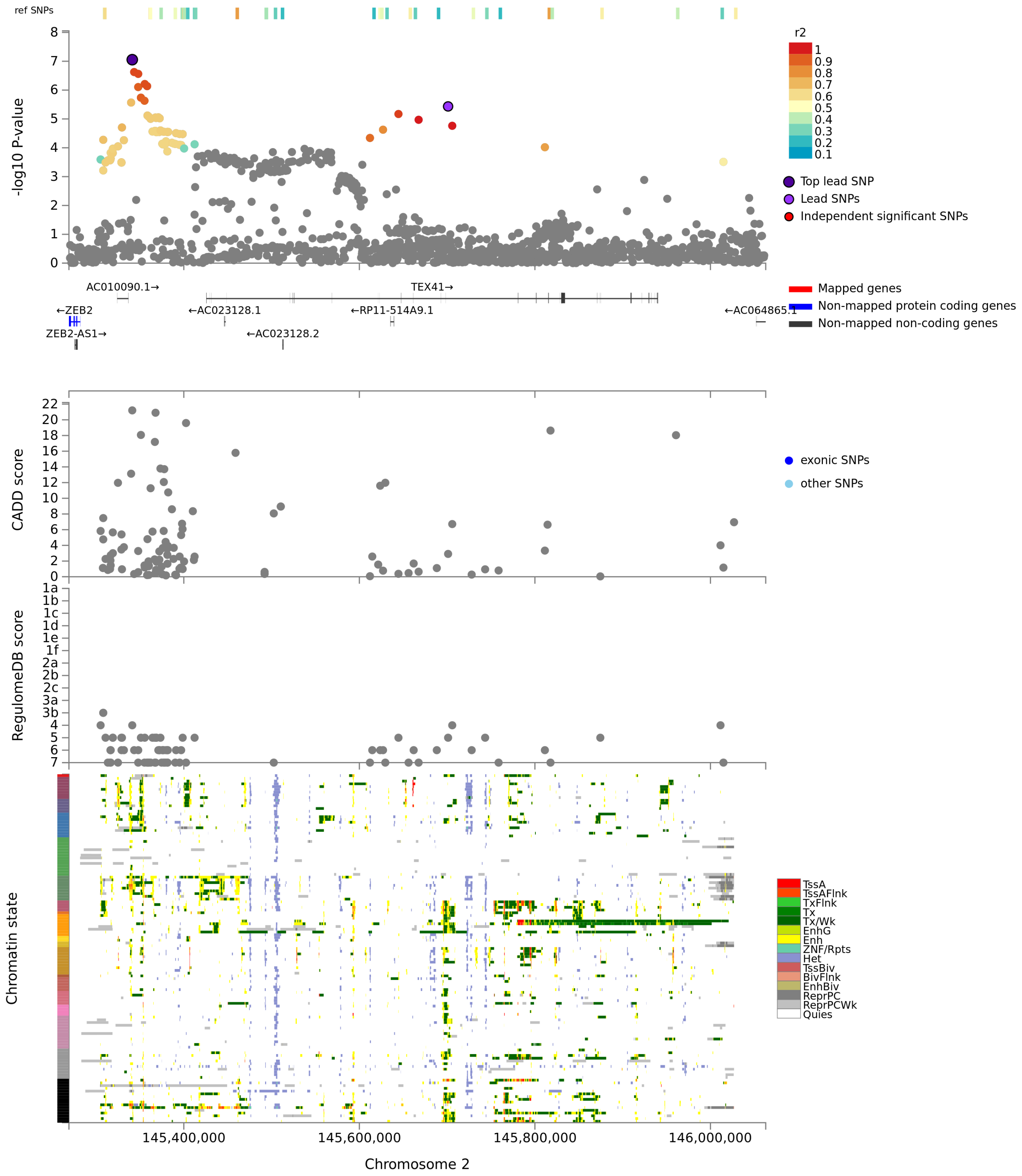

ref SNPs
r2
1
0.9
0.8
0.7
0.6
0.5
0.4
0.3
0.2
0.1
Top lead SNP
Lead SNPs
Independent significant SNPs
-log10 P-value
AC010090.1→
TEX41→
←ZEB2
←AC023128.1
←RP11-514A9.1
←AC064865.1
ZEB2-AS1→
←AC023128.2
Mapped genes
Non-mapped protein coding genes
Non-mapped non-coding genes
CADD score
exonic SNPs
other SNPs
RegulomeDB score
Chromatin state
TssA
TssAFlnk
TxFlnk
Tx
Tx/Wk
EnhG
Enh
ZNF/Rpts
Het
TssBiv
BivFlnk
EnhBiv
ReprPC
ReprPCWk
Quies
145,400,000
145,600,000
145,800,000
146,000,000
Chromosome 2

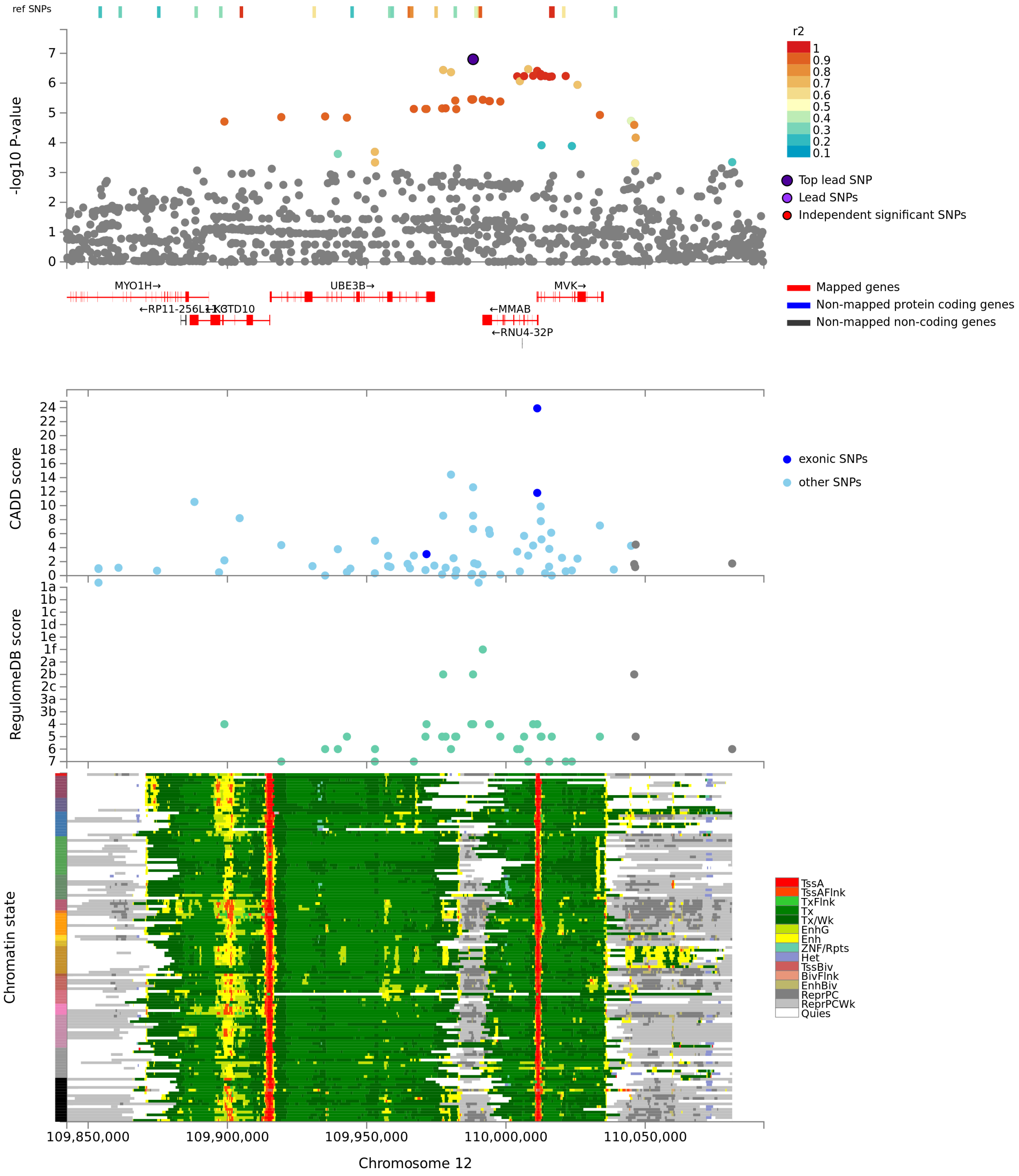
ref SNPs
-log10 P-value
r2
1
0.9
0.8
0.7
0.6
0.5
0.4
0.3
0.2
0.1
Top lead SNP
Lead SNPs
Independent significant SNPs
MYO1H→
UBE3B→
MVK→
←RP11-256L11.3
KCTD10
←MMAB
←RNU4-32P
Mapped genes
Non-mapped protein coding genes
Non-mapped non-coding genes
CADD score
exonic SNPs
other SNPs
RegulomeDB score
1a
1b
1c
1d
1e
1f
2a
2b
2c
3a
3b
4
5
6
7
Chromatin state
TssA
TssAFlnk
TxFlnk
Tx
TxWk
EnhG
Enh
ZNF/Rpts
Het
TssBiv
BivFlnk
EnhBiv
ReprPC
ReprPCWk
Quies
109,850,000
109,900,000
109,950,000
110,000,000
110,050,000
Chromosome 12

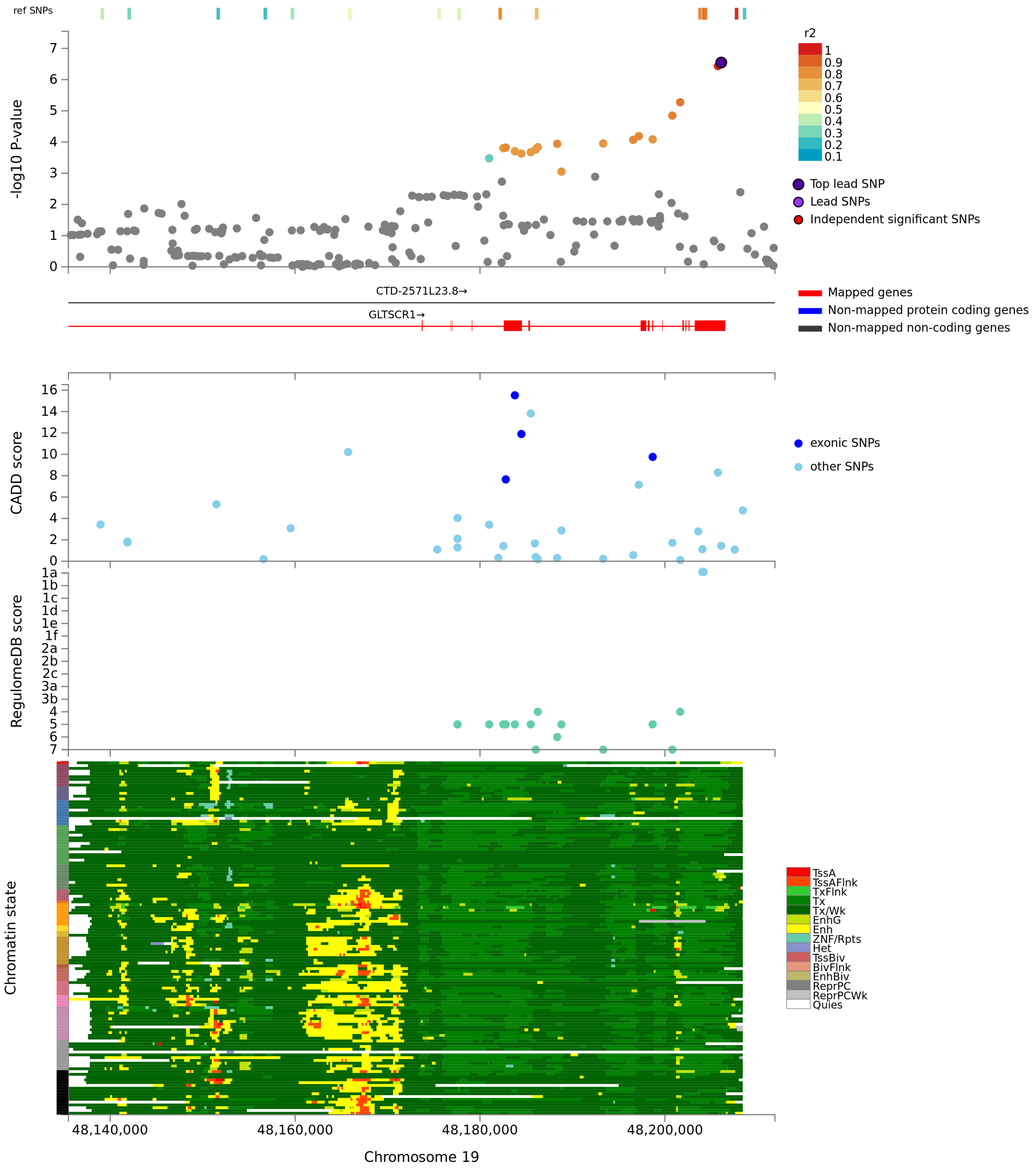
ref SNPs
-log10 P-value
r2
1
0.9
0.8
0.7
0.6
0.5
0.4
0.3
0.2
0.1
Top lead SNP
Lead SNPs
Independent significant SNPs
CTD-2571L23.8→
GLTSCR1→
Mapped genes
Non-mapped protein coding genes
Non-mapped non-coding genes
CADD score
exonic SNPs
other SNPs
RegulomeDB score
Chromatin state
TssA
TssAFlnk
TxFlnk
Tx
TxWk
EnhG
Enh
ZNF/Rpts
Het
TssBiv
BivFlnk
EnhBiv
ReprPC
ReprPCWk
Quies
48,140,000
48,160,000
48,180,000
48,200,000
Chromosome 19

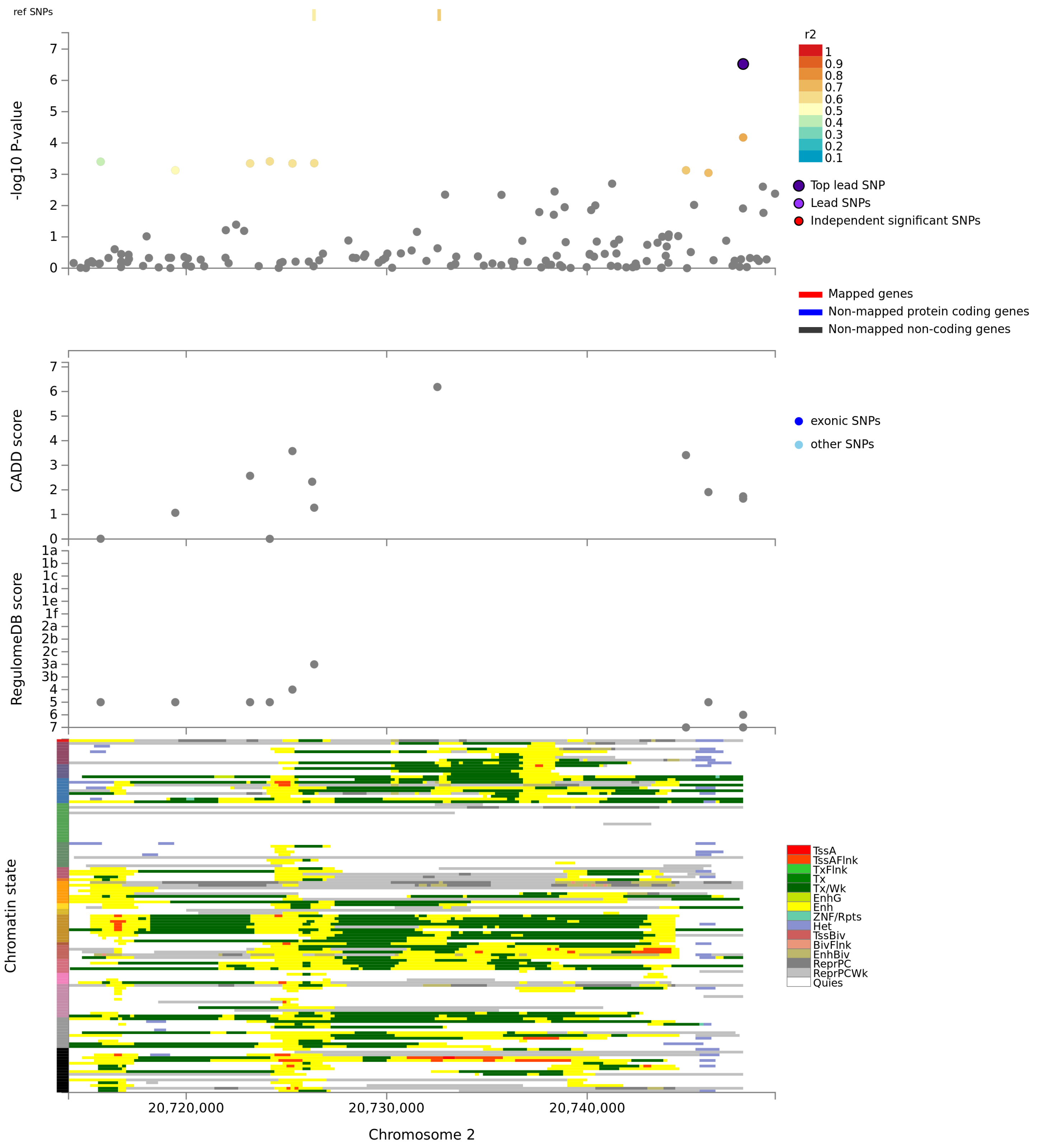
ref SNPs
-log10 P-value
r2
1
0.9
0.8
0.7
0.6
0.5
0.4
0.3
0.2
0.1
Top lead SNP
Lead SNPs
Independent significant SNPs
Mapped genes
Non-mapped protein coding genes
Non-mapped non-coding genes
CADD score
exonic SNPs
other SNPs
RegulomeDB score
Chromatin state
TssA
TssAFlnk
TxFlnk
Tx
Tx/Wk
EnhG
Enh
ZNF/Rpts
Het
TssBiv
BivFlnk
EnhBiv
ReprPC
ReprPCWk
Quies
20,720,000
20,730,000
20,740,000
Chromosome 2

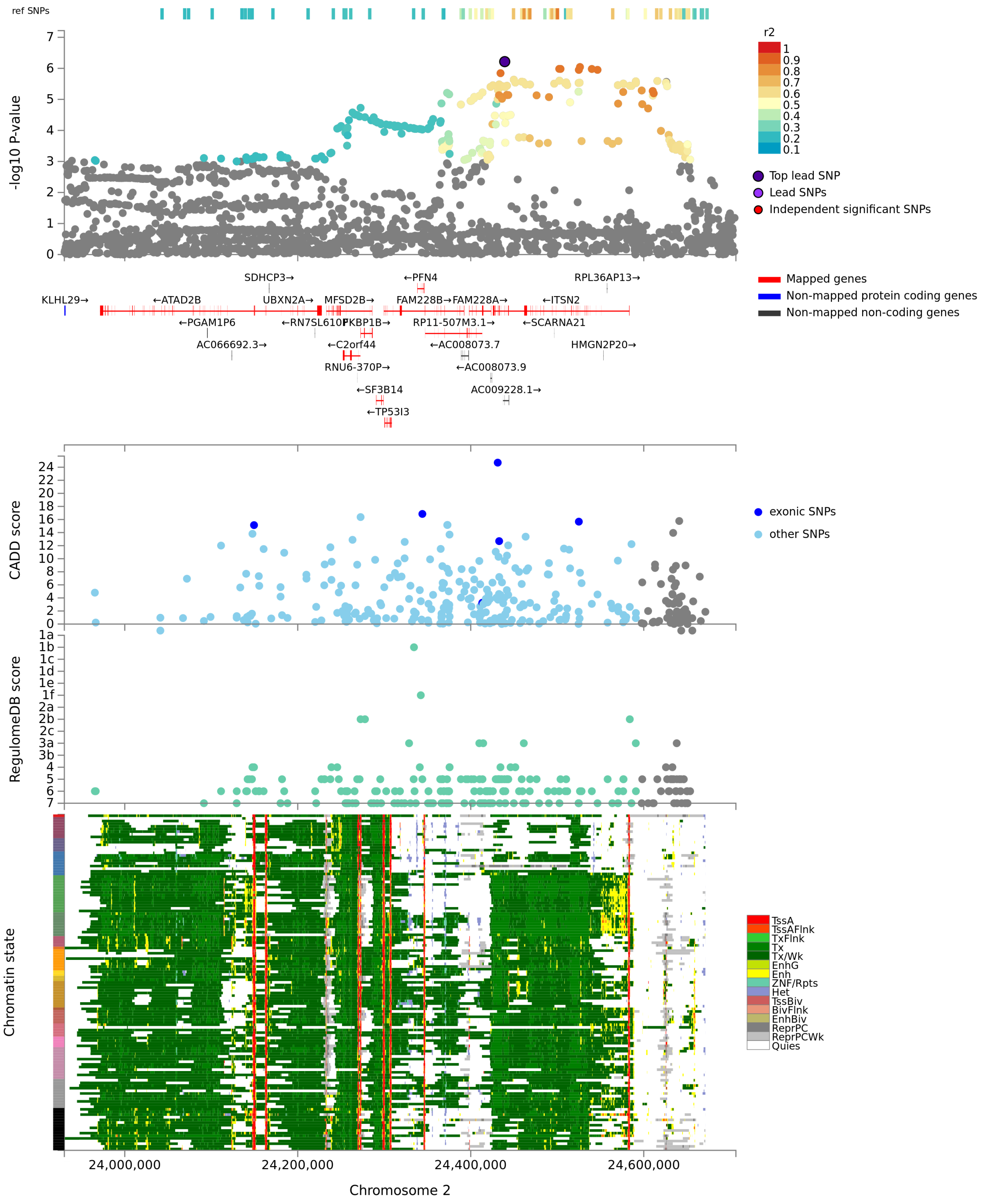

ref SNPs
-log10 P-value
r2
Top lead SNP
Lead SNPs
Independent significant SNPs
SDHCP3→
←PFN4
RPL36AP13→
KLHL29→
←ATAD2B
UBXN2A→
MFSD2B→
FAM228B→
FAM228A→
←ITSN2
←PGAM1P6
←RN7SL610P
FKBP1B→
RP11-507M3.1→
←SCARNA21
AC066692.3→
←C2orf44
←AC008073.7
HMGN2P20→
RNU6-370P→
←AC008073.9
←SF3B14
AC009228.1→
←TP53I3
Mapped genes
Non-mapped protein coding genes
Non-mapped non-coding genes
CADD score
exonic SNPs
other SNPs
RegulomeDB score
Chromatin state
TssA
TssAFlnk
TxFlnk
Tx
Tx/Wk
EnhG
Enh
ZNF/Rpts
Het
TssBiv
BivFlnk
EnhBiv
ReprPC
ReprPCWk
Quies
24,000,000
24,200,000
24,400,000
24,600,000
Chromosome 2

Supplementary Figure 7. Replication stage power analysis.

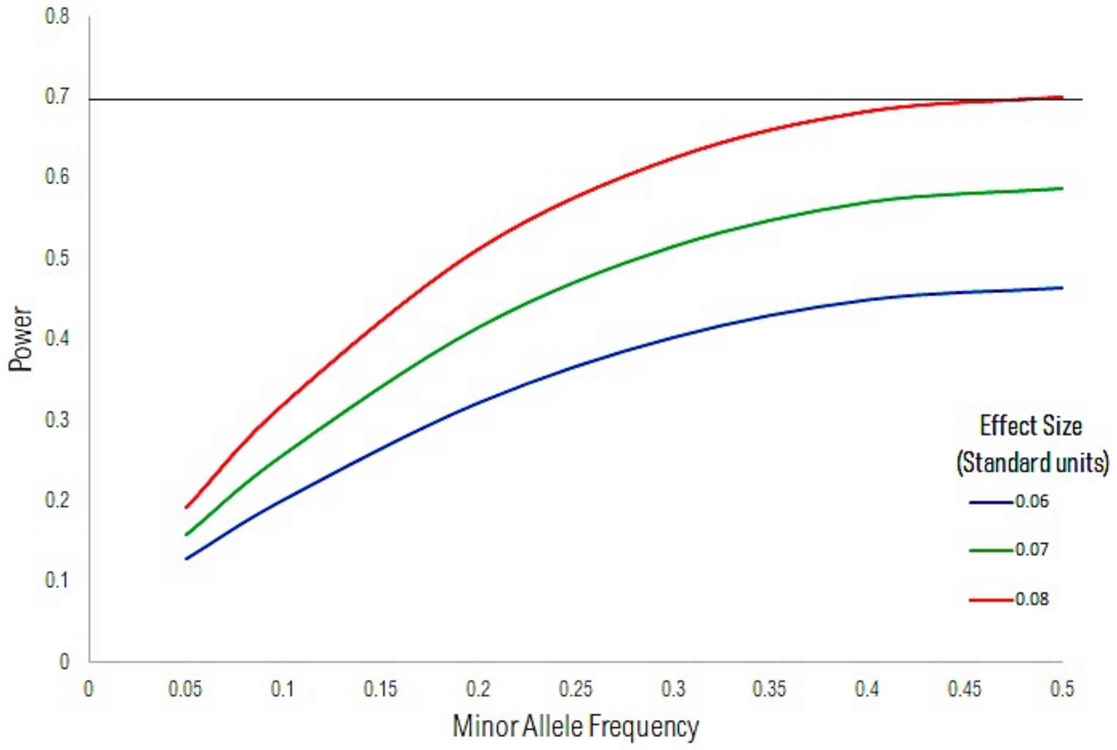

The figure shows the power the replication cohorts had to detect effects as the ones observed in the Discovery phase for a range of allele frequencies.

Supplementary Figure 8. Results from LD-score regression analysis.

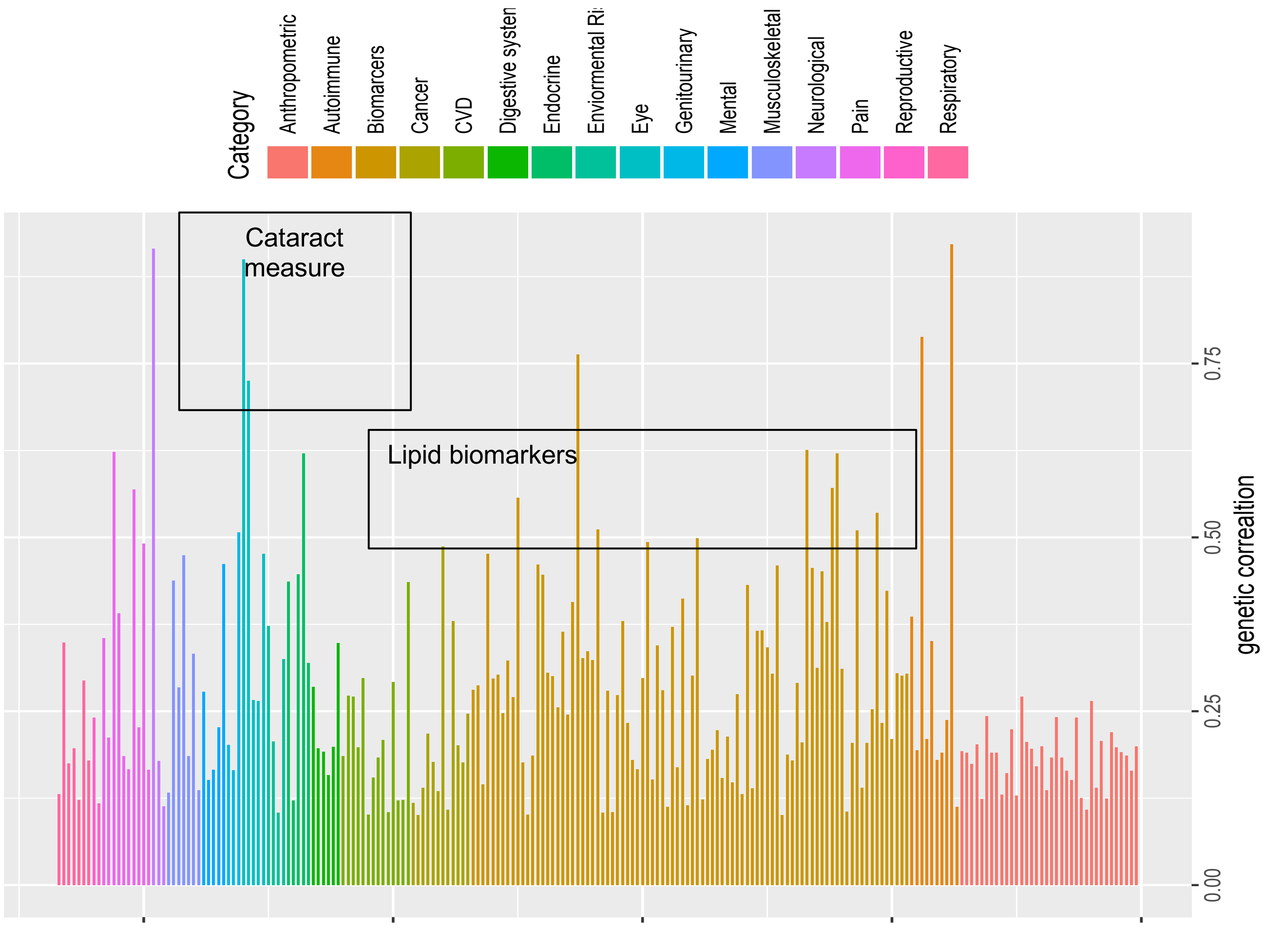

This figure shows the genetic correlation (calculated using LD-score regression) between cataract and various disease and biomarkers.

Supplementary Figure 9. Results from Open Targets Genetics: A) SNP-based PheWAS; B) Gene-centric co-localization

A)

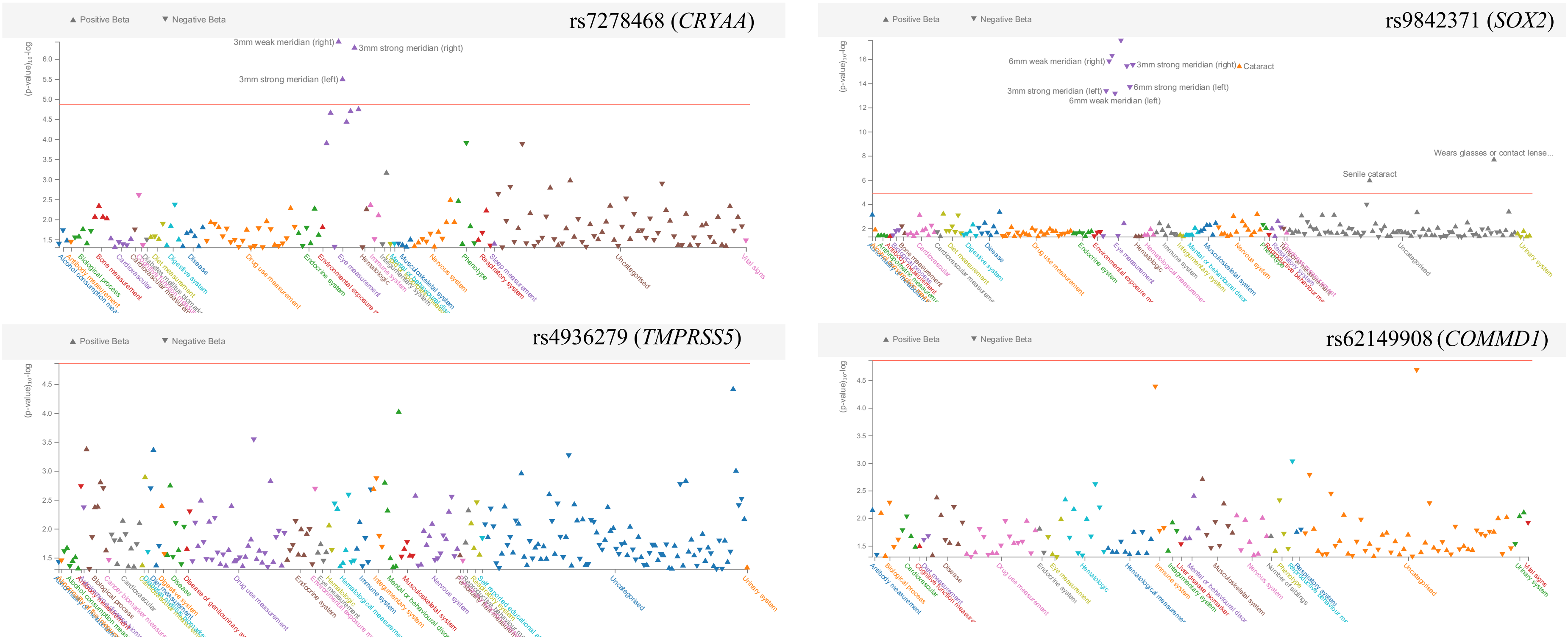

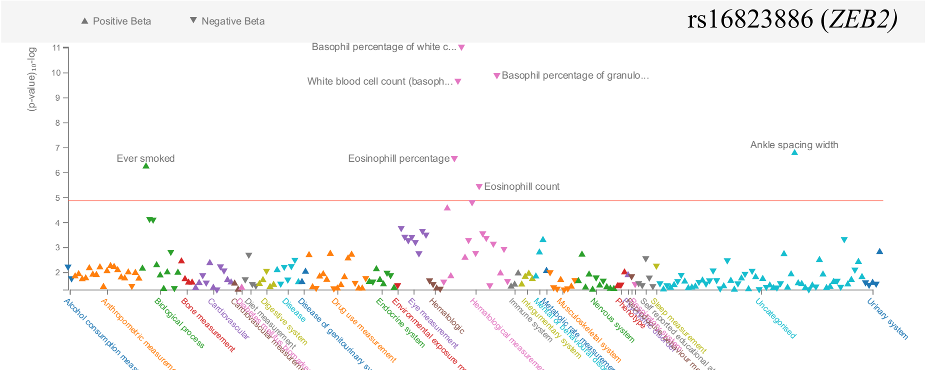
rs16823886 (ZEB2)
Positive Beta
Negative Beta
(p-value)₁₀-log
Basophil percentage of white c...
White blood cell count (basoph...
Basophil percentage of granulo...
Ever smoked
Eosinophil percentage
Eosinophil count
Ankle spacing width
Uncategorised
Urinary system

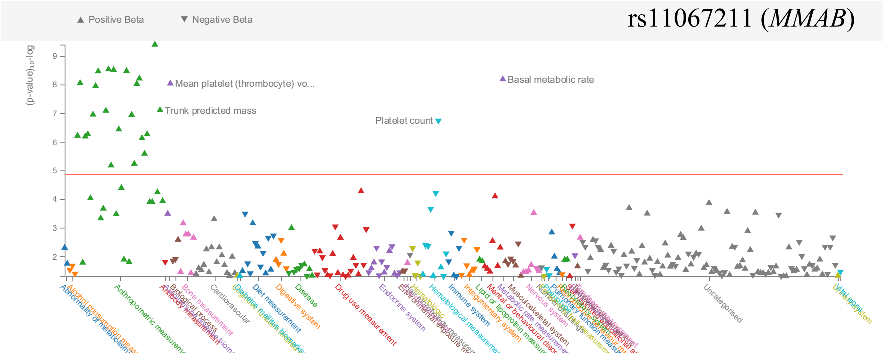
rs11067211 (MMAB)
Positive Beta
Negative Beta
Mean platelet (thrombocyte) vo...
Trunk predicted mass
Basal metabolic rate
Platelet count
Uncategorised

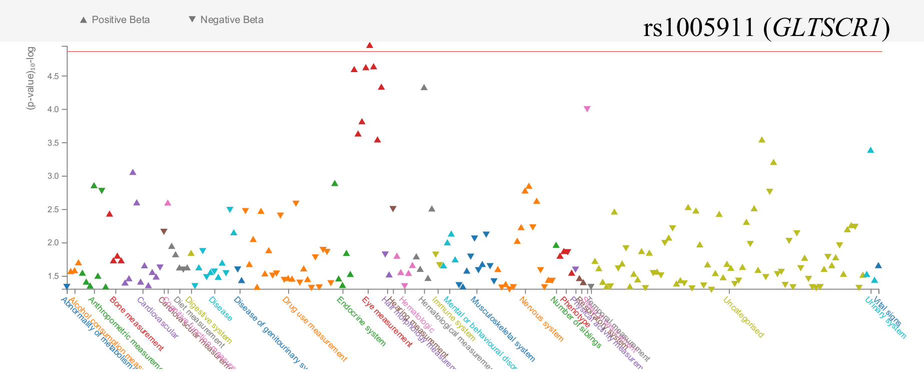
rs1005911 (GLTSCR1)
Positive Beta
Negative Beta
Uncategorised

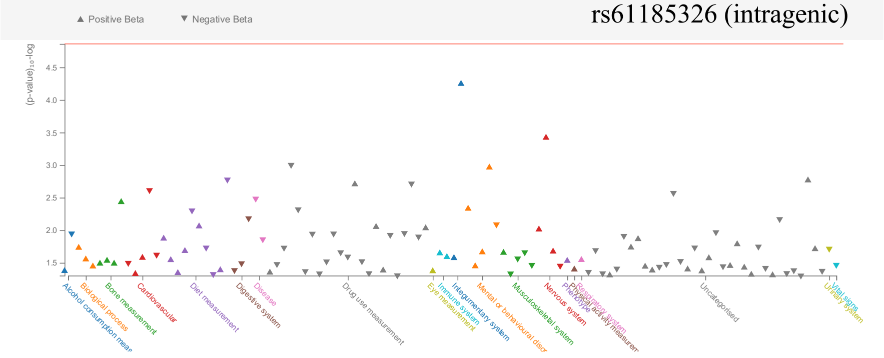
rs61185326 (intragenic)
Positive Beta
Negative Beta
Uncategorised

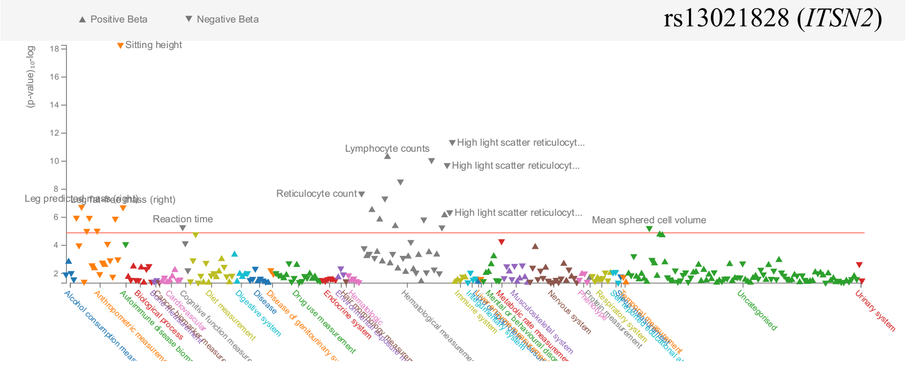
rs13021828 (ITSN2)
Positive Beta
Negative Beta
Sitting height
Lymphocyte counts
High light scatter reticulocyt...
High light scatter reticulocyt...
Reticulocyte count
High light scatter reticulocyt...
Reaction time
Mean sphered cell volume
Uncategorised
Urinary system

B)

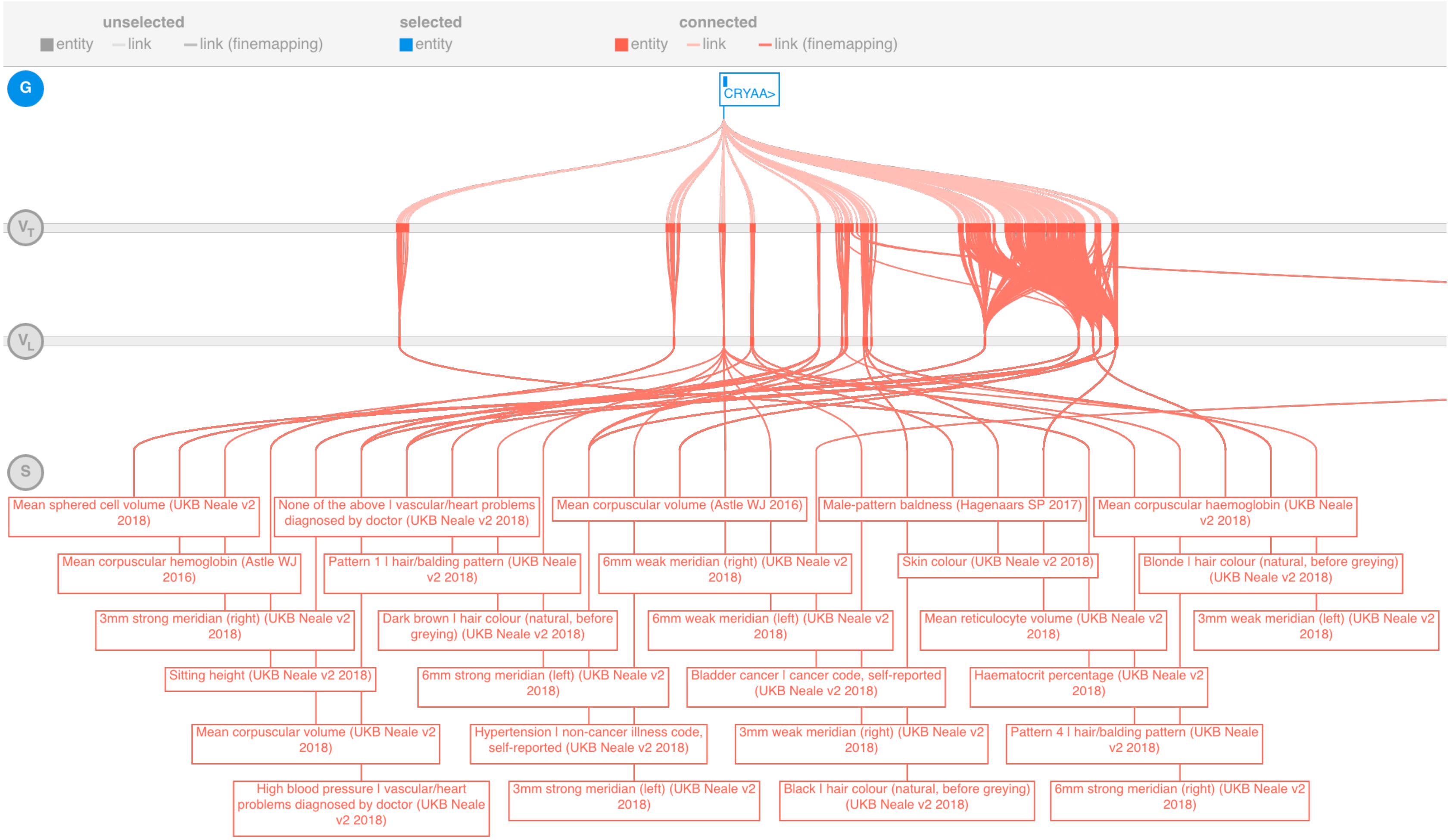
unselected
entity
link
link (finemapping)
selected
entity
connected
entity
link
link (finemapping)
G
CRYAA>
V_T
V_L
S
Mean sphered cell volume (UKB Neale v2 2018)
None of the above | vascular/heart problems diagnosed by doctor (UKB Neale v2 2018)
Mean corpuscular volume (Astle WJ 2016)
Male-pattern baldness (Hagenaars SP 2017)
Mean corpuscular haemoglobin (UKB Neale v2 2018)
Mean corpuscular hemoglobin (Astle WJ 2016)
Pattern 1 | hair/balding pattern (UKB Neale v2 2018)
6mm weak meridian (right) (UKB Neale v2 2018)
Skin colour (UKB Neale v2 2018)
Blonde | hair colour (natural, before greying) (UKB Neale v2 2018)
3mm strong meridian (right) (UKB Neale v2 2018)
Dark brown | hair colour (natural, before greying) (UKB Neale v2 2018)
6mm weak meridian (left) (UKB Neale v2 2018)
Mean reticulocyte volume (UKB Neale v2 2018)
3mm weak meridian (left) (UKB Neale v2 2018)
Sitting height (UKB Neale v2 2018)
6mm strong meridian (left) (UKB Neale v2 2018)
Bladder cancer | cancer code, self-reported (UKB Neale v2 2018)
Haematocrit percentage (UKB Neale v2 2018)
Mean corpuscular volume (UKB Neale v2 2018)
Hypertension | non-cancer illness code, self-reported (UKB Neale v2 2018)
3mm weak meridian (right) (UKB Neale v2 2018)
Pattern 4 | hair/balding pattern (UKB Neale v2 2018)
High blood pressure | vascular/heart problems diagnosed by doctor (UKB Neale v2 2018)
3mm strong meridian (left) (UKB Neale v2 2018)
Black | hair colour (natural, before greying) (UKB Neale v2 2018)
6mm strong meridian (right) (UKB Neale v2 2018)

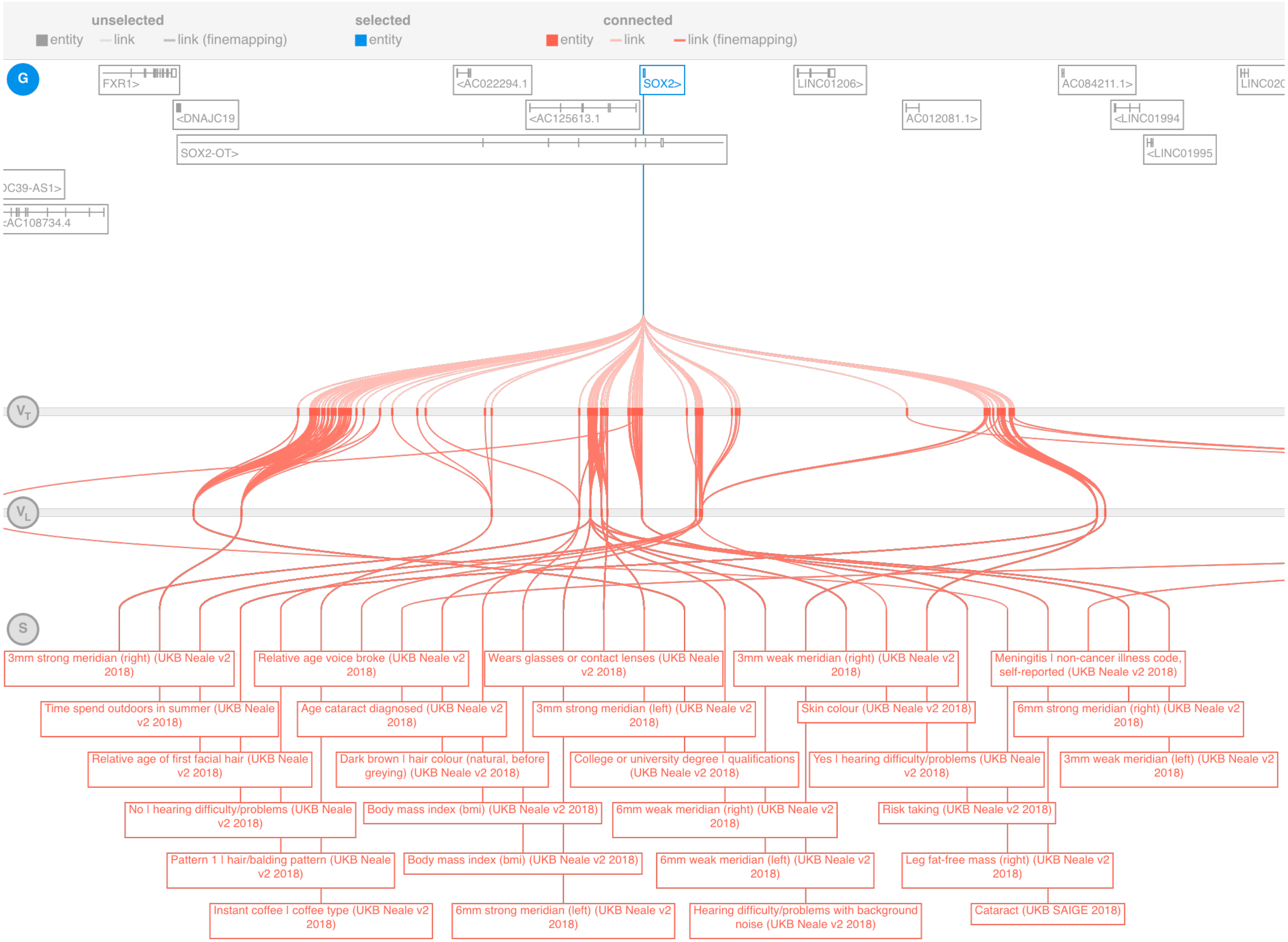
unselected
entity
link
link (finemapping)
selected
entity
connected
entity
link
link (finemapping)
G
FXR1>
<DNAJC19
<AC022294.1
<AC125613.1
SOX2>
SOX2-OT>
LINC01206>
AC012081.1>
AC084211.1>
<LINC01994
<LINC01995
)C39-AS1>
<AC108734.4
V_T
V_L
S
3mm strong meridian (right) (UKB Neale v2 2018)
Relative age voice broke (UKB Neale v2 2018)
Wears glasses or contact lenses (UKB Neale v2 2018)
3mm weak meridian (right) (UKB Neale v2 2018)
Meningitis | non-cancer illness code, self-reported (UKB Neale v2 2018)
Time spend outdoors in summer (UKB Neale v2 2018)
Age cataract diagnosed (UKB Neale v2 2018)
3mm strong meridian (left) (UKB Neale v2 2018)
Skin colour (UKB Neale v2 2018)
6mm strong meridian (right) (UKB Neale v2 2018)
Relative age of first facial hair (UKB Neale v2 2018)
Dark brown | hair colour (natural, before greying) (UKB Neale v2 2018)
College or university degree | qualifications (UKB Neale v2 2018)
Yes | hearing difficulty/problems (UKB Neale v2 2018)
3mm weak meridian (left) (UKB Neale v2 2018)
No | hearing difficulty/problems (UKB Neale v2 2018)
Body mass index (bmi) (UKB Neale v2 2018)
6mm weak meridian (right) (UKB Neale v2 2018)
Risk taking (UKB Neale v2 2018)
Pattern 1 | hair/balding pattern (UKB Neale v2 2018)
Body mass index (bmi) (UKB Neale v2 2018)
6mm weak meridian (left) (UKB Neale v2 2018)
Leg fat-free mass (right) (UKB Neale v2 2018)
Instant coffee | coffee type (UKB Neale v2 2018)
6mm strong meridian (left) (UKB Neale v2 2018)
Hearing difficulty/problems with background noise (UKB Neale v2 2018)
Cataract (UKB SAIGE 2018)

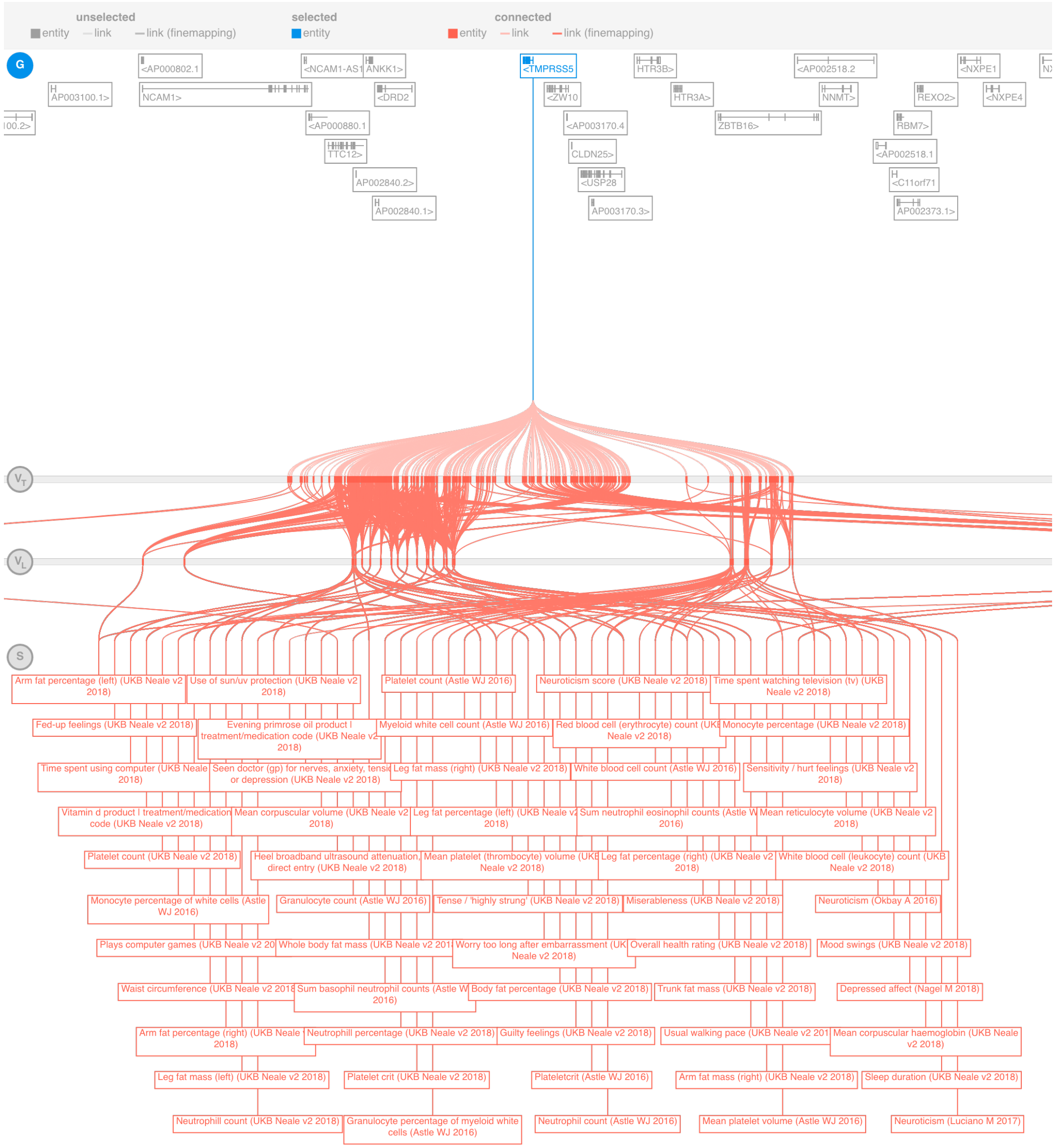
unselected
entity
link
link (finemapping)
selected
entity
connected
entity
link
link (finemapping)
G
<AP000802.1
<NCAM1-AS1
ANKK1>
<TMPRSS5
HTR3B>
<AP002518.2
<NXPE1
AP003100.1>
NCAM1>
<DRD2
<ZW10
HTR3A>
NNMT>
REXO2>
<NXPE4
<AP000880.1
<AP003170.4
ZBTB16>
RBM7>
TTC12>
CLDN25>
<AP002518.1
AP002840.2>
<USP28
<C11orf71
AP002840.1>
AP003170.3>
AP002373.1>
S
Arm fat percentage (left) (UKB Neale v2 2018)
Use of sun/uv protection (UKB Neale v2 2018)
Platelet count (Astle WJ 2016)
Neuroticism score (UKB Neale v2 2018)
Time spent watching television (tv) (UKB Neale v2 2018)
Fed-up feelings (UKB Neale v2 2018)
Evening primrose oil product | treatment/medication code (UKB Neale v2 2018)
Myeloid white cell count (Astle WJ 2016)
Red blood cell (erythrocyte) count (UKB Neale v2 2018)
Monocyte percentage (UKB Neale v2 2018)
Time spent using computer (UKB Neale v2 2018)
Seen doctor (gp) for nerves, anxiety, tension or depression (UKB Neale v2 2018)
Leg fat mass (right) (UKB Neale v2 2018)
White blood cell count (Astle WJ 2016)
Sensitivity / hurt feelings (UKB Neale v2 2018)
Vitamin d product | treatment/medication code (UKB Neale v2 2018)
Mean corpuscular volume (UKB Neale v2 2018)
Leg fat percentage (left) (UKB Neale v2 2018)
Sum neutrophil eosinophil counts (Astle WJ 2016)
Mean reticulocyte volume (UKB Neale v2 2018)
Platelet count (UKB Neale v2 2018)
Heel broadband ultrasound attenuation, direct entry (UKB Neale v2 2018)
Mean platelet (thrombocyte) volume (UKB Neale v2 2018)
Leg fat percentage (right) (UKB Neale v2 2018)
White blood cell (leukocyte) count (UKB Neale v2 2018)
Monocyte percentage of white cells (Astle WJ 2016)
Granulocyte count (Astle WJ 2016)
Tense / 'highly strung' (UKB Neale v2 2018)
Miserableness (UKB Neale v2 2018)
Neuroticism (Okbay A 2016)
Plays computer games (UKB Neale v2 2018)
Whole body fat mass (UKB Neale v2 2018)
Worry too long after embarrassment (UKB Neale v2 2018)
Overall health rating (UKB Neale v2 2018)
Mood swings (UKB Neale v2 2018)
Waist circumference (UKB Neale v2 2018)
Sum basophil neutrophil counts (Astle WJ 2016)
Body fat percentage (UKB Neale v2 2018)
Trunk fat mass (UKB Neale v2 2018)
Depressed affect (Nagel M 2018)
Arm fat percentage (right) (UKB Neale v2 2018)
Neutrophill percentage (UKB Neale v2 2018)
Guilty feelings (UKB Neale v2 2018)
Usual walking pace (UKB Neale v2 2018)
Mean corpuscular haemoglobin (UKB Neale v2 2018)
Leg fat mass (left) (UKB Neale v2 2018)
Platelet crit (UKB Neale v2 2018)
Plateletcrit (Astle WJ 2016)
Arm fat mass (right) (UKB Neale v2 2018)
Sleep duration (UKB Neale v2 2018)
Neutrophill count (UKB Neale v2 2018)
Granulocyte percentage of myeloid white cells (Astle WJ 2016)
Neutrophil count (Astle WJ 2016)
Mean platelet volume (Astle WJ 2016)
Neuroticism (Luciano M 2017)

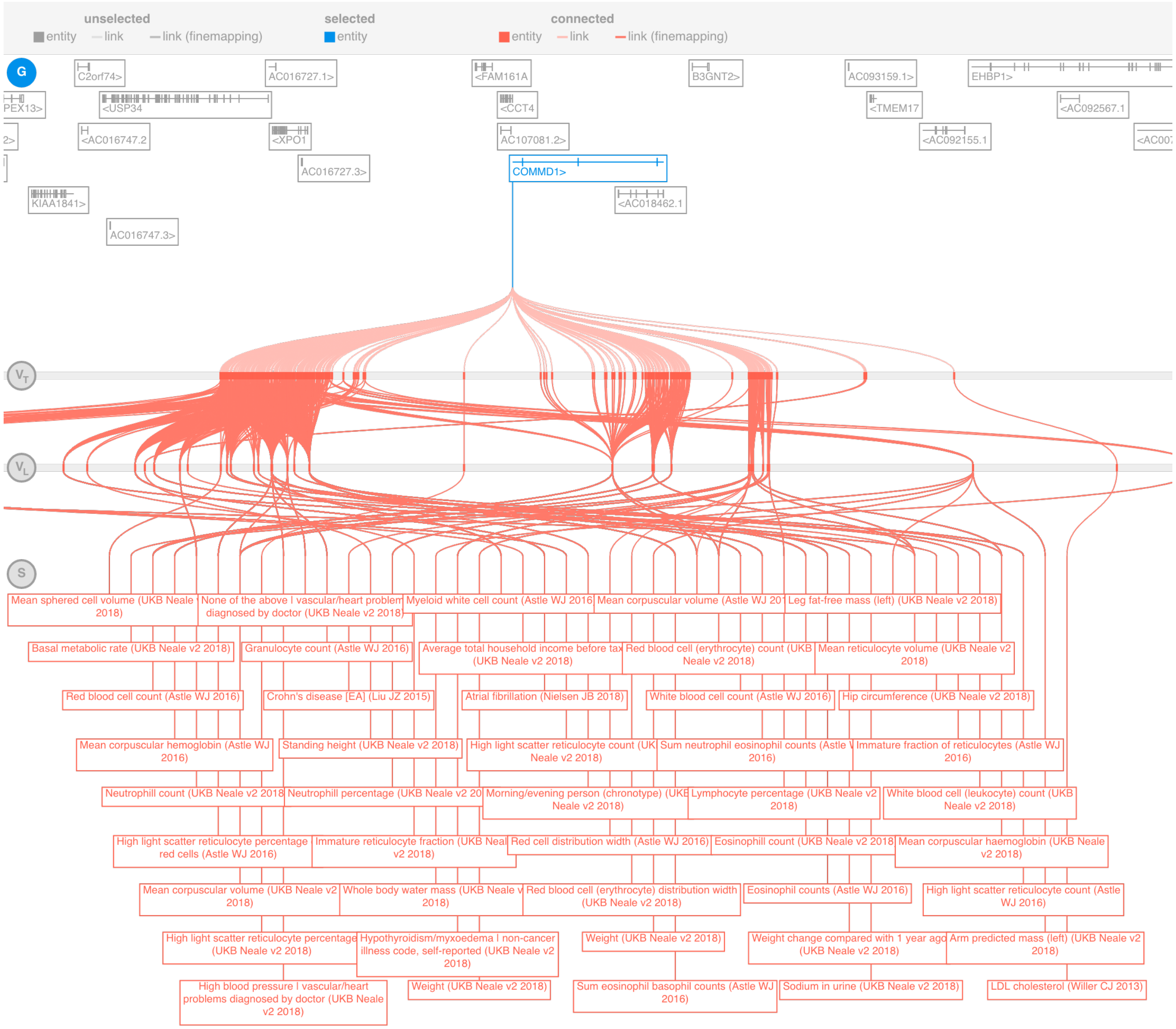
unselected
entity
link
link (finemapping)
selected
entity
connected
entity
link
link (finemapping)
COMMD1>
Mean sphered cell volume (UKB Neale v2 2018)
None of the above | vascular/heart problems diagnosed by doctor (UKB Neale v2 2018)
Myeloid white cell count (Astle WJ 2016)
Leg fat-free mass (left) (UKB Neale v2 2018)
Basal metabolic rate (UKB Neale v2 2018)
Granulocyte count (Astle WJ 2016)
Mean reticulocyte volume (UKB Neale v2 2018)
Red blood cell count (Astle WJ 2016)
Crohn's disease [EA] (Liu JZ 2015)
Atrial fibrillation (Nielsen JB 2018)
White blood cell count (Astle WJ 2016)
Hip circumference (UKB Neale v2 2018)
Standing height (UKB Neale v2 2018)
Immature fraction of reticulocytes (Astle WJ 2016)
Lymphocyte percentage (UKB Neale v2 2018)
White blood cell (leukocyte) count (UKB Neale v2 2018)
Red cell distribution width (Astle WJ 2016)
Eosinophill count (UKB Neale v2 2018)
Mean corpuscular haemoglobin (UKB Neale v2 2018)
Eosinophil counts (Astle WJ 2016)
High light scatter reticulocyte count (Astle WJ 2016)
Weight (UKB Neale v2 2018)
Arm predicted mass (left) (UKB Neale v2 2018)
Weight (UKB Neale v2 2018)
Sodium in urine (UKB Neale v2 2018)
LDL cholesterol (Willer CJ 2013)

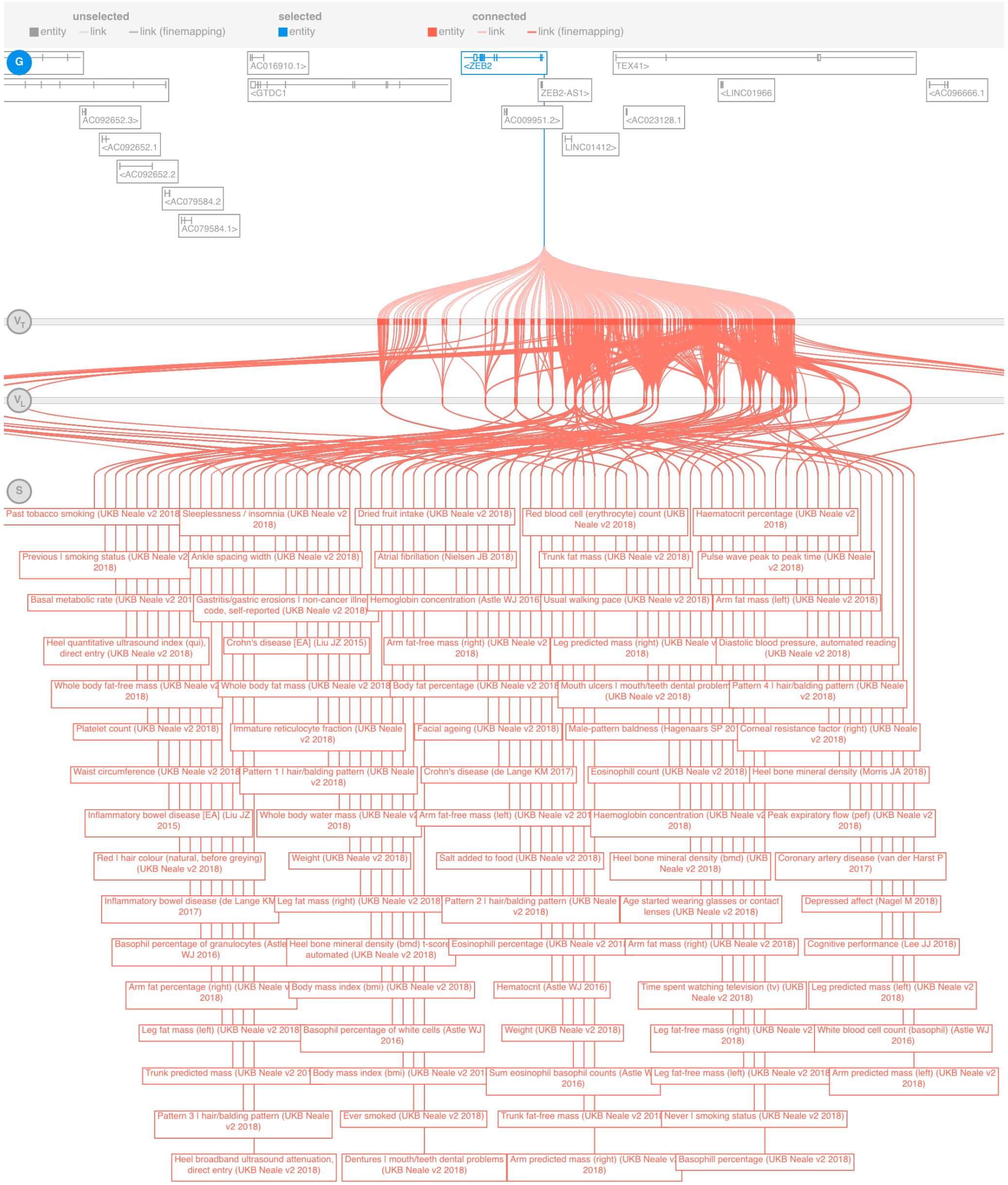
unselected
entity
link
link (finemapping)
selected
entity
connected
entity
link
link (finemapping)
G
AC016910.1>
<ZEB2
TEX41>
<GTDC1
ZEB2-AS1>
<LINC01966
<AC096666.1
AC092652.3>
AC009951.2>
<AC023128.1
<AC092652.1
LINC01412>
<AC092652.2
<AC079584.2
AC079584.1>
S
Past tobacco smoking (UKB Neale v2 2018)
Sleeplessness / insomnia (UKB Neale v2 2018)
Dried fruit intake (UKB Neale v2 2018)
Red blood cell (erythrocyte) count (UKB Neale v2 2018)
Haematocrit percentage (UKB Neale v2 2018)
Previous I smoking status (UKB Neale v2 2018)
Ankle spacing width (UKB Neale v2 2018)
Atrial fibrillation (Nielsen JB 2018)
Trunk fat mass (UKB Neale v2 2018)
Pulse wave peak to peak time (UKB Neale v2 2018)
Basal metabolic rate (UKB Neale v2 201
Gastritis/gastric erosions I non-cancer illne code, self-reported (UKB Neale v2 2018)
Hemoglobin concentration (Astle WJ 2016)
Usual walking pace (UKB Neale v2 2018)
Arm fat mass (left) (UKB Neale v2 2018)
Heel quantitative ultrasound index (qui), direct entry (UKB Neale v2 2018)
Crohn's disease [EA] (Liu JZ 2015)
Arm fat-free mass (right) (UKB Neale v2 2018)
Leg predicted mass (right) (UKB Neale v
Diastolic blood pressure, automated reading (UKB Neale v2 2018)
Whole body fat-free mass (UKB Neale v2 2018)
Whole body fat mass (UKB Neale v2 2018)
Body fat percentage (UKB Neale v2 2018)
Mouth ulcers I mouth/teeth dental problem (UKB Neale v2 2018)
Pattern 4 I hair/balding pattern (UKB Neale v2 2018)
Platelet count (UKB Neale v2 2018)
Immature reticulocyte fraction (UKB Neale v2 2018)
Facial ageing (UKB Neale v2 2018)
Male-pattern baldness (Hagenaars SP 20
Corneal resistance factor (right) (UKB Neale v2 2018)
Waist circumference (UKB Neale v2 2018)
Pattern 1 I hair/balding pattern (UKB Neale v2 2018)
Crohn's disease (de Lange KM 2017)
Eosinophill count (UKB Neale v2 2018)
Heel bone mineral density (Morris JA 2018)
Inflammatory bowel disease [EA] (Liu JZ 2015)
Whole body water mass (UKB Neale v2 2018)
Arm fat-free mass (left) (UKB Neale v2 201
Haemoglobin concentration (UKB Neale v2 2018)
Peak expiratory flow (pef) (UKB Neale v2 2018)
Red I hair colour (natural, before greying) (UKB Neale v2 2018)
Weight (UKB Neale v2 2018)
Salt added to food (UKB Neale v2 2018)
Heel bone mineral density (bmd) (UKB Neale v2 2018)
Coronary artery disease (van der Harst P 2017)
Inflammatory bowel disease (de Lange KM 2017)
Leg fat mass (right) (UKB Neale v2 2018)
Pattern 2 I hair/balding pattern (UKB Neale v2 2018)
Age started wearing glasses or contact lenses (UKB Neale v2 2018)
Depressed affect (Nagel M 2018)
Basophil percentage of granulocytes (Astle WJ 2016)
Heel bone mineral density (bmd) t-score automated (UKB Neale v2 2018)
Eosinophill percentage (UKB Neale v2 2018)
Arm fat mass (right) (UKB Neale v2 2018)
Cognitive performance (Lee JJ 2018)
Arm fat percentage (right) (UKB Neale v2 2018)
Body mass index (bmi) (UKB Neale v2 2018)
Hematocrit (Astle WJ 2016)
Time spent watching television (tv) (UKB Neale v2 2018)
Leg predicted mass (left) (UKB Neale v2 2018)
Leg fat mass (left) (UKB Neale v2 2018)
Basophil percentage of white cells (Astle WJ 2016)
Weight (UKB Neale v2 2018)
Leg fat-free mass (right) (UKB Neale v2 2018)
White blood cell count (basophil) (Astle WJ 2016)
Trunk predicted mass (UKB Neale v2 201
Body mass index (bmi) (UKB Neale v2 201
Sum eosinophil basophil counts (Astle W 2016)
Leg fat-free mass (left) (UKB Neale v2 2018)
Arm predicted mass (left) (UKB Neale v2 2018)
Pattern 3 I hair/balding pattern (UKB Neale v2 2018)
Ever smoked (UKB Neale v2 2018)
Trunk fat-free mass (UKB Neale v2 2018)
Never I smoking status (UKB Neale v2 2018)
Heel broadband ultrasound attenuation, direct entry (UKB Neale v2 2018)
Dentures I mouth/teeth dental problems (UKB Neale v2 2018)
Arm predicted mass (right) (UKB Neale v2 2018)
Basophill percentage (UKB Neale v2 2018)

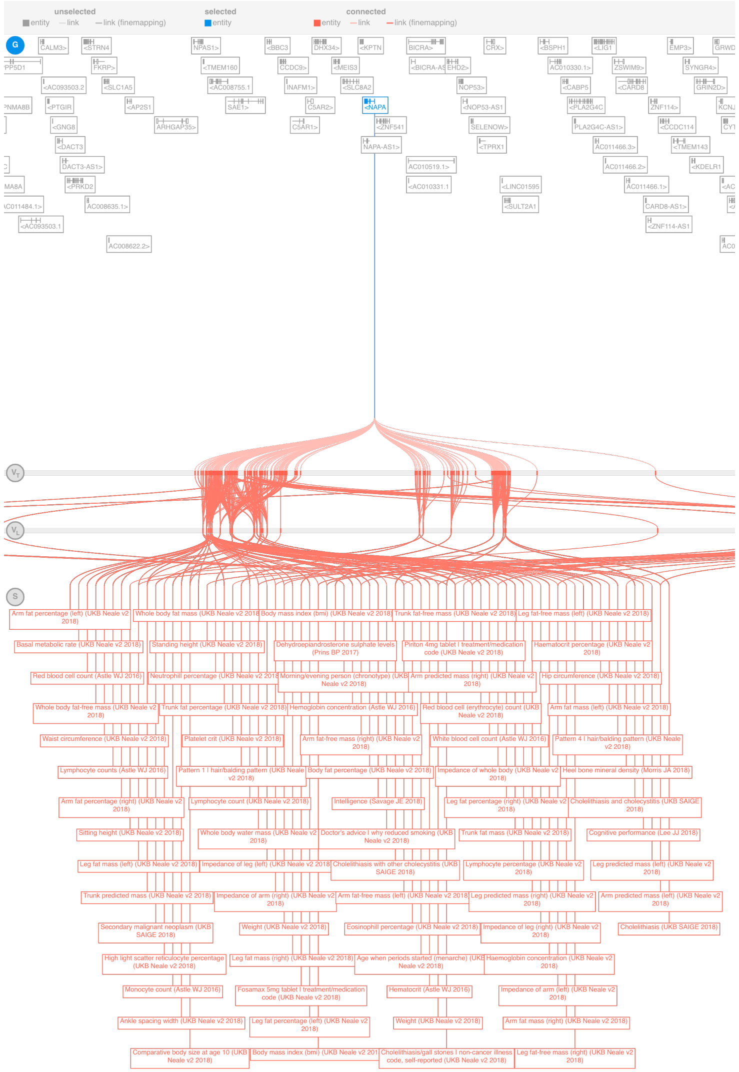
unselected
entity — link — link (finemapping)
selected
entity
connected
entity — link — link (finemapping)
G
CALM3>
<STRN4
FKRP>
<AC093503.2
<SLC1A5
<PTGIR
<AP2S1
<GNG8
ARHGAP35>
<DACT3
DACT3-AS1>
<PRKD2
AC008635.1>
<AC093503.1
AC008622.2>
NPAS1>
<TMEM160
<AC008755.1
SAE1>
<BBC3
CCDC9>
INAFM1>
C5AR2>
C5AR1>
DHX34>
<MEIS3
<SLC8A2
<KPTN
<NAPA
<ZNF541
NAPA-AS1>
BICRA>
<BICRA-AS
EHD2>
NOP53>
<NOP53-AS1
SELENOW>
<TPRX1
AC010519.1>
<AC010331.1
CRX>
<LINC01595
<SULT2A1
<BSPH1
AC010330.1>
<CABP5
<PLA2G4C
PLA2G4C-AS1>
AC011466.3>
AC011466.2>
AC011466.1>
<LIG1
ZSWIM9>
<CARD8
ZNF114>
<CCDC114
<TMEM143
CARD8-AS1>
<ZNF114-AS1
EMP3>
SYNGR4>
GRIN2D>
<KDELR1
V
V
S
Arm fat percentage (left) (UKB Neale v2 2018)
Whole body fat mass (UKB Neale v2 2018)
Body mass index (bmi) (UKB Neale v2 2018)
Trunk fat-free mass (UKB Neale v2 2018)
Leg fat-free mass (left) (UKB Neale v2 2018)
Basal metabolic rate (UKB Neale v2 2018)
Standing height (UKB Neale v2 2018)
Dehydroepiandrosterone sulphate levels (Prins BP 2017)
Piriton 4mg tablet | treatment/medication code (UKB Neale v2 2018)
Haematocrit percentage (UKB Neale v2 2018)
Red blood cell count (Astle WJ 2016)
Neutrophill percentage (UKB Neale v2 2018)
Morning/evening person (chronotype) (UKB Neale v2 2018)
Arm predicted mass (right) (UKB Neale v2 2018)
Hip circumference (UKB Neale v2 2018)
Whole body fat-free mass (UKB Neale v2 2018)
Trunk fat percentage (UKB Neale v2 2018)
Hemoglobin concentration (Astle WJ 2016)
Red blood cell (erythrocyte) count (UKB Neale v2 2018)
Arm fat mass (left) (UKB Neale v2 2018)
Waist circumference (UKB Neale v2 2018)
Platelet crit (UKB Neale v2 2018)
Arm fat-free mass (right) (UKB Neale v2 2018)
White blood cell count (Astle WJ 2016)
Pattern 4 | hair/balding pattern (UKB Neale v2 2018)
Lymphocyte counts (Astle WJ 2016)
Pattern 1 | hair/balding pattern (UKB Neale v2 2018)
Body fat percentage (UKB Neale v2 2018)
Impedance of whole body (UKB Neale v2 2018)
Heel bone mineral density (Morris JA 2018)
Arm fat percentage (right) (UKB Neale v2 2018)
Lymphocyte count (UKB Neale v2 2018)
Intelligence (Savage JE 2018)
Leg fat percentage (right) (UKB Neale v2 2018)
Cholelithiasis and cholecystitis (UKB SAIGE 2018)
Sitting height (UKB Neale v2 2018)
Whole body water mass (UKB Neale v2 2018)
Doctor's advice | why reduced smoking (UKB Neale v2 2018)
Trunk fat mass (UKB Neale v2 2018)
Cognitive performance (Lee JJ 2018)
Leg fat mass (left) (UKB Neale v2 2018)
Impedance of leg (left) (UKB Neale v2 2018)
Cholelithiasis with other cholecystitis (UKB SAIGE 2018)
Lymphocyte percentage (UKB Neale v2 2018)
Leg predicted mass (left) (UKB Neale v2 2018)
Trunk predicted mass (UKB Neale v2 2018)
Impedance of arm (right) (UKB Neale v2 2018)
Arm fat-free mass (left) (UKB Neale v2 2018)
Leg predicted mass (right) (UKB Neale v2 2018)
Arm predicted mass (left) (UKB Neale v2 2018)
Secondary malignant neoplasm (UKB SAIGE 2018)
Weight (UKB Neale v2 2018)
Eosinophil percentage (UKB Neale v2 2018)
Impedance of leg (right) (UKB Neale v2 2018)
Cholelithiasis (UKB SAIGE 2018)
High light scatter reticulocyte percentage (UKB Neale v2 2018)
Leg fat mass (right) (UKB Neale v2 2018)
Age when periods started (menarche) (UKB Neale v2 2018)
Haemoglobin concentration (UKB Neale v2 2018)
Monocyte count (Astle WJ 2016)
Fosamax 5mg tablet | treatment/medication code (UKB Neale v2 2018)
Hematocrit (Astle WJ 2016)
Impedance of arm (left) (UKB Neale v2 2018)
Ankle spacing width (UKB Neale v2 2018)
Leg fat percentage (left) (UKB Neale v2 2018)
Weight (UKB Neale v2 2018)
Arm fat mass (right) (UKB Neale v2 2018)
Comparative body size at age 10 (UKB Neale v2 2018)
Body mass index (bmi) (UKB Neale v2 2018)
Cholelithiasis/gall stones | non-cancer illness code, self-reported (UKB Neale v2 2018)
Leg fat-free mass (right) (UKB Neale v2 2018)

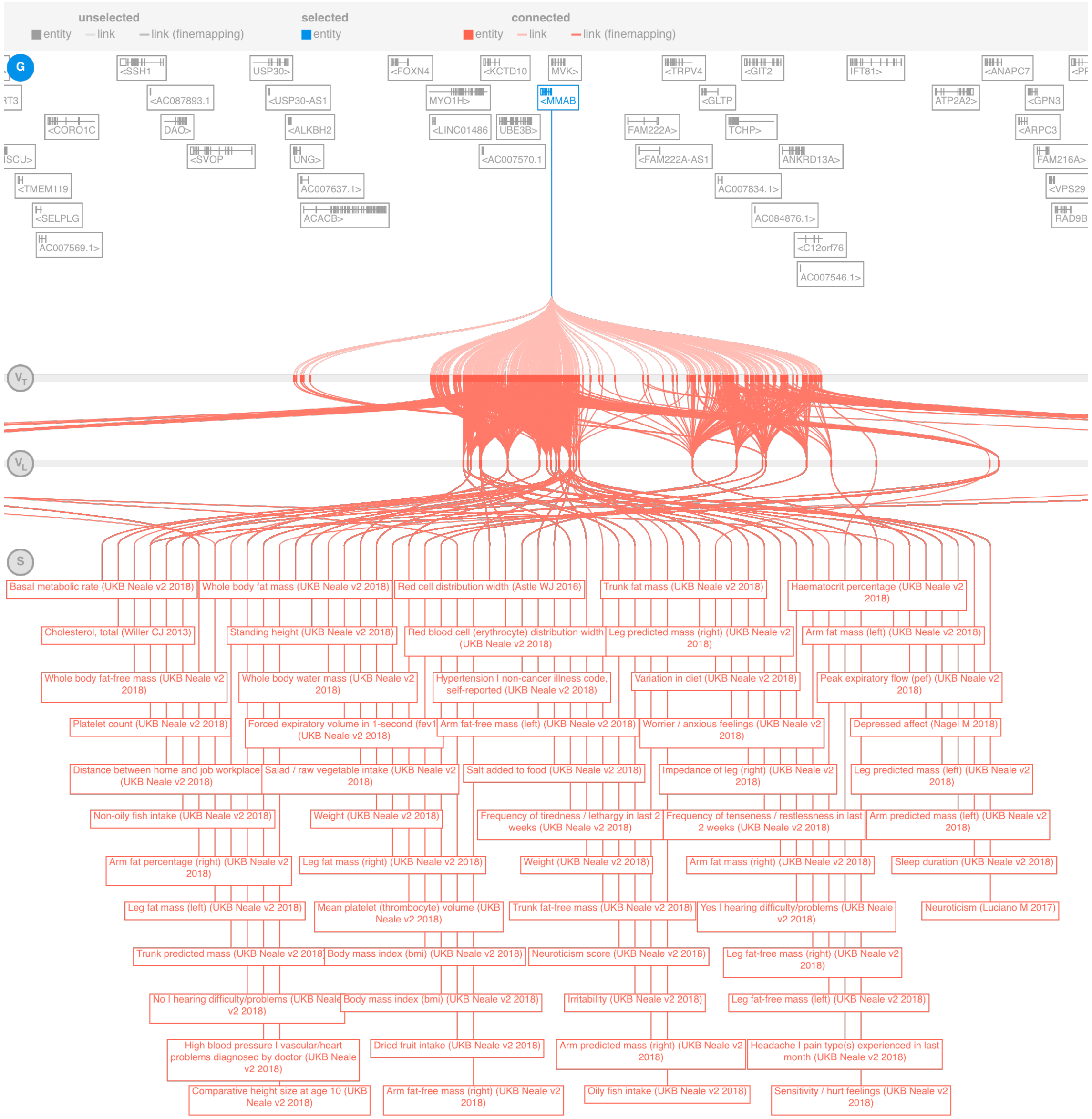

unselected
entity
link
link (finemapping)
selected
entity
connected
entity
link
link (finemapping)
G
<SSH1
USP30>
<FOXN4
<KCTD10
MVK>
<TRPV4
<GIT2
IFT81>
<ANAPC7
<AC087893.1
<USP30-AS1
MYO1H>
<MMAB
<GLTP
ATP2A2>
<GPN3
<CORO1C
DAO>
<ALKBH2
<LINC01486
UBE3B>
FAM222A>
TCHP>
<ARPC3
<SVOP
UNG>
<AC007570.1
<FAM222A-AS1
ANKRD13A>
FAM216A>
<TMEM119
AC007637.1>
AC007834.1>
<VPS29
<SELPLG
ACACB>
AC084876.1>
AC007569.1>
<C12orf76
AC007546.1>
S
Basal metabolic rate (UKB Neale v2 2018)
Whole body fat mass (UKB Neale v2 2018)
Red cell distribution width (Astle WJ 2016)
Trunk fat mass (UKB Neale v2 2018)
Haematocrit percentage (UKB Neale v2 2018)
Cholesterol, total (Willer CJ 2013)
Standing height (UKB Neale v2 2018)
Red blood cell (erythrocyte) distribution width (UKB Neale v2 2018)
Leg predicted mass (right) (UKB Neale v2 2018)
Arm fat mass (left) (UKB Neale v2 2018)
Whole body fat-free mass (UKB Neale v2 2018)
Whole body water mass (UKB Neale v2 2018)
Hypertension | non-cancer illness code, self-reported (UKB Neale v2 2018)
Variation in diet (UKB Neale v2 2018)
Peak expiratory flow (pef) (UKB Neale v2 2018)
Platelet count (UKB Neale v2 2018)
Forced expiratory volume in 1-second (fev1) (UKB Neale v2 2018)
Arm fat-free mass (left) (UKB Neale v2 2018)
Worrier / anxious feelings (UKB Neale v2 2018)
Depressed affect (Nagel M 2018)
Distance between home and job workplace (UKB Neale v2 2018)
Salad / raw vegetable intake (UKB Neale v2 2018)
Salt added to food (UKB Neale v2 2018)
Impedance of leg (right) (UKB Neale v2 2018)
Leg predicted mass (left) (UKB Neale v2 2018)
Non-oily fish intake (UKB Neale v2 2018)
Weight (UKB Neale v2 2018)
Frequency of tiredness / lethargy in last 2 weeks (UKB Neale v2 2018)
Frequency of tenseness / restlessness in last 2 weeks (UKB Neale v2 2018)
Arm predicted mass (left) (UKB Neale v2 2018)
Arm fat percentage (right) (UKB Neale v2 2018)
Leg fat mass (right) (UKB Neale v2 2018)
Weight (UKB Neale v2 2018)
Arm fat mass (right) (UKB Neale v2 2018)
Sleep duration (UKB Neale v2 2018)
Leg fat mass (left) (UKB Neale v2 2018)
Mean platelet (thrombocyte) volume (UKB Neale v2 2018)
Trunk fat-free mass (UKB Neale v2 2018)
Yes | hearing difficulty/problems (UKB Neale v2 2018)
Neuroticism (Luciano M 2017)
Trunk predicted mass (UKB Neale v2 2018)
Body mass index (bmi) (UKB Neale v2 2018)
Neuroticism score (UKB Neale v2 2018)
Leg fat-free mass (right) (UKB Neale v2 2018)
No | hearing difficulty/problems (UKB Neale v2 2018)
Body mass index (bmi) (UKB Neale v2 2018)
Irritability (UKB Neale v2 2018)
Leg fat-free mass (left) (UKB Neale v2 2018)
High blood pressure | vascular/heart problems diagnosed by doctor (UKB Neale v2 2018)
Dried fruit intake (UKB Neale v2 2018)
Arm predicted mass (right) (UKB Neale v2 2018)
Headache | pain type(s) experienced in last month (UKB Neale v2 2018)
Comparative height size at age 10 (UKB Neale v2 2018)
Arm fat-free mass (right) (UKB Neale v2 2018)
Oily fish intake (UKB Neale v2 2018)
Sensitivity / hurt feelings (UKB Neale v2 2018)

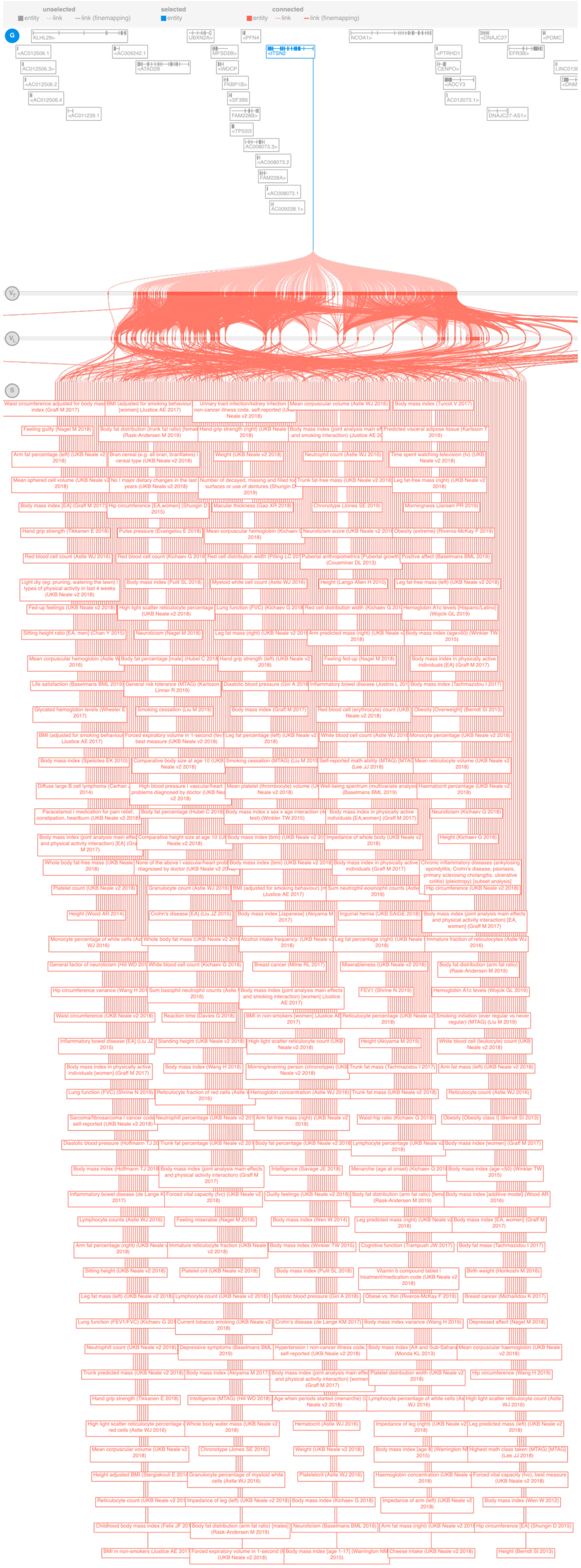

Supplementary Table 1: Clinical characteristics and cataract phenotyping of participating cohorts

Cohort	Ancestry	N of subjects	Age, yrs (Mean ± SD)	Sex (% F)	Phenotyping methods	Cataract Grading system*	Nuclear cataract grade (mean ± SD)
Discovery (n = 14,151)							
AREDS *	European	1434	67.4± 4.7	59.0	Lens photography	AREDS	2.2 ± 0.8
BMES	European	2209	66.3 ± 8.8	57.1	Lens photography	Modified Wisconsin	2.5 ± 0.7
RSI-III	European	1424	71.2 ± 6.2	54.1	Lens photography	CNDS	89.6 ± 36.9
TwinsUK	European	2285	62.2 ± 6.8	98.0	Lens photography	CNDS	62.1 ± 18.1
SCES-610	Asian (Chinese)	1704	57.5 ± 8.9	48.8	Lens photography	Modified Wisconsin	2.3 ± 0.7
SCES-OmniExpress	Asian (Chinese)	526	58.7 ± 8.5	48.3	Lens photography	Modified Wisconsin	2.4 ± 0.7
SiMES *	Asian (Malay)	2369	58.4 ± 10.9	50.6	Lens photography	Modified Wisconsin	2.5 ± 0.7
SINDI *	Asian (Indian)	2200	56.4 ± 9.1	48.3	Lens photography	Modified Wisconsin	2.3 ± 0.7
Replication (n = 5,299)		N of subjects (N of cases)					
BDES	European	1559 (879)	62.3 ± 10.5	53.7	Lens photography	Original Wisconsin	2.7 ± 0.9
BES	Asian (Chinese)	1528 (769)	57.4 ± 9.4	61.6	Lens photography	Modified AREDS	2.6 ± 1.1
INDEYE(S)	Asian (Indian)	1908 (1097)	66.7 ± 6.0	52.4	Lens photography	LOCS III	3.7 ± 1.1
SLCCS	European	304 (62)	73.9 ± 10.2	54.9	Slit-lamp biomicroscopy	LOCS III	2.4 ± 0.7

N – number of individuals studied; % F – percentage of females; SD – standard deviation; AREDS – Age-Related Eye Diseases Study; BMES – Blue Mountains Eye Study; RSI-III – Rotterdam Study I-III; TwinsUK – TwinsUK Adult Twin Registry; SCES-610 and SCES-OmniExpress – The Singapore Chinese Eye Study, genotyped by Illumina HumanHap610 and OmniExpress chips, respectively; SiMES – Singapore Malay Eye Study; SINDI – Singapore Indian Eye Study; BDES – Beaver Dam Eye Study; BES - Beijing Eye Study; INDEYE(S) – India Study of Age-related Eye Disease South India Centre; SLCCS – South London Case Control Study.
*Cataract grading system: CNDS – central nuclear dip score; Wisconsin Lens photography – Wisconsin Cataract Grading System; LOCS III – Lens Opacities Classification System III. For the association analysis, all studies adjusted for age and sex. Studies which also adjusted for genetic PCs are denoted by *.

Supplementary Table 2: Comparison between cataract grading systems used in this study

Cohort	Cataract Grading System*	Characteristics	Standards used
**Discovery**			
AREDS	AREDS	Decimal score in 0.1-unit steps, comparing to 7 standard lens photos	AREDS
BMES	Modified Wisconsin	Decimal score in 0.1-unit steps, comparing to 4 standard lens photos	Modified Wisconsin
RSI-III	CNDS	Decimal score of the pixel density within the centre of the lens nucleus	-
TwinsUK	CNDS	Decimal score of the pixel density within the centre of the lens nucleus	-
SCES-610	Modified Wisconsin	Decimal score in 0.1-unit steps, comparing to 4 standard lens photos	Modified Wisconsin
SCES-OmniExpress	Modified Wisconsin	Decimal score in 0.1-unit steps, comparing to 4 standard lens photos	Modified Wisconsin
SiMES	Modified Wisconsin	Decimal score in 0.1-unit steps, comparing to 4 standard lens photos	Modified Wisconsin
SINDI	Modified Wisconsin	Decimal score in 0.1-unit steps, comparing to 4 standard lens photos	Modified Wisconsin
**Replication**			
BDES	Original Wisconsin	Integer score, comparing to 4 standard lens photos	Original Wisconsin
BES	Modified AREDS	Integer score, comparing to 6 standard lens photos	Modified AREDS
INDEYE(S)	LOCS III	Decimal score, comparing to 6 standard lens photos	LOCS III
SLCCS	LOCS III	Integer score, comparing to 6 standard lens photos	LOCS III

AREDS – Age-Related Eye Diseases Study; BMES – Blue Mountains Eye Study; RSI-III – Rotterdam Study I-III; TwinsUK – TwinsUK Adult Twin Registry; SCES-610 and SCES-OmniExpress – The Singapore Chinese Eye Study, genotyped by Illumina HumanHap610 and OmniExpress chips, respectively; SiMES – Singapore Malay Eye Study; SINDI – Singapore Indian Eye Study; BDES – Beaver Dam Eye Study; BES - Beijing Eye Study; INDEYE(S) – India Study of Age-related Eye Disease South India Centre; SLCCS – South London Case Control Study.
*Cataract grading system: CNDS – central nuclear dip score; Wisconsin Lens photography – Wisconsin Cataract Grading System; LOCS III – Lens Opacities Classification System III.

Supplementary Table 3: Genotyping and imputation platforms in the participating cohorts

Cohort	Genotyping platform	1000G Imputation	
		**Software**	**Reference Panel**
**Discovery**			
AREDS	Illumina HumanOmni2.5-4v1_B chip array	IMPUTE2	Phase 1, ALL 2012
BMES	Illumina Human 670-Quadv1 custom	IMPUTE	Phase 1, EUR 2012
RSI-III	Illumina Infinium II HumanHap550 chip v3.0 array	Minimac	Phase 1, EUR 2012
TwinsUK	Illumina HumanHap 300kDuo & HumanHap610-Quad	IMPUTE2	Phase 3, EUR 2014
SCES-610	Illumina HumanHap610-Quad	Minimac	Phase 1, ALL 2012
SCES-OmniExpress	Illumina OmniExpress	Minimac	Phase 1, ALL 2012
SiMES	Illumina HumanHap610-Quad	Minimac	Phase 1, ALL 2012
SINDI	Illumina HumanHap610-Quad	Minimac	Phase 1, ALL 2012
**Replication**			
BDES	Illumina iSelect Custom Panel	Minimac	Phase 1, ALL 2012
BES	Illumina HumanHap610-Quad	Minimac	Phase 1, ALL 2012
	Illumina OmniExpress	IMPUTE2	Phase 3, ALL 2014
INDEYE(S)	TaqMan	-	-
SLCCS	Illumina OmniExpress Exome Array	Minimac	Phase 3, ALL 2014

Chinese Eye Study, genotyped by Illumina HumanHap610 and OmniExpress chips, respectively; SiMES – Singapore Malay Eye Study; SINDI – Singapore Indian Eye Study; BDES – Beaver Dam Eye Study; BES - Beijing Eye Study; INDEYE(S) – India Study of Age-related Eye Disease South India Centre; SLCCS – South London Case Control Study. CNDS – central nuclear dip score; Wisconsin Lens photography – Wisconsin Cataract Grading System; LOCS III – Lens Opacities Classification System III.
EUR – European ancestry; ALL – all populations in 1000 Genome project.

Supplementary Table 4: Inflation factors (λ) of the test statistics for each cohort participating in the discovery phase

Cohort	Ancestry	λ
AREDS*	European	1.003
BMES	European	1.01
RSI-III	European	0.99
TwinsUK	European	1.02
**European ancestry cohorts**		**1.02**
SCES-610	Asian (Chinese)	1.01
SCES-OmniExpress	Asian (Chinese)	1.01
SiMES*	Asian (Malay)	1.01
SINDI*	Asian (Indian)	1.06
**Asian Ancestry cohorts**		**1.03**
**All Discovery cohorts**		**1.04**

AREDS – Age-Related Eye Diseases Study; BMES – Blue Mountains Eye Study; RSI-III – Rotterdam Study I-III; TwinsUK – TwinsUK Adult Twin Registry; SCES-610 and SCES-OmniExpress – The Singapore Chinese Eye Study, genotyped by Illumina HumanHap610-Quad and OmniExpress chips, respectively; SiMES – Singapore Malay Eye Study; SINDI – Singapore Indian Eye Study. *These studies included nuclear principal components as covariates.

Supplementary Table 5: Regulatory elements data (ENCODE, HaploReg) for variants that were found associated to age-related nuclear cataract ($P<1x10^{-6}$) in the combined analysis (Discovery phase), as well as the *GJA3* and *CRYAB* loci

Chr	Lead SNP	P value	Closest Gene	H3K27ac mark	N H3K27ac marks	Enhancer histone marks	DNAse hypersensitivity sites	Binding proteins	Regulatory motifs
**21**	rs7278468	3.6E-17	*CRYAA*	No	8	IPSC, LIV, PLCNT			CACD, HDAC2, Klf4, Spz1
**3**	rs9842371	2.6E-12	*SOX2-OT*	Yes	4	12 tissues	4 tissues	GATA2	Dmbx1,Hoxa5,Otx2
**11**	rs4936279	4.2E-11	*TMPRSS5*	No	4				CTCF,E2F, ELF1, HEY1,Rad21, SMC3, Sin3Ak, YY1
**2**	rs62149908	6.5E-08	*COMMD1*	No	0	9 tissues	BLD,VAS		ERalpha-a,Zfx
**2**	rs16823886	8.8E-08	*LINC01412*	Yes	2	5 tissues			
**12**	rs11067211	1.6E-07	*MMAB*	No	5		IPSC,BLD		Pax5,Pbx3
**19**	rs1005911	2.8E-07	*GLTSCR1*	No	5				
**2**	rs61185326	3.0E-07	-	No	6				Foxj2, Foxp1, Mef2, Pou2f2, TATA
**2**	rs13021828	6.1E-07	*ITSN2*	No	0		28 tissues	CTCF, POL2, POL24H8, SRF, STAT3	ERalpha-a,ZBRK1
**13**	rs17077135	6.2E-06	*GJA3*	No	2		BRN		GR, Sox
**11**	rs10789852	8.4E-06	*HSPB2/C11orf 52 (CRYAB)*	No	10	7 tissues			BDP1,LUN-1

Only the most strongly associated variant per locus is reported; Chr – chromosome; SNP – SNP rs number; P value – P-value from the combined analysis (Phase III); H3K27ac mark – whether a variant falls within a H3K27ac mark (a mark of active regulatory elements); N – H3K27ac marks – number of H3K27ac mark within a 50kb window around the studied variant.

Supplementary Table 6: Publicly available expression data in human and mice for the genes closest to or in the same locus as the SNPs associated with age-related nuclear cataract at P<1x10-6 in the combined analysis (Discovery phase), as well as the *GJA3* and *CRYAB* loci

				iSyTE		Ocular tissue database		MGI GXD	
**Chr**	**Lead SNP**	**P**	**Closest Gene**	**Lens enrichment**	**Enrichment for other genes in the locus**	**PLIER score**	**PLIER scores for other genes in the locus**	**Lens expression**	**Other genes in the locus**
**21**	rs7278468	3.60E-17	*CRYAA*	*Yes*	*No*	12800.8	*UTAF1:* 119.4	*Yes*	*No*
**3**	rs9842371	2.60E-12	*SOX2-OT*	*No**	*SOX2*	289.7	*SOX2:* 289.7; *FXR1*: 700.1; *DNAJC19*: 118.4	*No*	*No*
**11**	rs4936279	4.20E-11	*TMPRSS5*	*Yes*	*No*	14.1	*ZW10:* 32.0; *USP28*: 38.9; HTR3B: 14.6; HTR3A: 24.2; ZBTB16: 41.8	*No*	*No*
**2**	rs62149908	6.50E-08	*COMMD1*	*No*	*No*	192.9	*CCT4*: 292.4; *B3GNT2*: 56.7	*No*	*No*
**2**	rs16823886	8.80E-08	*LINC01412*	*No*	*ZEB2*	-	*ZEB2:* 160.5	*No*	*No*
**12**	rs11067211	1.60E-07	*MMAB*	*No*	*No*	76.1	*FOXN4*: 23.0; *MYO1H*: 19.6; *KCTD10*: 50.3; *UBE3B*: 78.9; *MVK*: 31.5	*No*	*No*
**19**	rs1005911	2.80E-07	*GLTSCR1*	*No*	*CRX*	55.6	*KPTN:* 20.7; *SEPW1*: 164.1; *GLTSCR1*: 69.8; *CRX*: 14.3; *NAPA*: 45.0; *ZNF451*: 92.0; *SNORD23*: 69.8; *TPRX1*: 18.0	*No*	*No*
**2**	rs61185326	3.00E-07	-	*No*	*No*	-	-	*No*	*No*
**2**	rs13021828	6.10E-07	*ITSN2*	*No*	*No*	107.6	*KLHL29*: 23.7; *ATAD2B*: 6.9; *UBXN2A*: 170.7; *PFN4*: 26.4; *MFSD2B*: 130.0; *TP53I3*: 183:3; *FKBP1B*: 130.0; *SF3B14*: 138.5	*No*	*No*
**13**	rs17077135	6.20E-06	*GJA3*	*Yes*	*No*	74.6	-	*Yes*	*No*
**11**	rs10789852	8.40E-06	*CRYAB*	*Yes*	*HSPB2, C11orf52, ALG9*	2860.4	*HSPB2:* 273.6; *C11orf5:* 275.6; *ALG9:* 30.1; *FDXACB1*: 30.1; *DIXDC1*: 188.6; *PIH1D2*: 9.0; *DLAT*: 68.3; *TIMM8B*: 431.7; *SDHD*: 9.0; *PPP2R1B*: 63.0	*Yes*	*No*

Chr – chromosome; SNP – SNP rs number; P – P-value from the combined analysis (Discovery phase); Locus – a region between two recombination hot-spots (recombination rates ≥ 40) or a 1MB window, whichever was smaller; iSyTE – integrated Systems Tool for Eye gene discovery (http://bioinformatics.udel.edu/Research/iSyTE); Ocular tissue database (https://genome.uiowa.edu/otdb/); PLIER score – score from the probe logarithmic intensity error algorithm; MGI GXD – Mouse Genome Informatics Gene Expression Database (http://Supplemental.informatics.jax.org/expression.shtml); *expressed in embrionic lens

Supplementary Table 7: Results from the conditional analysis (GCTA) of the loci that were associated with nuclear cataract after replication

CHR	meta-analysis of GWAS sentinel SNP	P	LD panel	SNPs with Independent effect (conditional P)
2	rs62149908 (2:62191878)	6.5E-08	All	rs62149908 (3.3E-07)
			EUR	rs62149908 (1.0E-05)
			EAS	rs62149908 (1.0E-05)
			SAS	NI
	rs16823886 (2:145341259)	8.8E-08	All	rs16823886 (4.5E-07)
			EUR	rs16823886 (4.5E-07)
			EAS	rs16823886 (4.5E-07)
			SAS	rs16823886 (1.2E-08)
3	rs9842371 (3:181346937)	2.6E-12	All	rs9842371 (1.1E-12)
			EUR	rs9842371 (1.1E-12)
			EAS	rs9842371 (1.1E-12)
			SAS	rs9842371 (1.1E-12)
11	rs4936279 (11:113566207)	4.2E-11	All	rs4430548 (5.4e-09)
			EUR	rs4430548 (5.4e-09)
			EAS	rs4430548 (5.4e-09)
			SAS	rs4430548 (5.4e-09)
12	rs11067211 (12:109988214)	1.6E-07	All	rs11067211 (3.8E-07)
			EUR	rs11067211 (3.8E-07)
			EAS	rs11067211 (3.8E-07)
			SAS	rs11067211 (3.8E-07)
19	rs1005911 (19:48206092)	2.8E-07	All	rs1005911 (6.9E-07)
			EUR	rs1005911 (6.9E-07)
			EAS	rs1005911 (6.9E-07)
			SAS	rs1005911 (6.9E-07)
21	rs7278468 (21:44588757)	3.6E-17	All	rs7278468 (7.3E-28)
			EUR	rs7278468 (1.6E-15)
			EAS	rs7278468 (1.6E-15)
			SAS	rs7278468 (1.6E-15)

Legend: CHR – chromosome; sentinel SNP – most strongly associated SNP in the meta-analysis discovery phase. LD panel – 1000Genomes reference panel used for LD calculations: All – all cohorts; EUR –European ancestry individuals; EAS – East Asian ancestry individuals; SAS – south Asian ancestry. NI – no independent effects were detected.

Supplementary Table 8: Gene-based analysis based on the Discovery phase

Gene	P
CRYAA	2.9E-11
SOX2-OT	9.6E-06
TMPRSS5	1.4E-05
LINC01412	0.02
LOC440704	0.02
MMAB	0.02
COMMD1	0.02
STAB2	0.04
ITSN2	0.04

The table shows the results from the gene-based analysis (GATES) of the summary statistics from the Discovery phase (combined analysis). P value presented is corrected for multiple testing.

Supplementary Table 9: Gene set enrichment analysis results for the results obtained from the meta-analysis in the discovery stage

Pathway database: Pathway name	P- value
REACTOME: cholesterol biosynthesis	1.14E-02
KEGG: terpenoid backbone biosynthesis	1.18E-02
KEGG: folate biosynthesis	1.21E-02
REACTOME: DNA replication	1.52E-02
REACTOME: mitotic MMG1 phases	1.54E-02
REACTOME: mitotic prometaphase	1.59E-02
REACTOME: ligand gated ion channel transport	3.05E-02
KEGG: porphyrin and chlorophyll metabolism	3.09E-02
BIOCARTA: agrin in postsynaptic differentiation	3.38E-02
REACTOME: ion channel transport	3.78E-02
BIOCARTA: myocyte enhancer factor 2D	3.79E-02
REACTOME: platelet adhesion to exposed collagen	3.84E-02
REACTOME: mitotic cell cycle	3.84E-02
KEGG: glycosaminoglycan biosynthesis keratin sulfate	4.61E-02
REACTOME: keratin sulphate biosynthesis	4.61E-02

The p-values have been calculated using permutation (N=1000). Pathway databases as follows REACTOME (https://reactome.org/); KEGG – Kyoto Encyclopedia of Genes and Genomes (https://Supplemental.genome.jp/kegg/); BIOCARTA (https://Supplemental.ebi.ac.uk/miriam/main/collections/MIR:00000421)

Supplementary Note

1. Cohorts Description

The Age-Related Eye Disease Study (AREDS). AREDS was originally designed as a long-term multicenter, prospective study of the clinical course of age-related macular degeneration (AMD) and age-related cataract[1,2]. In clinical trial of high-dose vitamin and mineral supplements for AMD and a clinical trial of high-dose vitamin supplements for cataract. AREDS participants were 55 to 80 years of age at enrollment and had to be free of any illness or condition that would make long-term follow-up or compliance with study medications unlikely or difficult. On the basis of fundus photographs graded by a central

reading center, best-corrected visual acuity and ophthalmologic evaluations, 4,757 participants were enrolled.

All AREDS participants were genotyped at the Center for Inherited Disease Research. For AREDS1a-1b three chips were used for this genotyping: Affymetrix 100K, Illumina 100K and Illumina 300K. SNPs were abstracted from each of the chips and genotypes on more than one chip were checked to ensure that the calls were the same. Individuals not of European descent were removed. For AREDS 1c, genotyping of SNPs was performed using the Illumina HumanOmni2.5-4v1_B chip array. For all studies, samples with low call rate (<98%), with low mean confidence scores over all non-missing genotypes, with chromosome anomalies, or with sex-mismatch were excluded. No samples exhibited excess heterozygosity rates (1.5 interquartile ranges above or below the upper/lower quartile ranges). Cryptic relatedness was detected by estimating IBD sharing and kinship coefficients among all possible pairs and one member of each pair exhibiting a sibling or closer relationship was dropped from the analysis. SNPs were dropped from the analysis if they exhibited more than 1 blind duplicate error, more than 1 HapMap control error or more than 1 error in HapMap control trios, a genotype call rate < 99%, minor allele frequency < 0.01, or Hardy-Weinberg P value < 10^{-4}. Tests for batch effects were not significant. No sex-specific differences in allelic frequency (>0.2) or heterozygosity (>0.3) were detected.

The Beaver Dam Eye Study (BDES). BDES is a population based study of age related ocular disorders. A private census was conducted to identify all people between the ages of 43 and 84 years who were residents of the city or township of Beaver Dam, WI, USA, in 1987-8[3]. Of the 5925 eligible people identified, 4926 (83.1%) were examined during the baseline examination (1988-90), 225 (3.8%) died before examination, 91 (1.5%) had moved out of the area, 23 (0.4%) could not be located, 269 (4.5%) completed a questionnaire only, and 391 (6.6%) refused to participate.

The Beijing Eye Study (BES). BES is a population-based cross-sectional study of Chinese aged 40+ years residing in the village area of Yufa in Daxing District, south of Beijing, and in the Haidian urban district, north of Central Beijing. At the time of the survey in 2001, a total of 5,324 individuals were eligible to participate in the study, of which 4,439 individuals (83.4% response rate) were recruited. In 2006, all participants from the survey in 2001

were re-invited and 3,251 participants (73.2% response rate) were recruited, and blood was taken from 2,929 (90.1%)[4].

In BES, the degree of nuclear cataract was assessed in 6 grades using the grading system of the Age-Related Eye Disease Study (AREDS), which is an extension of the Wisconsin System[5]. Digital photographs of the lens were obtained using the slit lamp. A single grader under the supervision of senior ophthalmologists and researchers performed the lens grading[6]. The nuclear cataract grading from the 2006 follow-up study was used.

The Blue Mountains Eye Study (BMES). BMES is a population-based survey of vision and common eye diseases in the Blue Mountains, west of Sydney, Australia. Following a door-to-door census of the region, all permanent residents aged 49 years or older were invited to take a detailed eye examination. From a total of 4,433 eligible residents, 3,654 (82.4%) attended the baseline eye examination and interview between 1992 and 1994. During 1997-99 (BMES II A), 2,335 participants (75.1% of survivors) returned for examinations after 5 years. During 1999-2000, 1,174 (85.2%) new participants took part in an Extension Study of the BMES (BMES IIB).

Participants of the BMES were genotyped using the Illumina Human 670-Quadv1 custom genotyping array at the Wellcome Trust Sanger Institute, Cambridge as part of WTCCC2, and 2,761 had genotyping data available. Genotyped variants passed standard quality control.

The India Eye Study (INDEYE). The INDEYE study is a population-based study of people aged 60 years and older. The INDEYE study is aimed at estimating the age- and sex-specific prevalence of early and late AMD and of lens opacities, and at investigating associations of these conditions with tobacco use, exposure to biomass cooking fuels, outdoor work, and dietary factors. The study took place in two locations: the Gurgaon district, in Haryana state, North India, and the Pondicherry union territory and Cuddalore district in Tamil Nadu, South India. These areas were chosen to represent a mix of rural and urban populations served by the participating eye hospitals (Dr. Rajendra Prasad Centre [RPC]), Delhi; the All India Institute of Medical Sciences, Delhi; and the Aravind Eye Hospital [AEH], Pondicherry). A total of 59 clusters, 29 in North India, and 30 in South India were randomly selected on the basis that 8% of the total population would be aged 60 years and older. Before the start of the study, meetings were held with local village leaders to explain the study objectives and methods. A total of 7,518 people 60 years of

age and older (3586 in North India and 3932 in South India), were identified from enumeration and invited to take part in the study. Recruitment into the study was performed between 2005 and 2007. Informed written consent was obtained from all participants before enrollment. Information was read to people who were illiterate in the presence of a local witness, and a thumb impression of the participant signified assent. The study complied with the guidelines in the Declaration of Helsinki, and ethics approval was received from the Research Ethics Committees of the All India Institute of Medical Sciences, Aravind Eye Hospital, London School of Hygiene and Tropical Medicine, Queens University Belfast, and the Indian Council for Medical Research. In the current study only samples from the South India were used.

The Rotterdam Study (RS) is a prospective population based cohort study in the elderly living in Ommoord, a suburb of Rotterdam, the Netherlands. The Rotterdam Study consists of 3 independent cohorts: RS-I, RS-II, and RS-III. Participants underwent multiple physical examinations with regular intervals from 1991 to present. Individuals who took part in RS-I (part III), referred here as RSI-III, had lens measurements performed.

DNA was extracted from blood leucocytes according to standard procedures. Genotyping of SNPs was performed using the Human 610 Quad Arrays Illumina (RS-III). Samples with low call rate (<97.5%), with excess autosomal heterozygosity (>0.336), or with sex-mismatch were excluded, as were outliers identified by the identity-by-state clustering analysis (outliers were defined as being >3 s.d. from population mean or having identity-by-state probabilities >97%). A set of genotyped input SNPs with call rate >98%, with minor allele frequency >0.01, and with Hardy-Weinberg P value $>10^{-6}$ was used for imputation.

Singapore Cohorts (SCES, SiMES, SINDI)

Detailed information for the Singapore cohorts is provided below. For the 3 cohorts in Singapore, ethics approval was obtained from the SingHealth Centralised Institutional Review Board. All study participants were provided with written informed consent in adherence to the Declaration of Helsinki.

In the Singapore cohorts, the severity of nuclear cataract was assessed using the Wisconsin Cataract Grading System (Wisconsin System)[7], based on lens photographs and followed a decimalized system (decimal scores from 0.1 to 5.0). In brief, lens photographs were taken using a digital slit-lamp camera (model DC-1 with DF-21 flash

attachment; Topcon, Tokyo, Japan) and grading was performed through comparison with four standard photographs at the University of Sydney by a single experienced grader (AGT), with adjudication by a senior ophthalmologist (PM) and a senior researcher (JJW)[8].

The Singapore Chinese Eye Study (SCES-610 and SCES-OmniExpress). SCES is a population-based cross-sectional epidemiological study on eye diseases for Chinese aged between 40 and 80+ years old residing in Singapore. Using age-stratified random sampling strategy, 4,605 ethnic Chinese residents in the southwestern part of Singapore were eligible from the sampling frame (n = 6,752), of which 3,353 (72.8% response rate) participants were recruited between February 2009 and December 2012[9].

Genome-wide genotyping was conducted using Illumina Human-610 quad and OmniExpress Chips. The same QC methods used for SiMES and SINDI were applied to the SCES genotyping samples: samples were excluded if they showed evidence of admixture, cryptic relatedness, high heterogeneity and gender discrepancies.

The Singapore Malay Eye Study (SiMES). SiMES is a population-based cross-sectional epidemiological study on eye diseases for Malays aged between 40 and 80 years old residing in Singapore. Details of the SiMES design, sampling plan and methodology have been reported elsewhere[10]. In brief, between August 2004 and June 2006, a total of 4,168 Malay residents in the south-western part of Singapore were identified through age-stratified random sampling and were invited to participate in the study, of which 3,280 (78.7% response rate) underwent a detailed ocular examination.

Genome-wide genotyping was performed in 3,072 individuals. DNA samples were genotyped using the Illumina Human 610 Quad Beadchips[11,12]. Using the same quality control criteria, we omitted a total of 530 individuals including those of subpopulation structure (n=170), cryptic relatedness (n=279), excessive heterozygosity or high missingness rate > 5% (n=37), and gender discrepancy (n=44). After the removal of the samples, SNP QC was then applied on a total of 579,999 autosomal SNPs for the 2,542 post-QC samples. SNPs were excluded based on (i) high rates of missingness (> 5%); (ii) monomorphism or MAF < 1%; or (iii) genotype frequencies deviated from HWE ($p < 1 \times 10^{-6}$).

The Singapore Indian Eye Study (SINDI). Similar to SiMES, SINDI is a population-based cross-sectional epidemiological study on eye diseases for ethnic Indians aged between

40 and 80+ years old residing in Singapore. The study was conducted between March 2007 and December 2009. Using age-stratified random sampling, 4,497 ethnic Indian residents in the south-western part of Singapore were eligible from the sampling frame (n = 6,350), of which 3,400 participants (75.6% response rate) were recruited[9].

We performed genome-wide genotyping in 2,953 SINDI subjects. We excluded 415 subjects from the total of 2,953 genotyped samples based on: excessive heterozygosity or high missingness rate > 5% (n=34), cryptic relatedness (n=326), issues with population structure ascertainment (n=39) and gender discrepancies (n=16). This left a total of 2,538 individuals with 579,999 autosomal SNPs. During the SNP QC procedure, SNPs were excluded based on (i) high rates of missingness (> 5%); (ii) monomorphism or MAF < 1%; or (iii) genotype frequencies deviated from HWE ($p < 1 \times 10^{-6}$).

The TwinsUK adult twin registry (TwinsUK). TwinsUK based at St. Thomas' Hospital in London is a volunteer cohort of over 10,000 twins from the general population[13]. Twins largely volunteered unaware of the eye studies, gave fully informed consent under a protocol reviewed by the St. Thomas' Hospital Local Research Ethics Committee and underwent full eye examination. Genotyping was carried out using three genotyping platforms from Illumina: the HumanHap 300k Duo for part of the UK Twin Cohort and the HumanHap610-Quad array for the rest of the UK Twin Cohort. Individuals were included if their genotyping success rate exceeded 95%, did not show excess or low heterozygosity (defined by the interval interval of 0.2-04). SNPs were included in the imputation if they had a genotype success rate of at least 0.95 if their minor allele frequency was superior to 0.005 and at least 0.99 if their MAF was 0.01-0.05. Only SNPs that were within Hardy-Weinberg equilibrium ($p>10^{-04}$) and had a minor allele frequency of 0.04 or above were regressed.

South London Case Control Study (SLCCS). SLCCS is a case-control study, aimed at recruiting a replication sample for studies of the genetics of age-related eye diseases in South London. Study participants were recruited from the Princess Royal University Hospital, Orpington, and St Thomas' Hospital in London. Cataract cases were defined as having significant nuclear cataract (grade >=3 on the LOCS III clinical grading system), controls were individuals without significant cataract (as assessed by treating physician, usually without LOCS III grades) and attending the eye department for other reasons (e.g. patients with glaucoma, age-related macular degeneration or other conditions). The

SLCCS had no exclusion criteria on the grounds of age, gender, or ethnic origin, but for this study subjects identified as being of Northern European origin on principal component analyses were included.

2. Comparison of cataract grading systems

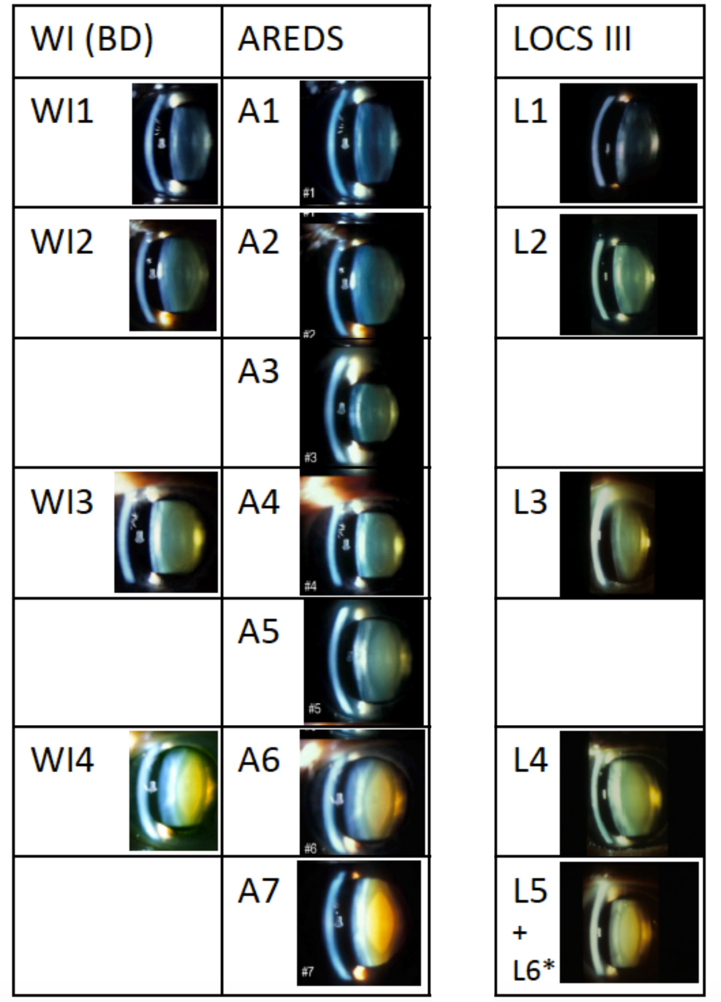

A comparison between the three grading systems (Wisconsin [WI], Age-related Eye Disease Study [AREDS] and LOCS III systems) used in the replication cohorts. The AREDS grading system included 4 standard lens photographs from the Wisconsin system, while the LOCS III used an entirely different set of lens photographs. Therefore, direct comparison among the three systems is challenging. An approximate conversion algorithm for conversion between LOCS III and Wisconsin System was proposed, yet the

algorithm provides only general ways to pool and compare cataract prevalence at population levels, rather than to concert grading at individual levels.[15] Comparison between subjective and objective grading has been published previously.[16]

References

1. Age-Related Eye Disease Study Research Group. A randomized, placebo-controlled, clinical trial of high-dose supplementation with vitamins C and E and beta carotene for age-related cataract and vision loss: AREDS report no. 9. *Arch. Ophthalmol. Chic. Ill 1960* **119,** 1439–1452 (2001).
2. Age-Related Eye Disease Study Research Group. A randomized, placebo-controlled, clinical trial of high-dose supplementation with vitamins C and E, beta carotene, and zinc for age-related macular degeneration and vision loss: AREDS report no. 8. *Arch. Ophthalmol. Chic. Ill 1960* **119,** 1417–1436 (2001).
3. Campbell, J. A. & Palit, C. D. Total digit dialing for a small area census by phone. *Proc. Surv. Res. Methods Sect. Am. Stat. Assoc.* 549 (1988).
4. Jonas, J. B., Xu, L. & Wang, SUPPLEMENTAL. X. The Beijing Eye Study. *Acta Ophthalmol. (Copenh.)* **87,** 247–261 (2009).
5. Age-Related Eye Disease Study Research Group. The age-related eye disease study (AREDS) system for classifying cataracts from photographs: AREDS report no. 4. *Am. J. Ophthalmol.* **131,** 167–175 (2001).
6. Zhang, J. S. *et al.* Five-Year Incidence of Age-Related Cataract and Cataract Surgery in the Adult Population of Greater Beijing. *Ophthalmology* **118,** 711–718 (2011).
7. Klein, B. E., Klein, R., Linton, K. L., Magli, SUPPLEMENTAL. L. & Neider, M. SUPPLEMENTAL. Assessment of cataracts from photographs in the Beaver Dam Eye Study. *Ophthalmology* **97,** 1428–1433 (1990).
8. Tan, A. C. S. *et al.* Cataract Prevalence Varies Substantially with Assessment Systems: Comparison of Clinical and Photographic Grading in a Population-Based Study. *Ophthalmic Epidemiol.* **18,** 164–170 (2011).
9. Lavanya, R. *et al.* Methodology of the Singapore Indian Chinese Cohort (SICC) Eye Study: Quantifying ethnic variations in the epidemiology of eye diseases in Asians. *Ophthalmic Epidemiol.* **16,** 325–336 (2009).
10. Foong, A. SUPPLEMENTAL. P. *et al.* Rationale and methodology for a population-based study of eye diseases in Malay people: The Singapore Malay eye study (SiMES). *Ophthalmic Epidemiol.* **14,** 25–35 (2007).
11. Vithana, E. N. *et al.* Collagen-related genes influence the glaucoma risk factor, central corneal thickness. *Hum. Mol. Genet.* **20,** 649–658 (2011).
12. Khor, C. C. *et al.* Genome-wide association studies in Asians confirm the involvement of ATOH7 and TGFBR3, and further identify CARD10 as a novel locus influencing optic disc area. *Hum. Mol. Genet.* **20,** 1864–1872 (2011).
13. Spector, T. D. & Williams, F. M. K. The UK Adult Twin Registry (TwinsUK). *Twin Res. Hum. Genet. Off. J. Int. Soc. Twin Stud.* **9,** 899–906 (2006).
14. Louttit, M. D. *et al.* A multicenter study to map genes for Fuchs endothelial corneal dystrophy: baseline characteristics and heritability. *Cornea* **31,** 26–35 (2012).
15. Wong W. L. e*t al.* Cataract Conversion Assessment Using Lens Opacity Classification System III and Wisconsin Cataract Grading System. *Invest Ophthalmol Vis Sci*. **54**, 280-287 (2013).
16. Hammond, C. J. *et al.* Genetic and Environmental Factors in Age-Related Nuclear Cataracts in Monozygotic and Dizygotic Twins. *N Engl J Med*. **342**, 1786-1790 (2000).

Supplementary Acknowledgements

The Age-Related Eye Disease Study (**AREDS**) **1a1b** and Fuchs' Endothelial Corneal Dystrophy (**FECD**) Controls were supported by the National Eye Institute (grants R01EY16482, R21EY015145, and P30EY11373) and by Research to Prevent Blindness and the Ohio Lions Eye Research Foundation. The investigators gratefully acknowledge the role of the clinical co-ordinators and investigators who collected data on FECD cases and controls. Individual investigators and sites are listed in the first publication of the FECD study[14]. Data for the AREDS1a and 1b studies was downloaded from dbGaP for analysis under a National Eye Institute data use agreement.

AREDS1c was supported by contracts from National Eye Institute/National Institutes of Health, Bethesda, MD, with additional support from Bausch & Lomb Inc., Rochester, NY. The genotyping costs were supported by the National Eye Institute (R01EY020483 to D.S.) and some of the analyses were supported by the Intramural Research Program of the National Human Genome Research Institute, National Institutes of Health, USA. AREDS acknowledges Frederick Ferris, National Eye Institute, National Institutes of Health, Bethesda, MD; and the Center for Inherited Disease Research, Baltimore, MD where SNP genotyping was carried out. The investigators gratefully acknowledge the advice and guidance of Hemin Chin of the National Eye Institute.

The Beaver Dam Eye Study (BDES) was supported by grant EY06594 from the National Institute of Health as well as Senior Scientific Investigator Award (to BEKK) and an unrestricted grant (to the University of Wisconsin Department of Ophthalmology and Visual Sciences) from Research to prevent Blindness. The Michigan study was supported by the National Eye Institute (EY0022005) and the National Human Genome Research Institute (HG006513 HG007022), Foundation Fighting Blindness and National Institutes of Health/National Eye Institute Grant-EY016862.

The Beijing Eye Study (BES) was supported by National Natural Science Foundation of China (grants 81170890 and 81041018) and the National Science Fund of Beijing government (grants 7092021 and 7112031).

The Blue Mountains Eye Study (BMES) was supported by the Australian National Health & Medical Research Council (NH&MRC), Canberra Australia (974159, 211069, 457349, 512423, 475604, 529912); the Centre for Clinical Research Excellence in Translational Clinical Research in Eye Diseases; NH&MRC research fellowships (358702, 632909 to J.J.SUPPLEMENTAL); and the Wellcome Trust, UK as part of Wellcome Trust Case Control Consortium 2 (A. Viswanathan, P. McGuffin, P. Mitchell, F.

Topouzis, P. Foster) for genotyping costs of the entire BMES population (085475B08Z, 08547508Z, 076113).

We acknowledge the Blue Mountains Eye Study GWAS team for their contribution to the GWAS data: Jie Jin Wang[1], Paul Mitchell[1], Elena Rochtchina[1], Ananth C. Viswanathan[2], Tien SUPPLEMENTAL. Wong[3,4], Jing Xie[3], Xueling Sim[5], Michael Inouye[6], Elizabeth G. Holliday[7,8], John Attia[7,8], Rodney J. Scott[8,9,10], Paul N, Baird [3]. 1) Centre for Vision Research, Department of Ophthalmology and Westmead Institute for Medical Research, University of Sydney, NSW Australia; 2) NIHR Biomedical Research Centre for Ophthalmology, Moorfields Eye Hospital NHS Foundation Trust and UCL Institute of Ophthalmology, London EC1V 2PD, UK; 3) Centre for Eye Research Australia, University of Melbourne, Royal Victorian Eye and Ear Hospital, Melbourne, Australia; 4) Singapore Eye Research Institute, Singapore National Eye Centre, National University of Singapore, Singapore; 5) National University of Singapore, Singapore; 6) Medical Systems Biology, Department of Pathology and Department of Microbiology & Immunology, The University of Melbourne, Parkville 3010, Victoria, Australia; 7) School of Medicine and Public Health, University of Newcastle, Newcastle, Australia; 8) Hunter Medical Research Institute, Newcastle, Australia; 9) The Centre for Information Based Medicine and the School of Biomedical Sciences and Pharmacy University of Newcastle, Newcastle, Australia; 10) The Division of Genetics, Hunter Area Pathology Service, John Hunter Hospital, Newcastle, Australia.

The Rotterdam Study is funded by Erasmus Medical Center and Erasmus University, Rotterdam, Netherlands Organization for the Health Research and Development (ZonMw), the Research Institute for Diseases in the Elderly (RIDE), the Ministry of Education, Culture and Science, the Ministry for Health, Welfare and Sports, the European Commission (DG XII), the Municipality of Rotterdam, Vidi 91796357 from the Netherlands Organization of Scientific Research (Klaver), grant Uitzicht 2014-38 (Klaver) and 2012-45 (Klaver) and Topcon Europe. Uitzicht grants are financed by Macula Fonds, ODAS stichting, Landelijke Stichting voor Blinden en Slechtzienden and Algemene Nederlandse Vereniging ter Voorkoming van Blindheid. The authors are grateful to the study participants, the staff from the Rotterdam Study and the participating general practitioners and pharmacists.

The Singapore Chinese Eye Study (SCES), Singapore Malay Eye Study (SiMES) and Singapore Indian Eye Study (SINDI) were supported by the National Medical Research Council (NMRC), Singapore (grants 0796/2003, 1176/2008,

1149/2008, STaR/0003/2008, 1249/2010, CG/SERI/2010, CIRG/1371/2013, and CIRG/1417/2015), and Biomedical Research Council, Singapore (08/1/35/19/550 and 09/1/35/19/616). Ching-Yu Cheng is supported by an award from NMRC (CSA/033/2012). E-Shyong Tai is also supported by an award from NMRC (CSA/008/2009).

The Immunofluorescence confocal microscopy work was supported by grants from the National Medical Research Council (NMRC/TCR/002-SERI/2008 and NMRC/CBRG/0032/2013)

The **TwinsUK** study was funded by: the Wellcome Trust, the Guide Dogs for the Blind Association and the European Community's Seventh Framework Programme (FP7/2007-2013). The study also received support from the National Institute for Health Research(NIHR)–funded BioResource, Clinical Research Facility, and Biomedical Research Centre based at Guy's and St. Thomas' NHS Foundation Trust in partnership with King's College London. **The South London Case Control Study (SLCCS)** was supported by an NIHR Senior Research Fellowship awarded to CJ Hammond. E.SUPPLEMENTAL-D.: Funding − Biotechnology and Biological Sciences Research Council and the London Interdisciplinary Doctoral Program.
