# Supplementary material for: Common variants in SOX-2 and congenital cataract genes contribute to age-related nuclear cataract
Source: Commun Biol. 2020 Dec 11;3:755. doi: 10.1038/s42003-020-01421-2 (PMC7733496; doi:10.1038/s42003-020-01421-2)
Supplement: Supplementary file 2 — Description of Additional Supplementary Files [file 42003_2020_1421_MOESM2_ESM.pdf]

Description of additional supplementary files

File name: Supplementary Data 1

Description: eQTL data (GTEx, TwinsUK expression data) for variants associated with age-related nuclear cataract (P<1x10-6) in the combined analysis (Discovery phase), as well as the *GJA3* and *CRYAB* loci.

Only the most strongly associated variant per locus is reported. Variants in both are variants that are either the top eQTL for a given gene in a tissue or in a strong LD (r2>0.8) with the top eQTL for that gene. Chr – chromosome; SNP – SNP rs number; P – P-value from the combined analysis (Discovery phase): N – number of hits; eQTL P – P-value for the correlation between SNP allele and transcript levels; Top eQTL for gene – the strongest eQTL for the gene in the respective tissue; LD – linkage disequilibrium parameters (r2 and D') for the GWAS and the top eQTL SNPs were calculated against the 1000 Genomes LD data using SNAP (https://Supplemental.broadinstitute.org/mpg/snap/ldsearchpw.php), or LD calculator (https://caprica.genetics.kcl.ac.uk/~ilori/ld_calculator.php) in the cases where the two SNP were further apart than the SNAP distance limit of 500kb; LCL – lymphoblastoid cell line; ** The most strongly associated SNP (rs10502150, P=2.2x10-6) at CRYAB locus in the European individual only (Phase 1) is in perfect LD (r2=0.95/D'=1.0) with the top SNP for CRYAB (rs11214027) in fat.

File name: Supplementary Data 2

Description: Variants in proximity to 47 genes linked to congenital cataract were nominally associated with ARNC in our analysis.

Source – database or literature search (LS), listing the gene as a congenital cataract gene: OMIM – Online Mendelian Inheritance in Human, Cat-Map – Cataract Map; ClinVar – Clinical Variants; Chr – chromosome; SNP - SNP rs number; Beta - effect size and its standard error (SE); P – P-value from Discovery phase. Associations within gene refers to the longest transcript; locus - region between two hot-spots of recombination (recombination rates ≥ 40) or a 1 mega base (MB) window, whichever was smaller; Dist. - distance between variant and the beginning or end of the longest transcript for that gene (in kilo bases). For a gene to be associated (denoted in bold) it had to have more than one variant within the longest open reading frame ± 100kb (recombination rates ≤ 40) that was associated with nuclear cataract at nominal significance (P<0.05).

File name: Supplementary Data 3

Description: Summary statistics for the genome-wide association study.
